# Benmelstobart plus anlotinib in patients with EGFR-positive advanced NSCLC after failure of EGFR TKIs therapy: a phase I/II study

**DOI:** 10.1038/s41392-024-01982-2

**Published:** 2024-10-10

**Authors:** Meiqi Shi, Ping Chen, Bin Cui, Yuanhu Yao, Juanyi Wang, Tong Zhou, Li Wang

**Affiliations:** 1 Department of Medical Oncology, Jiangsu Cancer Hospital and Jiangsu Institute of Cancer Research and The Affiliated Cancer Hospital of Nanjing Medical University, Nanjing, China; 2 Department of Oncology, Yancheng NO.1 People’s Hospital, Yancheng, China; 3 Department of Oncology, Ji’nan Zhangqiu District People’s Hospital, Zhangqiu, China; 4 Department of Radiotherapy, The Affiliated Hospital of Xuzhou Medical University, Xuzhou, China; 5 Department of Oncology, Hanzhong 3201 Hospital, Hanzhou, China; 6 Department of Oncology, Changzhou Cancer Hospital, Changzhou, China

**Keywords:** Cancer therapy, Cancer

## Abstract

The effect of immune‐based therapies on patients with epidermal growth factor receptor (EGFR)-positive advanced non-small cell lung cancer (NSCLC) resistant to EGFR tyrosine kinase inhibitor (TKI) therapy remains unclear. The ALTER-L038 study aimed to evaluate efficacy and safety of a chemotherapy-free combination of benmelstobart, an anti-programmed cell death ligand 1 antibody, and anlotinib, a small-molecule multi-target anti-angiogenic TKI, in EGFR-positive advanced NSCLC patients who progressed after EGFR TKI therapy. Patients were enrolled in a phase I/II study. In phase I (dose-escalation), patients received anlotinib (8, 10, 12 mg) plus benmelstobart (1200 mg). Recommended phase II dose, determined during phase I, was used in phase II dose-expansion cohort. Primary endpoints were maximum tolerable dose in phase I and progression-free survival (PFS) in phase II. At the data cutoff date (March 10, 2024), 55 patients were enrolled in phase II dose-expansion cohort. Median PFS of patients included in phase II cohort was 9.0 months, median overall survival was 28.9 months, objective response rate was 25.5%, disease control rate was 87.3%, and median duration of response was 19.8 months. Incidence of grade ≥3 treatment-related adverse events in study population was 25.5% (14/55), whereas grade ≥3 immune-related adverse events occurred in 10.9% (6/55) of patients. Benmelstobart plus anlotinib showed promising anti-tumor efficacy with tolerable safety profile, supporting the value of further development of this convenient chemotherapy-free regimen for patients with EGFR-positive advanced NSCLC who progressed after EGFR TKI therapy. Trial Registration: ChiCTR1900026273.

## Introduction

A high epidermal growth factor receptor (EGFR) mutation rate of 51.4% has been reported in Asian patients with advanced non-small cell lung cancer (NSCLC).^1^ Platinum-based chemotherapy with or without bevacizumab has become the standard treatment for patients with EGFR-positive advanced NSCLC resistant to EGFR tyrosine kinase inhibitors (TKIs).^2,3^ Previous studies have shown the preliminary efficacy of immunochemotherapy with or without bevacizumab in this population.^3^ Considering the high toxicity and poor treatment compliance of chemotherapy, there remains an unmet clinical need for a safer, more convenient, and comparably effective chemotherapy-free regimen for patients with NSCLC after TKIs resistance.^4^

A phase I study on a first-line chemotherapy-free regimen (sintilimab plus anlotinib) presented at the 2019 World Conference on Lung Cancer showed anti-tumor efficacy (objective response rate [ORR], 77.3%; 6-month progression-free survival [PFS], 93.8%),^5,6^ indicating the synergy of immunotherapy and anti-angiogenic therapy in patients with EGFR-negative advanced NSCLC.^7^ The median PFS (mPFS) of 15 months observed in the updated analysis supports the value of this chemotherapy-free regimen as a potential treatment option,^6^ comparable to that of immunochemotherapy (mPFS, 6–8 months).^8–11^ Inspired by the regimen of immune checkpoint inhibitors (ICIs) and anti-angiogenic agents, we designed a chemotherapy-free combination therapy for patients with EGFR-positive advanced NSCLC.

Benmelstobart (also known as TQ-B2450), a novel humanized anti-programmed cell death ligand 1 (PD-L1) antibody, has shown preliminary efficacy in patients with advanced solid tumors, including NSCLC (ORR, 32.8%; disease control rate [DCR], 81.8%).^12^ Anlotinib, an oral multi-target TKI, has been approved for the treatment of NSCLC in China.^6,13,14^ In the phase I/II ALTER-L038 study, the efficacy and safety of the chemotherapy-free combination therapy comprising benmelstobart and anlotinib were evaluated in patients with EGFR-positive advanced NSCLC following resistance to EGFR-TKIs.

## Results

### Patient characteristics

**Fig. 1:**
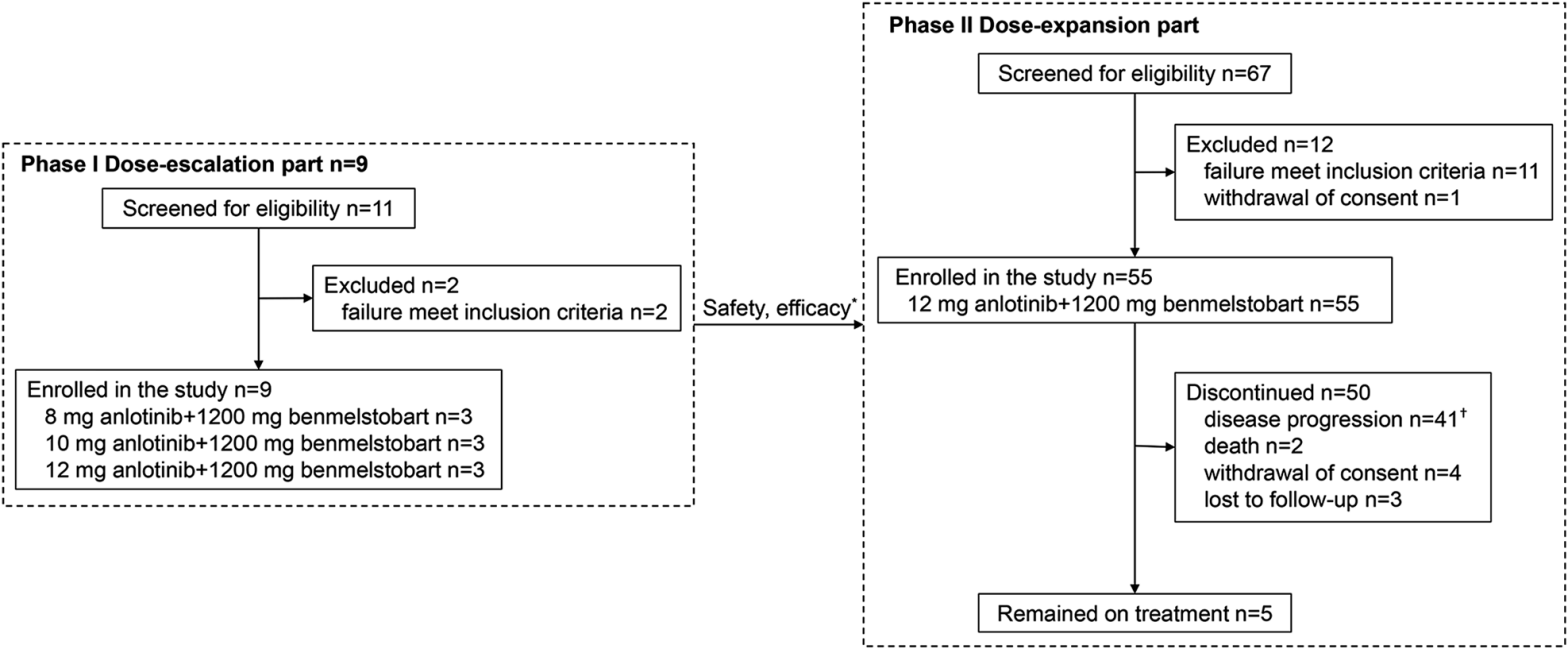
Trial design and CONSORT diagram. ^*^The dose used in the phase II dose-expansion study was based on the safety profile and efficacy observed in the phase I dose-escalation study. ^†^Among the 41 patients with disease progression, 40 exhibited imaging progression, while 1 experienced clinical progression

During phase I (November 2019–June 2020), nine patients received anlotinib (three dose levels) plus benmelstobart (Fig. 1). Between August 26, 2020, and October 18, 2022, 67 patients were screened, and 55 were enrolled in the phase II dose-expansion cohort. Twelve patients (11 failed to meet the inclusion criteria [Supplementary Table S1], and one withdrew consent) were excluded from the study. At the time of data cutoff (March 10, 2024), 50 (90.9%) of the 55 patients had discontinued the study, and 5 (9.1%) remained on therapy. The reasons for discontinuation were disease progression (*n* = 41), death (*n* = 2), withdrawal of consent (*n* = 4), loss to follow-up (*n* = 3).

Supplementary Table S2 provides details on the demographic characteristics of patients in the dose-escalation phase. The demographic characteristics of patients in phase II are presented in Table 1. The median age of the 55 patients was 64 (range, 49–73) years. At baseline, most patients had an Eastern Cooperative Oncology Group performance status (ECOG PS) of 1 (44/55, 80%). The majority (52/55; 94.5%) of patients were diagnosed with stage IV disease at the time of study entry. The EGFR mutation types in the 55 patients included exon 19 deletion (27/55; 49.1%), exon 21 Leu858Arg (21/55; 38.2%), and other mutations (6/55; 11%). Fourteen patients (14/55; 25.5%) experienced brain metastases.

**Table 1:** Baseline characteristics of the patients in the phase II study

	Total (*n* = 55)
**Age-years, median (range)**	64 (49–73)
**Men,** ***n*** **(%)**	17 (30.9)
**ECOG PS,** ***n*** **(%)**	
0	11 (20.0)
1	44 (80.0)
**Smoking status,** ***n*** **(%)**	
Never	50 (90.9)
Former	4 (7.3)
Current	1 (1.8)
**Disease stage,** ***n*** **(%)**	
IIIB	3 (5.5)
IV	52 (94.5)
**Number of organs involved in metastases**	
≤2	50 (90.9)
>2	5 (9.1)
**Previous systemic chemotherapy,** ***n*** **(%)**	13 (23.6)
Adjuvant chemotherapy	12 (21.8)
Neoadjuvant and adjuvant chemotherapy	1 (1.8)
**mSLD,** ***n*** **(%)**	
<37.4 mm	27 (49.1)
≥37.4 mm	28 (50.9)
**EGFR mutations, n (%)**	
Exon 19 deletion	27 (49.1)
Exon 21 Leu858Arg	21 (38.2)
Others^a^	6 (10.9)
Not available^b^	1 (1.8)
**Generation of previous EGFR TKI therapy, n (%)**	
First or second	21 (38.2)
First or second, followed by the third	26 (47.3)
Third	8 (14.6)
**T790M mutation,** ***n*** **(%)**	
Yes	24 (43.6)
No	20 (36.4)
Unknown	11 (20.0)
**Brain metastases,** ***n*** **(%)**	14 (25.5)
**Previous EGFR TKI treatment time,** ***n*** **(%)**	
≤1 year	9 (16.4)
1–2 years	19 (34.6)
>2 years	27 (49.1)

*ECOG PS* Eastern Cooperative Oncology Group Performance Status, *mSLD* median summation of longest diameter (37.4 mm in this study), *EGFR* Epidermal growth factor receptor, *TKI* tyrosine kinase inhibitor

^a^Other EGFR mutations include G719X, exon 18 p.G719A, exon 20 insertions, and A750del

^b^This patient only provided a previous T790M mutation test report

### Efficacy

**Fig. 2:**
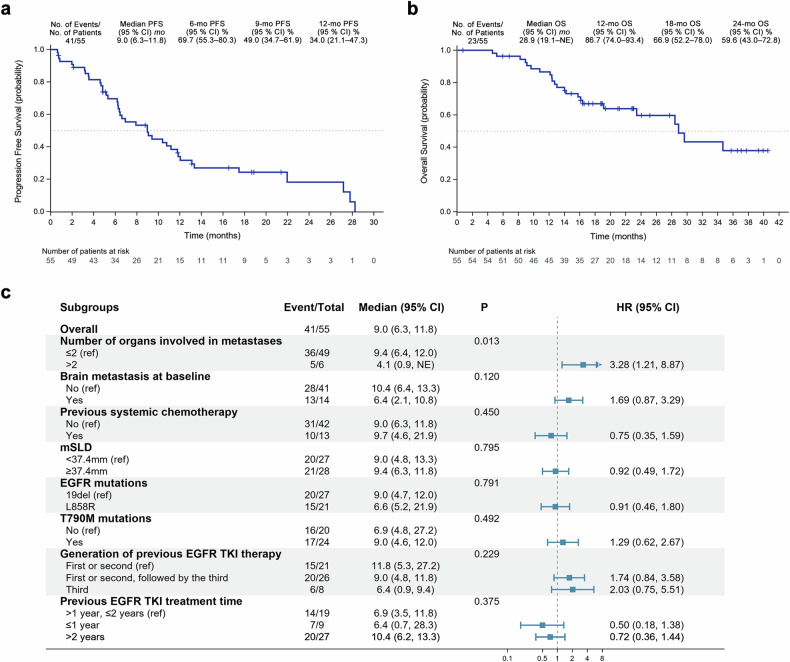
Progression-free survival (PFS) (**a**), overall survival analyses (**b**), and forest plot of median PFS (**c**) in phase II of the study. PFS progression-free survival, OS overall survival. CI confidence interval, EGFR epidermal growth factor receptor, TKI tyrosine kinase inhibitor, mSLD, median summation of longest diameter (37.4 mm in this study); NE not estimable

In phase II, the median follow-up was 22.8 (range, 18.9–27.7) months for 55 patients. The Kaplan–Meier analyses of the PFS and overall survival (OS) curves are shown in Fig. 2. Overall, 41 PFS events were observed, and the mPFS was 9.0 (95% confidence intervals [CIs], 6.3–11.8) months (Fig. 2a). The estimated probabilities of PFS at 6, 9, and 12 months were 69.7% (95% CI, 55.3%–80.3%), 49.0% (95% CI, 34.7%–61.9%), and 34.0% (95% CI, 21.1%–47.3%), respectively. A total of 23 deaths were reported (OS events), and the estimated median OS (mOS) was 28.9 (95% CI, 19.1–not estimable [NE]) months (Fig. 2b). The 12-month and 18-month OS rates of the overall population were 86.7% (95% CI, 74.0%–93.4%) and 66.9% (95% CI, 52.2%–78.0%), respectively.

As illustrated in the forest plot (Fig. 2c), the mPFS was further analyzed in subgroups defined by baseline characteristics. The mPFS of patients with T790M mutations was 9.0 (95% CI, 4.6‒12.0) months. The patients without T790M mutations achieved a mPFS of 6.9 (95% CI, 4.8‒27.2) months.

In the full analysis population of phase II, the ORR was 25.5% (95% CI, 14.7%–39.0%), and the DCR was 87.3% (95% CI, 75.5%–94.7%), with a median duration of response (DoR) of 19.8 (95% CI, 7.7–26.2) months (Table 2 and Supplementary Fig. S1). Detailed subsequent treatment information for patients is presented in Supplementary Table S3.

**Table 2:** Anti-tumor activity in patients in the phase II study

	Total (*n* = 55)
**Best response,** ***n*** **(%)**	
Complete response	0
Partial response	14 (25.5)
Stable disease	34 (61.8)
Progressive disease	6 (10.9)
Not estimable	1 (1.8)
**ORR, % (95% CI)**	25.5 (14.7, 39.0)
**DCR, % (95% CI)**	87.3 (75.5, 94.7)
**Median DoR, months (95% CI)**	19.8 (7.7, 26.2)
**Median TTR, months (range)**	2.1 (0.5–3.5)

*CI* confidence interval, *ORR* Objective response rate, *DCR* Disease control rate, *DoR* Duration of response, *TTR* Time to response

### Safety

In phase I, no dose-limiting toxicities (DLTs) were observed, and no maximum tolerable dose (MTD) was determined. The most common grade 1–2 adverse events (AEs) were hand-foot syndrome (33.3%) and proteinuria (22.2%). Only two patients reported grade 3 AEs, which included hypertension.

Among the 55 patients in phase II, treatment-related AEs (TRAEs) of any grade occurred in 51 of the (92.7%; Table 3) patients; the TRAEs predominantly included hypertension (45.5%), hand-foot syndrome (38.2%), and proteinuria (27.3%). Fourteen (25.5%) of the 55 patients experienced grade ≥3 TRAEs. Four (4/55; 7.3%) patients reported serious treatment-related AEs (SAEs). TRAEs led to dose interruptions and dose reductions in 10 (10/55; 18.2%) and 7 (7/55; 12.7%) patients, respectively. There were no deaths attributed to TRAEs in the overall population.

**Table 3:** Treatment-related and immune-related adverse events in the phase II study

	Total (*n* = 55)	
	Any grade	≥Grade 3
**TRAE**	51 (92.7)	14 (25.5)
**TRAEs leading to reductions**	7 (12.7)	2 (3.6)
**TRAEs leading to interruptions**	10 (18.2)	3 (5.5)
**TRAE leading to death**	0	0
**Frequent TRAEs (≥5% incidence)**		
Hypertension	25 (45.5)	6 (10.9)
Hand-foot syndrome	21 (38.2)	2 (3.6)
Proteinuria	15 (27.3)	1 (1.8)
Fatigue	9 (16.4)	0
Hypercholesteremia	9 (16.4)	0
Cough	9 (16.4)	0
Hypothyroidism	8 (14.6)	0
Anorexia	8 (14.6)	0
Hypertriglyceridemia	6 (10.9)	2 (3.6)
Hoarseness	6 (10.9)	0
Diarrhea	5 (9.1)	1 (1.8)
Hyperthyroidism	5 (9.1)	0
Mucositis oral	5 (9.1)	0
Weight loss	4 (7.3)	0
White blood cell decreased	3 (5.5)	0
Positive fecal occult blood tests	3 (5.5)	0
Nausea	3 (5.5)	0
Lung infection	3 (5.5)	0
Liver injury	3 (5.5)	0
Periarthritis of shoulder	3 (5.5)	1 (1.8)
Rash	3 (5.5)	0
Blood bilirubin increased	3 (5.5)	0
Platelet count decreased	3 (5.5)	1 (1.8)
**irAEs**	38 (69.1)	6 (10.9)
**Frequent irAEs (≥10% incidence)**		
Fatigue	9 (16.4)	0
Hypothyroidism	7 (12.7)	0
Cough	6 (10.9)	0
Hoarseness	6 (10.9)	0
Anorexia	6 (10.9)	0

Data are expressed as *n* (%)

*TRAE* treatment-related adverse event, *irAE* immune-related adverse event

Furthermore, immune-related adverse events (irAEs) were reported in 38 of 55 (69.1%) patients; fatigue (16.4%) and hypothyroidism (12.7%) were the most frequently reported irAEs. Among the 55 patients, only 6 (10.9%) experienced grade ≥3 irAEs.

## Discussion

This is the first study on a chemotherapy-free regimen conducted in Chinese patients with EGFR-positive advanced NSCLC who progressed after EGFR TKI therapy. Benmelstobart plus anlotinib therapy exhibited good efficacy and low toxicity, representing a promising chemotherapy-free immunotherapy option for this population.

Various combinations of immunotherapies have been assessed in patients with EGFR-positive advanced NSCLC who progressed after EGFR-TKIs. Compared with the effect of chemotherapy alone, immunotherapy plus chemotherapy failed to demonstrate significant benefits in the CheckMate-722 (mPFS, 5.6 vs. 5.4 months, respectively; *p* = 0.053) and Keynote-789 (mPFS, 5.6 vs. 5.5 months, respectively; *p* = 0.0122) studies.^15,16^ The Orient-31 study, involving immunotherapy, chemotherapy, and IBI305 (bevacizumab biosimilar), showed a better mPFS compared to that observed with chemotherapy alone (6.9 vs. 4.3 months, respectively; *p* < 0.0001), but the study reported a notably high incidence (54.7%) of grade ≥3 TRAEs, raising concerns about safety in clinical application and subsequent treatment selection.^17^ Recent ATTLAS (mPFS, 8.47 months; grade ≥3 TRAEs, 35.1%) and HARMONi-A (mPFS, 7.1 months; grade ≥3 TEAEs, 61.5%) studies have shown similar efficacy and safety profiles.^18,19^ Despite the superior mPFS observed for the quadruple combination (atezolizumab plus bevacizumab and chemotherapy; ABCP regimen) arm than that for the triple combination (bevacizumab plus chemotherapy; BCP regimen) arm (10.2 vs. 6.9 months, respectively) in the IMpower150 study,^3^ the mPFS benefits (8.5 and 8.3 months) of the ABCP regimen could not be further validated in the IMpower151 study.^20^ Furthermore, among 80 patients in the real-world BACH-NET study, 22 received the ABCP regimen, with an ineligibility rate of 80%, mostly because of poor PS and comorbidities, and the patients had a lower mPFS compared with that in the IMpower150 study (5.7 vs. 10.2 months, respectively),^21^ suggesting that the quadruple combination was not feasible for most patients, with limited clinical acceptance and low efficacy. Besides, as demonstrated by the promising clinical efficacy (mPFS, 7.1–11.5 months), antibody-drug conjugates, such as Dato-DXd, HER3-DXd, SKB264, and BL-B01D1, and bispecific antibody plus chemotherapy (e.g., LACP regimen [lazertinib, amivantamab, carboplatin, pemetrexed]) represent the recent therapeutic advances.^22–26^ However, cumulative toxicity of chemotherapy drugs hinders long-term administration and compromises the benefits of the combination regimens.^27^ In this treatment landscape, the ALTER-L038 study met its primary endpoint, achieving a mPFS of nine months with a tolerable safety profile in patients with EGFR-positive advanced NSCLC who progressed after EGFR TKI, indicating the potential for a new chemotherapy-free immunotherapy in this population.

The ORR observed with benmelstobart plus anlotinib therapy was numerically lower than that observed with immunochemotherapy with or without bevacizumab (25.5% vs. 33.1%–43.9%, respectively).^17^ Notably, we observed a DCR of 87.3% and a mDoR of 19.8 months, indicating the durability of response in responders. Given a mDoR range of 6.3 to 11.3 months in similar populations (as observed in CheckMate-722, Keynote-789, ORIENT-31, and IMpower151), the mDoR in our study appears promising, considering the small sample size and single-arm design.^15–17,20^ Besides, this study preliminarily showed a mOS of 28.9 months, whereas previous studies have reported an mOS ranging from 17.1 to 21.1 months.^17–19^ Poor PS and intolerance to limited post-study therapies after progression (grade ≥3 TRAEs, 35.1–56%) may lead to an OS of 20.6–21.1 months due to the lack of extensibility in patients who received the quadruple combination.^17,18^ The encouraging OS values observed in our study may be related to good administration of various post-study therapies.^28,29^ The high degree of efficacy was not only because of the synergy between the ICI and anti-angiogenic therapies,^7^ but also caused by the single-agent activity of the multi-target TKI anlotinib, which is different from the activity of bevacizumab.^13,14^ Consequently, a combined anlotinib regimen may achieve substantial efficacy in patients with NSCLC, as demonstrated by the promising survival data obtained in several studies (anlotinib plus EGFR-TKI therapy continuation after gradual or local progression: mPFS, 9.2 months; anlotinib plus sintilimab: mPFS, 15 months).^6,30^ Furthermore, the advantages of chemotherapy-free combination therapy with benmelstobart plus anlotinib include favorable safety profiles (grade ≥3 TRAEs, 25.5%; grade ≥3 irAEs, 10.9%) compared to those of chemotherapy-containing regimens. In this study, although results should be interpreted with caution due to the limited sample size, patients with the T790M mutation achieved a numerically higher mPFS (9.0 vs. 6.9 months), which may be attributed to the potent cytotoxicity exerted by anlotinib on the T790M-mutant cell line.^31^ Notably, significant mPFS benefits (HR, 0.24; 95% CI, 0.09‒0.59) for T790M mutation patients were observed in the HARMONi-A study,^19^ whereas such benefits were not evident in the ILLUMINATE, Orient-31, and ATTLAS studies.^17,18,32^ Consistent with the findings of the IMpower151 study,^20^ mPFS (9.0–11.8 vs. 6.4 months, respectively) improved in the subpopulation that received first-/second-generation EGFR-TKIs, regardless of subsequent third-generation EGFR-TKIs. Overall, benmelstobart plus anlotinib achieved a clinically therapeutic effect in most subpopulations, making it a promising therapy for the evolving treatment landscape.

This phase I/II study was limited by its single-arm design, small sample size, and lack of a randomized controlled design. Second, the results of subgroup analysis may be biased due to limited sample size, wide confidence intervals, and potential heterogeneity, requiring cautious interpretation. Third, the absence of candidate biomarkers (such as PD-L1 expression) analysis, and tumor microenvironment profiling limited our ability to assess the efficacy of the therapy and relationships between the study drug and tumors. It would be important to investigate tumor biomarkers in large-scale randomized trials for response to better select patients for the benmelstobart plus anlotinib therapy.

In conclusion, in the context of the attempts of numerous immunotherapy combinations in patients with EGFR-positive advanced NSCLS resistant to EGFR-TKIs, benmelstobart plus anlotinib therapy, which shows anti-tumor efficacy, low toxicity, and the convenience of oral administration, could potentially fill the gap in the treatment of this population.

## Materials and methods

### Study design and patients

ALTER-L038 (Chinese Clinical Trial Registry identifier: ChiCTR1900026273), a multicenter, single-arm, phase I/II trial, is currently being conducted in China. This study included two parts: phase I (3 + 3 dose-escalation cohorts) and phase II (dose-expansion cohorts). Written informed consent was obtained from all patients, and the study was conducted in accordance with the principles of the Declaration of Helsinki and Good Clinical Practice. This study was approved by the institutional review board and independent ethics committees at each site.

In both cohorts, the enrolled patients, aged 18–75 years, had histologically or cytologically confirmed stage IIIB–IV EGFR-positive NSCLC. Eligible patients included those with EGFR-activating mutations in whom prior EGFR-TKI therapy had failed, including first- or second-generation EGFR TKI therapy in patients without the T790M mutation and third-generation TKI therapy in patients with the T790M mutation or third-generation EGFR TKI therapy. All patients had a predicted survival of 3 months or more, an ECOG PS of 0–1, and at least one measurable lesion according to the response evaluation criteria in solid tumors (RECIST) version 1.1. Patients were excluded if they had previously undergone ICI therapy, anti-angiogenic agent therapy, ≥1 systemic chemotherapy, or allergies to anlotinib. Additional inclusion and exclusion criteria are provided in Supplementary Text S1.

### Procedures

The patients received anlotinib orally once daily on days 1–14 and benmelstobart intravenously on day 1 of a 21-day cycle. For the dose-escalation cohort, we used a standard 3 + 3 design to determine the DLT and MTD. During dose escalation, the patients received three doses of anlotinib (8, 10, and 12 mg), and benmelstobart was administered at a fixed dose of 1200 mg. DLT, defined as drug-related toxicity during the first 21-day cycle, is detailed in Supplementary Text S2. MTD was defined as the maximum dose with ≤33% risk of DLT. The dose was escalated if 0/3 or 1/6 patients experienced DLT during the first 21-day cycle. The recommended phase II dose (12 mg anlotinib and 1200 mg benmelstobart) was determined based on the safety and response data. All patients were treated until disease progression (PD), unacceptable toxicity, poor medication compliance (defined as taking less than 80% or more than 120% of the medication prescribed), consent withdrawal, initiation of another anticancer therapy (e.g., chemotherapy, targeted therapy, or biological agents), or if they were deemed ineligible for further treatment by the investigators.

Dose delays, interruptions, and discontinuations were recommended at the investigators’ discretion in events of specific toxicities. Delays in the administration of benmelstobart and anlotinib were permitted for selected toxicities. The maximum durations of dose delays were 12 and 35 days for benmelstobart and anlotinib, respectively. If the length of the dose delay exceeded the maximum duration, the treatment was discontinued. Dose reductions were not permitted for benmelstobart but were permitted for anlotinib. The anlotinib dose was reduced from 12 to 10 mg and then to 8 mg, after which discontinuation was required (toxicities leading to dose reductions of anlotinib are shown in Supplementary Table S4), whereas dose re-escalation was not permitted. For benmelstobart, modifications to the infusion speed and interruptions or discontinuations were allowed if specific infusion reactions or irAEs occurred (further information is provided in Supplementary Tables S5 and S6).

### Assessments

The tumor was assessed by the investigators using computed tomography or magnetic resonance imaging, as per RECIST version 1.1, within four weeks before the first dose (baseline), three weeks after the first therapy, and every six weeks thereafter during therapy. Patients who discontinued therapy before PD were included in the study and continued to undergo assessments at 6-week intervals until PD or administration of other anticancer therapies. After PD, the survival status and any further anticancer therapies were documented at follow-up visits every eight weeks. The investigators reported AEs and graded them according to the National Cancer Institute-Common Terminology Criteria for Adverse Events (NCI-CTCAE) version 5, throughout the therapy period and for at least 21 days after administration of the last dose of the study drug.

### Endpoints

The MTD was the primary endpoint of the dose-escalation phase. The primary efficacy endpoint of the dose-expansion phase was PFS, which was defined as the time from entry to the first documentation of tumor progression or death from any cause (whichever occurred first). The secondary efficacy endpoints of the dose-expansion phase included OS, defined as the time from entry to death from any cause; ORR, defined as the proportion of patients who showed complete response (CR) or partial response (PR); DCR, defined as the proportion of patients with CR, PR, or stable disease (SD); DoR, defined as the time from the first objective status assessment of CR/PR to progression; time to response (TTR), defined as the time from the start of therapy to the first objective tumor response; 12-month PFS rate; 12-month OS rate; and safety.

### Statistical analysis

The sample size for phase I of the study was based on the dose-escalation rules described in the study design section and not on explicit power considerations. Based on prior efficacy data obtained using carboplatin plus pemetrexed for patients with EGFR-positive NSCLC in whom EGFR TKI therapy failed, a mPFS of five months was used as a historical control to determine the sample size for the phase II trial. We hypothesized that benmelstobart plus anlotinib would have clinical benefits in this population, with an mPFS of nine months. A sample size of 54 participants was required to achieve 90% power at a significance level (α) of 0.05, with an anticipated dropout rate of 20%.

Efficacy analysis was performed in the full analysis set (FAS), which included all patients who received at least one dose of the study therapy. The safety analysis set (SAS) included all patients with safety data who had received at least one dose of the study therapy. Patients who were alive or lost to follow-up were censored at the time of last contact to estimate OS. For both PFS and DoR, data of patients who discontinued the study or started new anticancer therapy without radiographic evidence of progression were censored at the time of discontinuation or initiation of new anticancer therapy, respectively.

The demographic and clinical characteristics of the patients, safety outcomes, and tumor responses were summarized descriptively. The mPFS, mOS, DoR, and associated two-sided 95% CIs were calculated using the Kaplan–Meier method. The Clopper–Pearson method was used to calculate the two-sided 95% CIs for ORR and DCR values. Continuous variables are summarized descriptively using means, standard deviations, medians, minima, and maxima. Categorical variables are summarized descriptively using numbers and percentages. All statistical analyses were performed using SAS version 9.4 or higher.

## Supplementary information

Sigtrans_Supplementary_Materials

Clinical Study Protocol

## Data Availability

The datasets used and/or analyzed in the current study are available from the corresponding author upon reasonable request.
