# Supplementary material for: Benmelstobart plus anlotinib in patients with EGFR-positive advanced NSCLC after failure of EGFR TKIs therapy: a phase I/II study
Source: Signal Transduct Target Ther. 2024 Oct 10;9:283. doi: 10.1038/s41392-024-01982-2 (PMC11467201; doi:10.1038/s41392-024-01982-2)
Supplement: Supplementary file 1 — Sigtrans_Supplementary_Materials [file 41392_2024_1982_MOESM1_ESM.docx]

Supplementary Materials for

Benmelstobart plus anlotinib in patients with EGFR-positive advanced NSCLC after failure of EGFR TKIs therapy

Meiqi Shi, Ping Chen, Bin Cui, Yuanhu Yao, Juanyi Wang, Tong Zhou

Correspondence to: shimeiqi1963@163.com

**This PDF file includes:**

Supplementary Texts S1 to S2

Figure. S1

Tables S1 to S6

Text S1. Inclusion and exclusion criteria

**Inclusion criteria**

The inclusion criteria were:

1. Male or female, aged 18-75 years;
2. Histologically and cytologically confirmed locally advanced/advanced non-small cell lung cancer (NSCLC), assessed as stage IIIB-Ⅳ NSCLC (according to the 8th edition of the tumor–node–metastasis staging system of the American Joint Committee on Cancer), or NSCLC patients with postoperative recurrence (all types);
3. Patients with Epidermal Growth Factor Receptor mutation-positive (EGFR+) NSCLC with failure of prior EGFR tyrosine kinase inhibitors (TKIs), or T790M mutation with failure of prior 3^rd^-generation EGFR TKIs;
4. EGFR TKI should be the mainstay of the initial treatment for patients. Patients are allowed to receive ≤1 systemic chemotherapy;
5. Patients with previous only 1^st^/2^nd^-generation EGFR TKIs were required to provide genetic testing reports showing no primary or acquired T790M mutation; patients with previous 3^rd^-generation EGFR TKIs were required to provide genetic testing reports showing EGFR mutation was positive;

Treatment failure is defined as (1) the progressive disease (PD) assessed by imaging or clinical evidence during or after EGFR TKIs treatment, or withdrawal from standard treatment for intolerant adverse events (AEs). The intolerant AEs included grade Ⅳ hematologic toxicity, or grade ≥Ⅲ nonhematologic toxicity, or grade ≥Ⅱ main organ damage such as heart, liver, and kidneys per Common Terminology Criteria for Adverse Events (CTCAE) version 5.0.

Detection results from other hospitals were also acceptable. Tissue is the first choice for detection samples, if optional blood samples cannot be obtained from tissue, the detection methods can be selected by traditional genetic testing (such as immunohistochemistry, ddPCR, Arms-PCR, etc.) or high-throughput sequencing.

1. At least one measurable lesion according to Response Evaluation Criteria in Solid Tumors (RECIST) version 1.1, without prior radiotherapy in the past three months;
2. Predicted life expectancy of ≥3 months; Eastern Cooperative Oncology Group (ECOG) performance status (PS) of 0-1;
3. Recovered from damages (≤grade 1) associated with other treatments per the CTCAE version 5.0;
4. Adequate function of the important organs as evident by the following criteria:
5. Hemanalysis (no blood transfusion, and without the use of G-CSF or other hematopoietic stimulating factors within 14 days):
6. Hemoglobin (HB) ≥80g/L;
7. Absolute neutrophil count (ANC) ≥1.5×10^9/L;
8. Platelets (PLT) ≥75×10^9/L;
9. Biochemistry:
10. Total bilirubin (TBIL) ≤upper limit of normal value (ULN);
11. Alanine aminotransferase (ALT) and aspartate aminotransferase (AST) ≤1.5×ULN;
12. Creatinine (Cr) ≤1.5×ULN or creatinine clearance rate (CCr) ≥60 mL/min;
13. Urinary protein <++;
14. Coagulation function: INR and APTT ≤1.5×ULN;
15. Doppler ultrasound assessment: left ventricular ejection fraction (LVEF) ≥50%;
16. Women of childbearing potential must have adopted contraception, or have a negative serum and urine pregnancy test within 7 days before the study enrollment; women of childbearing potential and men must agree to contraception for the duration of study treatment and 8 weeks after the last dose of study treatment, or have been of sterilization;
17. Be willing and able to provide written informed consent for the trial, and comply with all aspects of the protocol.

**Exclusion criteria**

The exclusion criteria were:

1. Patients with a history of malignant tumors within 5 years before the start of treatment except for patients with cured orthotopic cervical cancer, cutaneous basal cell carcinoma, cutaneous squamous cell carcinoma, or superficial bladder cancer (Ta [non-invasive cancer], Tis [orthotopic cancer], T1 [superficially invasive]);
2. Central squamous cell lung cancer or squamous cell lung cancer with a significant pulmonary cavity;
3. Have received >1 systemic chemotherapy during previous lines of treatment (patients with neoadjuvant or adjuvant chemotherapy were allowed to be enrolled);
4. Have previously received other anti-PD-1/PD-L1 drugs or immunotherapies targeted PD-1/PD-L1;
5. Patients with severe hypersensitivity reaction after administration of other monoclonal antibodies;
6. Patients with anlotinib allergy;
7. Patients with active autoimmune disease or a history of autoimmune disease (such as, but not limited to, autoimmune hepatitis, interstitial pneumonia, enteritis, vasculitis, and nephritis; patients with asthma that needed bronchodilators for medical intervention were unable to be enrolled); patients with vitiligo, psoriasis, alopecia, and well-controlled type 1 diabetes mellitus who did not require systemic treatment were allowed to be enrolled;
8. Patients who had clinically significant thyroid dysfunction within 6 months before enrollment and whose thyroid function did not return to normal or was clinically insignificant despite medical therapy;
9. Factors that have a significant impact on oral drug absorption, such as inability to swallow, chronic diarrhea, and intestinal obstruction;
10. Patients with brain metastases with symptoms or less than 14 days of symptom control;
11. Severe comorbidities before enrollment:
12. Unstable angina and/or congestive heart failure or vascular disease within 12 months requiring hospitalization (such as aortic aneurysm requiring surgical repair or peripheral venous thrombosis), or other heart damages judged by investigators that could influence the safety assessment of study drugs (such as uncontrolled arrhythmia, myocardial infarction, or ischemia);
13. Esophagogastric varices, unhealed ulcers, uncured wounds, or fracture within 6 months;
14. History of abdominal fistula, gastrointestinal perforation, intra-abdominal abscess, or gastrointestinal bleeding within 6 months;
15. Arterial thromboembolism, grade ≥3 venous thromboembolism, transient ischemic attack (TIA), cerebral vascular accident (CVA), hypertensive crisis, or hypertensive encephalopathy within 6 months;
16. Aggravating chronic obstructive pulmonary disease (COPD) or other respiratory illness requiring hospitalization within 28 days;
17. An active lung infection and/or acute bacterial or fungal infection requiring intravenous antibiotic treatment within 28 days;
18. Clinical jaundice caused by abnormal liver function within 7 days;
19. Hypertension that was uncontrollable with a single antihypertensive drug at present (systolic blood pressure ≥160 mmHg or diastolic blood pressure ≥100 mmHg); or being treated with a combination of two or more antihypertensive drugs;
20. Major surgical operation, biopsy, or obvious traumatic injury within 28 days before enrollment;
21. Virologic testing during screening shows any of the following:
22. HBsAg positive, and HBV DNA ≥1×10^3^ copies/mL;
23. Anti-HCV positive and HCV >ULN;
24. HIV-positive;
25. Immune checkpoint inhibitors or systemic therapies were required to achieve immunosuppression (prednisone or other hormones >10 mg/d);
26. Patients with other anti-cancer therapies or participated in other clinical studies (not EGFR TKI) within 4 weeks before the first dose of the drug; patients who received therapies or participated in other clinical studies of EGFR TKI within 2 weeks before the first dose of the drug;
27. Imaging showed that the tumor had invaded the important blood vessels, or the tumor was likely to invade the important blood vessels during the subsequent study and cause massive hemorrhage judged by investigators;
28. Patients with any bleeding signs or a history of bleeding, regardless of severity, having grade ≥3 bleeding events per CTCAE, unhealed wounds, anabiosis, or fracture within 4 weeks before grouping;
29. Clinically hemoptysis (daily hemoptysis greater than 50ml) within 2 months before enrollment (defined as coughing up ≥1 teaspoon of blood, small blood clots, or coughing up blood without sputum); but patients with blood in sputum were not excluded;
30. A history of psychotropic substance abuse and cannot be abstinent, or patients with mental disturbance;
31. Have previously received antiangiogenic drugs;
32. Conditions that increased the risk associated with study participation or the study medication, and could make a patient ineligible for study in the investigator's judgment.

Text S2. Dose-limiting toxicity

DLT (dose-limiting toxicity) was defined as a drug-related toxicity occurring during the first 21 days of therapy that resulted in a grade ≥4 hematologic toxicity; grade ≥3 neutropenia with fever (absolute neutrophil count <1000/μL, fever of ≥38.5℃); grade ≥3 neutropenia lasting >7 days; grade 3 thrombocytopenia lasting >7 days accompanied by significant bleeding or requiring transfusion; grade ≥2 hepatic and renal toxic effects lasting >7 days despite symptomatic treatment; grade 2 bilirubin lasting >7 days; grade ≥3 bilirubin; grade 3 AST (aspertate aminotransferase) or ALT (alanine aminotransferase) elevation lasting >7 days; grade 4 AST or ALT; grade ≥3 non-hematologic toxicity (excluding alopecia); grade ≥2 pancreatitis; cerebral hemorrhage; grade ≥2 pulmonary hemorrhage; grade ≥2 coagulation abnormalities or bleeding lasting >7 days; grade ≥3 fatigue lasting >7 days; grade 3 hypertension, hand-foot syndrome, diarrhea, nausea, and vomiting last to next cycle despite symptomatic treatment; grade 3 laboratory values last to next cycle despite symptomatic treatment.

Figure. S1. Tumor response in patients in the phase II study


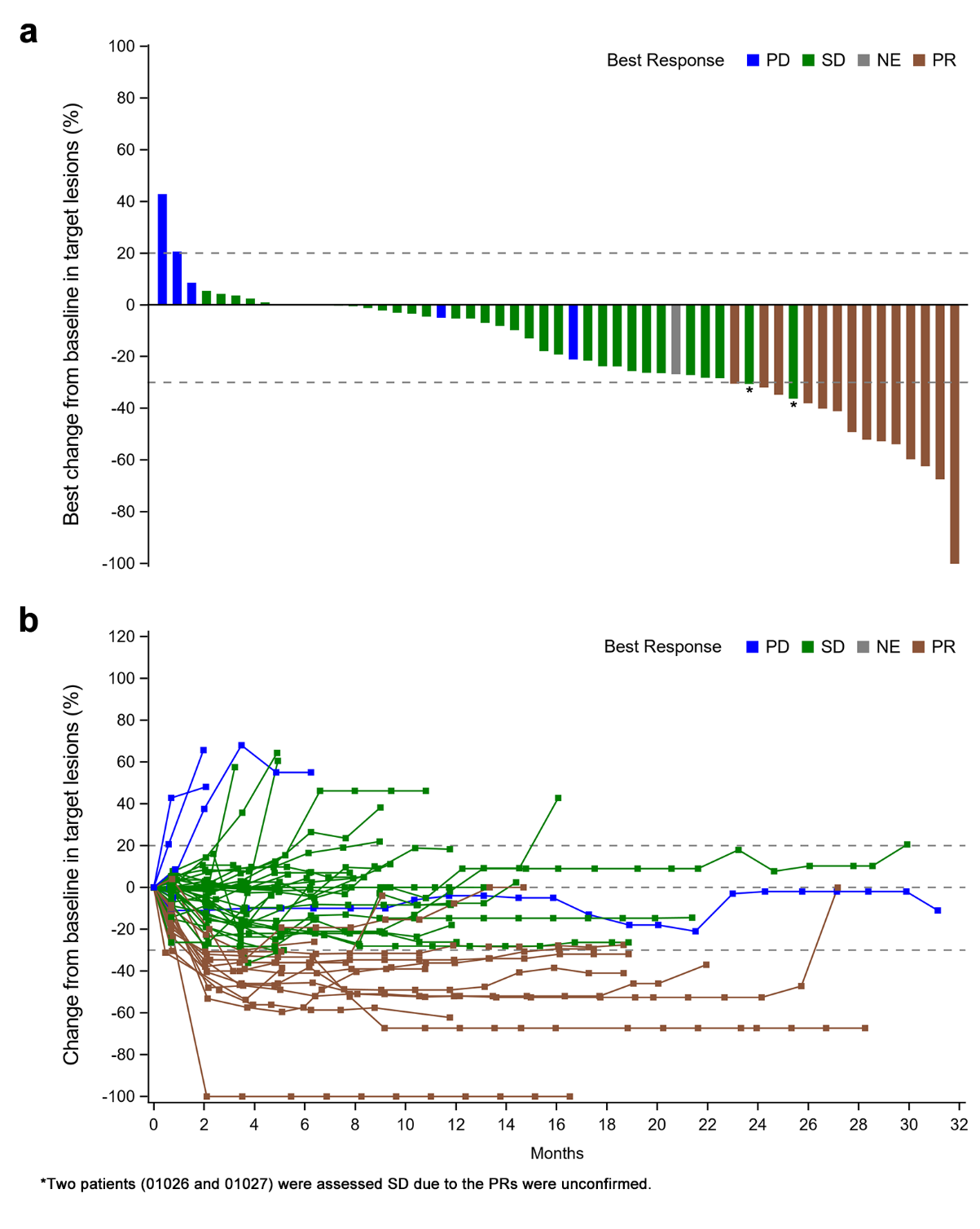


(**a**) The maximum decrease from the baseline; if the lesion increased in size, then the lowest measured increase was plotted. Each bar represents one patient. (**b**) The spider plot shows the changes over time in the target lesions of response-evaluable patients.

PR, partial response; PD, progressive disease; SD, stable disease; NE, not estimable.

Table S1. The reasons 11 patients failed to meet eligibility criteria

| **Patient** | **Reasons** |
| --- | --- |
| 01019 | Did not meet inclusion criteria number 9: Be willing and able to provide written informed consent for the trial, and comply with all aspects of the protocol.  Did not meet exclusion criteria number 21: Conditions that increased the risk associated with study participation or the study medication, and could make a patient ineligible for study in the investigator's judgment. |
| 01028 | Did not meet exclusion criteria number 13: Virologic testing during screening shows any of the following: 1) HBsAg positive, and HBV DNA ≥1×103 copies/mL; 2) Anti-HCV positive and HCV >ULN; 3) HIV-positive. |
| 01031 | Did not meet inclusion criteria number 9: Be willing and able to provide written informed consent for the trial, and comply with all aspects of the protocol. |
| 01041 | Did not meet exclusion criteria number 7: Patients with active autoimmune disease or a history of autoimmune disease (such as, but not limited to, autoimmune hepatitis, interstitial pneumonia, enteritis, vasculitis, and nephritis; patients with asthma that needed bronchodilators for medical intervention were unable to be enrolled); patients with vitiligo, psoriasis, alopecia, and well-controlled type 1 diabetes mellitus who did not require systemic treatment were allowed to be enrolled. |
| 01048 | Did not meet exclusion criteria number 10: Patients with brain metastases with symptoms or less than 14 days of symptom control. |
| 01049 | Did not meet exclusion criteria number 10: Patients with brain metastases with symptoms or less than 14 days of symptom control. |
| 08001 | Did not meet inclusion criteria number 6: Recovered from damages (≤grade 1) associated with other treatments per the CTCAE version 5.0. |
| 08002 | Did not meet inclusion criteria number 3: Patients with Epidermal Growth Factor Receptor mutation-positive (EGFR+) NSCLC with failure of prior EGFR tyrosine kinase inhibitors (TKIs), or T790M mutation with failure of prior 3rd-generation EGFR TKIs. |
| 09007 | Did not meet inclusion criteria number 9: Be willing and able to provide written informed consent for the trial, and comply with all aspects of the protocol. |
| 09009 | Did not meet inclusion criteria number 7: Adequate function of the important organs. |
| 10001 | Did not meet exclusion criteria number 10: Patients with brain metastases with symptoms or less than 14 days of symptom control. |

Table S2. Baseline characteristics of patients in the phase I part

|  | **Total (n=9)** |
| --- | --- |
| **Age-years, median (range)** | 64 (44-66) |
| **Male, n (%)** | 4 (44.4) |
| **ECOG PS, n (%)** |  |
| 1 | 9 (100.0) |
| **Smoking status, n (%)** |  |
| Never | 7 (77.8) |
| Former or current | 2 (22.2) |
| **Disease stage, n (%)** |  |
| IV | 9 (100.0) |
| **EGFR mutation, n (%)** |  |
| Exon 19 deletion | 3 (33.3) |
| Exon 21 Leu858Arg | 6 (66.7) |
| **T790M mutation, n (%)** | 2 (22.2) |
| **The number of prior TKI therapy lines, n (%)** |  |
| 1 | 7 (77.8) |
| 2 | 2 (22.2) |
| **Brain metastases, n (%)** | 2 (22.2) |

ECOG PS, Eastern Cooperative Oncology Group Performance Status; EGFR, epidermal growth factor receptor; TKI, tyrosine kinase inhibitors.

Table S3. Subsequent treatment information

| **Subsequent treatment, n (%)** | **Total (n = 40^†^)** |
| --- | --- |
| EGFR-TKI (monotherapy, + chemotherapy, + chemotherapy + bevacizumab, + bevacizumab) | 26 (65.0) |
| Bevacizumab + chemotherapy | 15 (37.5) |
| PD-1 + bevacizumab + chemotherapy | 9 (22.5) |
| PD-1 + chemotherapy /PD-1 + anlotinib /PD-1 + bevacizumab /PD-1 + ADC | 14 (35.0) |
| Chemotherapy | 4 (10.0) |
| Bispecific antibody | 3 (7.5) |

**^†^** Five patients remained on treatment, three discontinued without receiving subsequent treatment, four died, and three were lost to follow-up.

EGFR, epidermal growth factor receptor; TKI, tyrosine kinase inhibitors; PD-1, programmed death 1; ADC, antibody-drug conjugates.

Table S4. Toxicities leading to dose reductions of anlotinib

| **Toxicities** | **Grade** |
| --- | --- |
| **Hematologic toxicities** | |
| Platelet count decreased | Grade 3 |
| Neutrophil count decreased | Grade 3-4 |
| **Non-hematological toxicities** | |
| Abnormal liver function (elevated ALT, AST, or TBIL) | Grade 3-4 |
| Proteinuria | Grade 3 |
| Hemorrhage (including hemoptysis, gastrointestinal bleeding, epistaxis, bronchial bleeding, gum bleeding, gross hematuria, fecal occult blood, cerebral hemorrhage, etc.) | Grade 2 |
| Hypertension | Grade 3 |
| Hand-foot skin reaction | Grade 3-4 |
| Others | Grade 2-3 |
| Abbreviation: ALT, alanine aminotransferase; AST, aspartate aminotransferase; TBIL, total bilirubin.  AEs were graded per the National Cancer Institute Common Terminology Criteria for Adverse Events (NCI CTCAE) version 5.0. | |

Table S5. Modifications for infusion reactions

| **Grade** | **Modifications** |
| --- | --- |
| Grade 1 | 50% reduction in the initial infusion rate; closely monitor for any worsening symptoms, with clinical intervention if necessary |
| Grade 2 | Suspend the benmelstobart injection until the infusion reaction is relieved to grade 0-1; 50% reduction in the initial infusion rate; closely monitor for any worsening symptoms with clinical intervention according to local medical practice |
| Grade 3 | Benmelstobart was immediately and permanently discontinued, and appropriate therapeutic interventions were implemented according to local medical practice |
| AEs were graded per the National Cancer Institute Common Terminology Criteria for Adverse Events (NCI CTCAE) version 5.0. | |

Table S6. Modifications for immune-related adverse events

| **irAEs** | **Grade** | **Modifications** |
| --- | --- | --- |
| Respiratory diseases | Grade 2 | Suspend the benmelstobart injection and use glucocorticoids for symptomatic management until toxicity is relieved to grade 1 or below. If toxicity does not recover within 12 weeks after the last dose, treatment will be discontinued |
|  | Grade ≥3 or recurrent | Discontinuation of treatment |
| Hepatitis | Grade 2 (ALT >3 × ULN or AST <5 × ULN, or TBIL >1.5 × ULN, TBIL <3 × ULN) and lasting for more than 5 days | Suspend the benmelstobart injection and use glucocorticoids for symptomatic management until toxicity is relieved to grade 1 or below. If toxicity does not recover within 12 weeks after the last dose, treatment will be discontinued |
|  | Grade ≥3 (ALT ≥5 × ULN or AST ≥5 × ULN, or TBIL ≥3 × ULN) | Discontinuation of treatment |
| Colitis/diarrhea | Grade 2 or 3 | Suspend the benmelstobart injection and use glucocorticoids for symptomatic management until toxicity is relieved to grade 1 or below. If toxicity does not recover within 12 weeks after the last dose, treatment will be discontinued |
|  | Grade 4 | Discontinuation of treatment |
| Pancreatitis | Grade 2 or 3 pancreatitis; grade 2 or 3 amylase or lipase elevated | Suspend the benmelstobart injection and use glucocorticoids for symptomatic management until toxicity is relieved to grade 1 or below. If toxicity does not recover within 12 weeks after the last dose, treatment will be discontinued |
|  | Grade ≥4 or recurrent pancreatitis | Discontinuation of treatment |
| Endocrine dysfunction | Grade 2 or 3 hypophysitis | Suspend the benmelstobart injection and use glucocorticoids for symptomatic management until toxicity is relieved to grade 1 or below. If toxicity does not recover within 12 weeks after the last dose, treatment will be discontinued |
|  | Grade 4 hypophysitis | Discontinuation of treatment |
|  | Grade ≥3 hyperglycemia (fasting glucose >13.9-27.8 mmol/L) | Suspend the benmelstobart injection and use insulin until the blood sugar levels back to the 2nd level and lower and maintain stability |
|  | Symptomatic hypothyroidism and hyperthyroidism, or asymptomatic hyperthyroidism (TSH <0.1 mIU/L) | Suspend the benmelstobart injection and use the relevant symptomatic treatment until the relevant indicators return to the inclusion criteria |
|  | Grade ≥2 | Suspend the benmelstobart injection and use glucocorticoids for symptomatic management until toxicity is relieved to grade 1 or below. If toxicity does not recover within 12 weeks after the last dose, treatment will be discontinued |
| Encephalitis/meningitis | All grades | Discontinuation of treatment |
| Motor and nervous system diseases | Grade 2 | Suspend the benmelstobart injection and use the appropriate drug until toxicity is relieved to grade 1 or below. If toxicity does not recover within 12 weeks after the last dose, treatment will be discontinued |
|  | Grade ≥3 or recurrent | Discontinuation of treatment |
|  | All grades Guillain–Barré syndrome | Discontinuation of treatment |
|  | All grades of myasthenia gravis | Discontinuation of treatment |
| Skin and subcutaneous tissue disorders | Grade 2 or 3 | Suspend the benmelstobart injection and use the appropriate drug until toxicity is relieved to grade 1 or below. If toxicity does not recover within 12 weeks after the last dose, treatment will be discontinued |
|  | Grade 4 | Discontinuation of treatment |
| Eye diseases | Grade 2 | Suspend the benmelstobart injection and use the appropriate drug until toxicity is relieved to grade 1 or below. If toxicity does not recover within 12 weeks after the last dose, treatment will be discontinued |
|  | Grade ≥3 or recurrent | Discontinuation of treatment |
| Others | Grade 2 or 3 | Suspend the benmelstobart injection and use the appropriate drug until toxicity is relieved to grade 1 or below. If toxicity does not recover within 12 weeks after the last dose, treatment will be discontinued |
|  | Grade 4 | Discontinuation of treatment |
| Abbreviation: irAE, immune-related adverse event; ULN, the upper limit of normal; ALT, alanine aminotransferase; AST, aspartate aminotransferase; TBIL, total bilirubin; TSH, thyroid Stimulating Hormone.  AEs were graded per the National Cancer Institute Common Terminology Criteria for Adverse Events (NCI CTCAE) version 5.0. | | |
