# Supplementary material for: Benmelstobart plus anlotinib in patients with EGFR-positive advanced NSCLC after failure of EGFR TKIs therapy: a phase I/II study
Source: Signal Transduct Target Ther. 2024 Oct 10;9:283. doi: 10.1038/s41392-024-01982-2 (PMC11467201; doi:10.1038/s41392-024-01982-2)
Supplement: Supplementary file 2 — Clinical Study Protocol [file 41392_2024_1982_MOESM2_ESM.docx]

**TQB2450 plus anlotinib in EGFR+ advanced non-small-cell lung cancer patients failed to prior EGFR TKI therapies: a multi-center, single-arm, phase I/II study**

**Clinical Study Protocol**

**Version Number:** 2.0

**Version Date:** November 18, 2019

**Principal Investigator:** Professor, Meiqi Shi

**Unit of Data Management:** Jiangsu Cancer Hospital

**Sponsor:** Chia Tai Tianqing Pharmaceutical Group Co., Ltd.

**Contents**

[**Protocol Synopsis** 1](#_Toc177200810)

[**Study Procedures** 6](#_Toc177200811)

[**List of Abbreviations** 11](#_Toc177200812)

[1. Background 14](#_Toc177200813)

[1.1 Feasibility of study protocol 14](#_Toc177200814)

[1.2 Overview of TQB2450 15](#_Toc177200815)

[2. Study objective 20](#_Toc177200816)

[3. Study design 20](#_Toc177200817)

[3.1 Overall design 20](#_Toc177200818)

[3.2 Type of comparison 21](#_Toc177200819)

[3.3 Sample size calculation 21](#_Toc177200820)

[4. Subjects 21](#_Toc177200821)

[4.1 Diagnostic criteria 21](#_Toc177200822)

[4.2 Inclusion criteria 21](#_Toc177200823)

[4.3 Exclusion criteria 22](#_Toc177200824)

[4.4 Screening failure 23](#_Toc177200825)

[4.5 Withdrawal criteria 24](#_Toc177200826)

[**5.** **Study drugs** 25](#_Toc177200827)

[**5.1** **Basic information about the study drugs** 25](#_Toc177200828)

[**5.2** **Packaging of the study drugs** 25](#_Toc177200829)

[**5.3** **Management of the study drugs** 26](#_Toc177200830)

[**5.4** **TQB2450preparation method** 26](#_Toc177200831)

[**5.5** **Administration regimens of the study drugs** 26](#_Toc177200832)

[**5.6** **Concomitant medications** 32](#_Toc177200833)

[6. Study procedures and assessments 34](#_Toc177200834)

[6.1 Study procedures 34](#_Toc177200835)

[6.2 Study assessments 42](#_Toc177200836)

[6.3 Maximum tolerated dose and dose-limiting toxicity 43](#_Toc177200837)

[6.4 Efficacy assessment 44](#_Toc177200838)

[6.5 Safety assessment 45](#_Toc177200839)

[**7.** **Data management and statistical analysis** 49](#_Toc177200840)

[**7.1** **Data entry** 49](#_Toc177200841)

[**7.2** **Data verification** 49](#_Toc177200842)

[**7.3** **Data review and cleaning** 50](#_Toc177200843)

[**7.4** **Electronic signature** 50](#_Toc177200844)

[**7.5** **Database locking** 50](#_Toc177200845)

[**7.6** **Data transfer** 50](#_Toc177200846)

[**7.7** **Data preservation** 50](#_Toc177200847)

[**8.** **Statistical analysis management** 50](#_Toc177200848)

[**8.1** **Analysis datasets** 50](#_Toc177200849)

[**8.2** **Statistical Analysis Plans** 51](#_Toc177200850)

[**9.** **Project management** 53](#_Toc177200851)

[**9.1** **Information promulgation** 53](#_Toc177200852)

[**9.2** **GCP and ethics committee review and approval** 53](#_Toc177200853)

[**9.3** **Informed consent** 53](#_Toc177200854)

[**9.4** **Protocol modification** 54](#_Toc177200855)

[**9.5** **Protocol deviation** 54](#_Toc177200856)

[**9.6** **Retention of document** 54](#_Toc177200857)

[**9.7** **Study discontinuation** 54](#_Toc177200858)

[**9.8** **End of study** 54](#_Toc177200859)

[**9.9** **Quality control and quality assurance** 55](#_Toc177200860)

[**9.10** **Publication policy** 55](#_Toc177200861)

[**10.** **Participanting center and personnel.** 55](#_Toc177200862)

[**10.1** **Participanting center, center number, and principal investigator (sorted by center number)** 55](#_Toc177200863)

[**10.2** **Lead center and principal investigator** 55](#_Toc177200864)

[**10.3** **Statistical analysis unit and responsible person** 55](#_Toc177200865)

[**10.4** **Co-organizer and principal** 56](#_Toc177200866)

[**10.5** **Research progress** 56](#_Toc177200867)

**Protocol Synopsis**

| Protocol Title | TQB2450 plus anlotinib in EGFR+ advanced non-small-cell lung cancer patients failed to prior EGFR TKI therapies: a multi-center, single-arm, phase I/II study |
| --- | --- |
| Protocol Number | ALTER-L038 |
| Sponsor | Jiangsu Cancer Hospital |
| Co-sponsor | Chia-tai Tianqing Pharmaceutical Co., Ltd. |
| Study Nature | A phase I/II study |
| Study Population | Patients with EGFR+ advanced non-small-cell lung cancer who failed prior EGFR TKI therapies |
| Objective | Phase I part: To determine the maximum tolerable dose (MTD) of anlotinib and TQB2450.  Phase II part: To evaluate the efficacy and safety in EGFR+ advanced non-small-cell lung cancer patients who failed prior EGFR TKI therapies |
| Study Design | Phase I part: 3+3 dose-escalation study  Patients are treated with anlotinib at 3 levels of 8 mg, 10 mg, and 12mg, and TQB2450 at a fixed dose of 1200 mg. The dose-escalation starts with the combination of 8mg anlotinib and TQB2450. Monitor dose-limiting toxicity (DLT) during one cycle to determine the MTD for patients. MTD is defined as the maximum dose with ≤33% risk of DLT. DLT is defined as drug-related toxicity occurring during the first 21 days of therapy that results in grade 4 hematological toxicity, neutropenia (ANC<1000/μL, fever of ≥38.5℃), grade 2 hepatic or renal dysfunction (lasting >7 days despite symptomatic treatment), and grade 3 or higher non-hematological toxicity (excluding alopecia).  Phase II part: A multi-center, single-arm, phase II study  The eligible patients are treated with anlotinib at the dose of 12 mg plus TQB2450 at the dose of 1200 mg, which is determined in phase I study. All patients were treated until events meeting the termination criteria. The efficacy and safety of anlotinib combined with TQB2450 are further evaluated. |
| Planned Sample Size | Phase I part: 3-18 patients  Phase II part: 54 patients |
| Principal Investigator | Professor Meiqi Shi |
| Clinical Study Unit | Jiangsu Cancer Hospital |
| Screening Criteria | **Inclusion criteria**  The inclusion criteria were:   1. Male or female, aged 18-75 years; 2. Histologically and cytologically confirmed locally advanced/advanced non-small cell lung cancer (NSCLC), assessed as stage IIIB-Ⅳ NSCLC (according to the 8th edition of the tumor–node–metastasis staging system of the American Joint Committee on Cancer), or NSCLC patients with postoperative recurrence (all types); 3. Patients with Epidermal Growth Factor Receptor mutation-positive (EGFR+) NSCLC with failure of prior EGFR tyrosine kinase inhibitors (TKIs), or T790M mutation with failure of prior 3^rd^-generation EGFR TKIs; 4. EGFR TKI should be the mainstay of the initial treatment for patients. Patients are allowed to receive ≤1 systemic chemotherapy; 5. Patients with previous only 1^st^/2^nd^-generation EGFR TKIs were required to provide genetic testing reports showing no primary or acquired T790M mutation; patients with previous 3^rd^-generation EGFR TKIs were required to provide genetic testing reports showing EGFR mutation was positive;   Treatment failure is defined as (1) the progressive disease (PD) assessed by imaging or clinical evidence during or after EGFR TKIs treatment, or withdrawal from standard treatment for intolerant adverse events (AEs). The intolerant AEs included grade Ⅳ hematologic toxicity, or grade ≥Ⅲ nonhematologic toxicity, or grade ≥Ⅱ main organ damage such as heart, liver, and kidneys per Common Terminology Criteria for Adverse Events (CTCAE) version 5.0.  Detection results from other hospitals were also acceptable. Tissue is the first choice for detection samples, if optional blood samples cannot be obtained from tissue, the detection methods can be selected by traditional genetic testing (such as immunohistochemistry, ddPCR, Arms-PCR, etc.) or high-throughput sequencing.   1. At least one measurable lesion according to Response Evaluation Criteria in Solid Tumors (RECIST) version 1.1, without prior radiotherapy in the past three months; 2. Predicted life expectancy of ≥3 months; Eastern Cooperative Oncology Group (ECOG) performance status (PS) of 0-1; 3. Recovered from damages (≤grade 1) associated with other treatments per the CTCAE version 5.0; 4. Adequate function of the important organs as evident by the following criteria: 5. Hemanalysis (no blood transfusion, and without the use of G-CSF or other hematopoietic stimulating factors within 14 days): 6. Hemoglobin (HB) ≥80g/L; 7. Absolute neutrophil count (ANC) ≥1.5×10^9/L; 8. Platelets (PLT) ≥75×10^9/L; 9. Biochemistry: 10. Total bilirubin (TBIL) ≤upper limit of normal value (ULN); 11. Alanine aminotransferase (ALT) and aspartate aminotransferase (AST) ≤1.5×ULN; 12. Creatinine (Cr) ≤1.5×ULN or creatinine clearance rate (CCr) ≥60 mL/min; 13. Urinary protein <++; 14. Coagulation function: INR and APTT ≤1.5×ULN; 15. Doppler ultrasound assessment: left ventricular ejection fraction (LVEF) ≥50%; 16. Women of childbearing potential must have adopted contraception, or have a negative serum and urine pregnancy test within 7 days before the study enrollment; women of childbearing potential and men must agree to contraception for the duration of study treatment and 8 weeks after the last dose of study treatment, or have been of sterilization; 17. Be willing and able to provide written informed consent for the trial, and comply with all aspects of the protocol.   **Exclusion criteria**  The exclusion criteria were:   1. Patients with a history of malignant tumors within 5 years before the start of treatment except for patients with cured orthotopic cervical cancer, cutaneous basal cell carcinoma, cutaneous squamous cell carcinoma, or superficial bladder cancer (Ta [non-invasive cancer], Tis [orthotopic cancer], T1 [superficially invasive]); 2. Central squamous cell lung cancer or squamous cell lung cancer with a significant pulmonary cavity; 3. Have received >1 systemic chemotherapy during previous lines of treatment (patients with neoadjuvant or adjuvant chemotherapy were allowed to be enrolled); 4. Have previously received other anti-PD-1/PD-L1 drugs or immunotherapies targeted PD-1/PD-L1; 5. Patients with severe hypersensitivity reaction after administration of other monoclonal antibodies; 6. Patients with anlotinib allergy; 7. Patients with active autoimmune disease or a history of autoimmune disease (such as, but not limited to, autoimmune hepatitis, interstitial pneumonia, enteritis, vasculitis, and nephritis; patients with asthma that needed bronchodilators for medical intervention were unable to be enrolled); patients with vitiligo, psoriasis, alopecia, and well-controlled type 1 diabetes mellitus who did not require systemic treatment were allowed to be enrolled; 8. Patients who had clinically significant thyroid dysfunction within 6 months before enrollment and whose thyroid function did not return to normal or was clinically insignificant despite medical therapy; 9. Factors that have a significant impact on oral drug absorption, such as inability to swallow, chronic diarrhea, and intestinal obstruction; 10. Patients with brain metastases with symptoms or less than 14 days of symptom control; 11. Severe comorbidities before enrollment: 12. Unstable angina and/or congestive heart failure or vascular disease within 12 months requiring hospitalization (such as aortic aneurysm requiring surgical repair or peripheral venous thrombosis), or other heart damages judged by investigators that could influence the safety assessment of study drugs (such as uncontrolled arrhythmia, myocardial infarction, or ischemia); 13. Esophagogastric varices, unhealed ulcers, uncured wounds, or fracture within 6 months; 14. History of abdominal fistula, gastrointestinal perforation, intra-abdominal abscess, or gastrointestinal bleeding within 6 months; 15. Arterial thromboembolism, grade ≥3 venous thromboembolism, transient ischemic attack (TIA), cerebral vascular accident (CVA), hypertensive crisis, or hypertensive encephalopathy within 6 months; 16. Aggravating chronic obstructive pulmonary disease (COPD) or other respiratory illness requiring hospitalization within 28 days; 17. An active lung infection and/or acute bacterial or fungal infection requiring intravenous antibiotic treatment within 28 days; 18. Clinical jaundice caused by abnormal liver function within 7 days; 19. Hypertension that was uncontrollable with a single antihypertensive drug at present (systolic blood pressure ≥160 mmHg or diastolic blood pressure ≥100 mmHg); or being treated with a combination of two or more antihypertensive drugs; 20. Major surgical operation, biopsy, or obvious traumatic injury within 28 days before enrollment; 21. Virologic testing during screening shows any of the following: 22. HBsAg positive, and HBV DNA ≥1×10^3^ copies/mL; 23. Anti-HCV positive and HCV >ULN; 24. HIV-positive; 25. Immune checkpoint inhibitors or systemic therapies were required to achieve immunosuppression (prednisone or other hormones >10 mg/d); 26. Patients with other anti-cancer therapies or participated in other clinical studies (not EGFR TKI) within 4 weeks before the first dose of the drug; patients who received therapies or participated in other clinical studies of EGFR TKI within 2 weeks before the first dose of the drug; 27. Imaging showed that the tumor had invaded the important blood vessels, or the tumor was likely to invade the important blood vessels during the subsequent study and cause massive hemorrhage judged by investigators; 28. Patients with any bleeding signs or a history of bleeding, regardless of severity, having grade ≥3 bleeding events per CTCAE, unhealed wounds, anabiosis, or fracture within 4 weeks before grouping; 29. Clinically hemoptysis (daily hemoptysis greater than 50ml) within 2 months before enrollment (defined as coughing up ≥1 teaspoon of blood, small blood clots, or coughing up blood without sputum); but patients with blood in sputum were not excluded; 30. A history of psychotropic substance abuse and cannot be abstinent, or patients with mental disturbance; 31. Have previously received antiangiogenic drugs; 32. Conditions that increased the risk associated with study participation or the study medication, and could make a patient ineligible for study in the investigator's judgment. |
| Dropout/Removal Criteria | - 1. Participants who do not meet the screening criteria;   2. Participants who are unable to evaluate for efficacy or unable to evaluate safety without receiving 1 dose of the drug;   3. Participants who are treated with chemotherapy, surgical or experimental drugs other than this protocol during the trial;   4. Participant's concurrent use of China Food and Drug Administration (CFDA)-approved modern herbal agents and immunomodulatory agents for the treatment of tumors;   5. Participants who do not follow the dose, method, and course of treatment according to the study protocol. |
| Termination Criteria | Subject must termination the study if the following cases occur (including but certainly not limited to):   - 1. Disease progression (PD) determined according to RECIST 1.1 criteria or considered clinically progressive.   2. Termination of treatment decided by the investigator from the patient's best interests.   3. Occurrence of intolerable adverse reactions or severe adverse events confirmed by the investigator.   4. Patients with poor compliance, using drug amounts outside the range of 80% to 120% of the prescribed dose.   5. Patient withdrawal of informed consent.   6. Initiation of other anti-tumor drug treatments affecting efficacy judgment (such as chemotherapy, targeted therapy, or biologic agents).   7. Unexpected pregnancy. |
| Study Schedule | Estimated date of the start of the study: August 2019  Estimated enrollment date of the last subject: August 2020  Estimated date of the end of the study: August 2021 |
| Study Procedures | TQB2450 plus anlotinib hydrochloride capsules were administered in a 21-day cycle until disease progression or intolerance. |
| Primary effect endpoints | Duration of response (DoR); overall survival (OS); 12-month progression-free survival rate; disease control rate (DCR); objective response rate (ORR) (assessed per RECIST version 1.1 at the following time points: 3 weeks after the first therapy, then every 6 weeks thereafter during therapy.) |
| Primary safety endpoints | Dose-limiting toxicities (DLT); maximum tolerable dose (MTD); vital signs; laboratory index; adverse event (AE); immune-related adverse event (irAE); serious adverse event (SAE) according to NCI CTCAE version 5.0. |
| Sample Size calculation | Phase I part: Following the 3+3 dose-escalation design, the study anticipates a minimum of 3 and a maximum of 18 participants to be enrolled.  Phase II part: Based on prior efficacy data achieved with carboplatin plus pemetrexed in EGFR+ NSCLC patients who failed to EGFR TKI, an mPFS of 5 months was utilized as a historical control to determine the sample size for the phase II part. We hypothesized that TQB2450 plus anlotinib would lead to clinical benefit in this population, with a mPFS of 9 months. Sample sizes of 54 in the study achieved 90% power at a significance level (α) of 0.05 with an anticipated dropout rate of 20%. The study is expected to last for 24 months, including a 12-month patient enrollment period. At the study's conclusion, an estimated 32 endpoint events are expected to occur. |
| Statistical Analysis | The PFS, median OS, and DoR and associated two-sided 95% confidence intervals (CIs) were constructed based on the Kaplan-Meier methods. The disease control rate (DCR = CR + PR + SD), objective response rate (ORR = CR + PR), 12-month survival rate, and 12-month PFS rate are expressed as percentages.  Safety analysis is summarized descriptively, detailing AE and irAE observed in this trial in a form. |

**Study Procedures**

| **Visit** | **Screening[1]** | | **Treatment period** | | | | **Post-treatment** | | | **Follow-up of survival** | | |
| --- | --- | --- | --- | --- | --- | --- | --- | --- | --- | --- | --- | --- |
|  |  |  |  |  |  |  | **Withdrawal** | **End-of-**  **treatment** | **Before progression[2]** | | **After progression** | **After DCO[3]** |
|  | 1 | 2 | 3 | 4 | 5+ | 6+[4] | NA | NA | NA | | NA | NA |
| **Visit windows** | -28-0 | -7-0 | ±3 | ±3 | ±3 | ±3 | ±3 | ±3 | ±7 | | ±7 | ±7 |
| **Treatment cycles** | NA | NA | C1D7 | C1D21 | C3D21 | C5D21+ | NA | NA | NA | | NA | NA |
| **Study weeks** | -4-0 | -1-0 | 1 | 3 | 9 | 15+ | NA | With 21 days after treatment discontinuation | Every 6 weeks | | Every 6 weeks | Every 8 weeks |
| **Baseline characteristics** | | | | | | | | | | | | |
| **Informed consent** | X |  |  |  |  |  |  |  |  | |  |  |
| **Demographics** | X |  |  |  |  |  |  |  |  | |  |  |
| **Inclusion/exclusion criteria** | X |  |  |  |  |  |  |  |  | |  |  |
| **Enrollment ID** |  | X |  |  |  |  |  |  |  | |  |  |
| **Physical examination [5]** |  | X | X | X | X | X | X |  |  | |  |  |
| **Vital signs [6]** |  | X | X | X | X | X | X |  |  | |  |  |
| **Medical history [7]** | X |  |  |  |  |  |  |  |  | |  |  |
| **Compliance before enrollment [8]** |  | X |  |  |  |  |  |  |  | |  |  |
| **Radiographic evidence of progression** | X |  |  |  |  |  |  |  |  | |  |  |
| **Laboratory evaluations** | | | | | | | | | | | | |
| **Hemanalysis [9]** |  | X | X | X | X | X | X | X |  | |  |  |
| **Urinalysis [10]** |  | X | X | X | X | X | X | X |  | |  |  |
| **Stool tests [11]** |  | X | X | X | X | X | X |  |  | |  |  |
| **Blood biochemistry [12]** |  | X | X | X | X | X | X | X |  | |  |  |
| **Coagulation function [13]** |  | X | X | X | X | X | X |  |  | |  |  |
| **Thyroid function [14]** |  | X | X | X | X | X | X |  |  | |  |  |
| **Tumor marker** |  | X | X | X | X | X | X |  |  | |  |  |
| **12-ECG [15]** |  | X | X | X | X | X | X |  |  | |  |  |
| **Myo-cardial enzymonram [16]** |  | X |  |  |  |  |  |  |  | |  |  |
| **Echocardiography [17]** |  | X |  |  |  |  | X |  |  | |  |  |
| **Hepatitis B, Hepatitis C, HIV [18]** | X |  | | | | | | | | | | |
| **Pregnancy test [19]** |  | X |  |  |  |  | X |  |  | |  |  |
| **Imaging assessment** | | | | | | | | | | | | |
| **Imaging assessment [20]** |  | X |  | X | At the end of every two cycles (6 weeks ±7 days) | | X |  | X | |  |  |
| **Examination of brain lesions [21]** |  | X |  | X | At the end of cycle 3 (±7 days) and every 4 weeks thereafter (12 weeks ±7 days) | | X |  | X | |  |  |
| **Other clinical assessments** | | | | | | | | | | | | |
| **ECOGPS** |  | X |  | X | X | X | X | X |  | |  |  |
| **Neurological function evaluation and corticosteroid use** |  | X |  | X | At the end of cycle 3 (±7 days) and every 4 weeks thereafter (12 weeks ±7 days) | | X |  | X | |  |  |
| **Blood pressure monitoring [22]** | X | X | X | X | X | X | X |  |  | |  |  |
| **Adverse events [23]** |  |  | X | X | X | X | X | X |  | |  |  |
| **PD-L1 expression [24]** | X |  |  |  |  |  |  |  |  | |  |  |
| Study drugs | | | | | | | | | | | | |
| **TQB2450** |  | | 3-week cycle, given on day 1 in a cycle | | | |  |  |  | |  |  |
| **Anlotinib** |  |  | 3-week cycle (2 weeks on and 1 week off) | | | |  |  |  | |  |  |
| **Distribution/recycling of anlotinib** |  | X |  | X | X | X | X |  |  | |  |  |
| **Concomitant treatments [25]** | X | X | X | X | X | X | X | X | X | | X | X |
| **Drug compliance [26]** |  |  | X | X | X | X | X |  |  | |  |  |
| **Follow-up of survival [27]** | | | | | | | | | | | | |
| **Time to disease progression [28]** |  |  |  |  |  |  | every 6 weeks (±3 days) until disease progression or initiating other anti-tumor therapy (non-imaging PD patients) | | | |  |  |
| **Time to death** |  |  |  |  |  |  |  |  |  | | X | X |
| **Tomur treatment [29]** |  |  |  |  |  |  |  | X | X | | X | X |

Note: All observation indicators and examination schedule will not be affected by duration of treatment discontinuation, but occasional changes in each wisit window due to holidays or other administrative reasons are allowed.

The window period of 3 days for screening indicators and examination must be completed before the initiation of the treatment cycle, for example, screening should not be repeated if performed within 7 days before the initiation of the first cycle of treatment. NA: not applicable.

1. Subjects must complete the screening visit within 28 days after imaging progression;
2. Subjects who withdraw from the study without objective progress will continue to be followed up until the date of objective progress occurred or data cut-off;
3. DCO: Data cut-off;
4. Subjects are visited every 6 weeks from V5;
5. Physical examination: Whole body system examination (facial features, integumentary system, lymph nodes, eyes, ears, nose, throat, abdomen, spine, extremities, and nervous system) are performed within 7 days before enrollment, at C1D7, C1D21, and thereafter at days 21 of every two cycles (odd numbered cycles), and at dropout visit;
6. Vital signs: Temperature, pulse rate, and respiratory rate are performed within 7 days before enrollment, at C1D7, C1D21, and thereafter at days 21 of every two cycles (odd numbered cycles), and at dropout visit; Blood pressure monitoring is described in [22];
7. Medical history: Pathological results, EGFR T790M mutation test report; history of tumor surgery, chemotherapy, radiotherapy, and other disease treatment;
8. Compliance before enrollment: Days between the first progression of the subject and enrollment;
9. Hemanalysis: Hemoglobin, red blood cells, white blood cells, neutrophil count, lymphocyte count, and platelets count are performed within 7 days before enrollment, at C1D7, C1D21, and thereafter at days 21 of every two cycles (odd numbered cycles), and at dropout visit, and at days 21 after treatment. If the neutrophil count ≤1×109/L or platelets (PLT) ≤50×109/L, additional examination (1 time/2-3 days) is required; weekly hemanalysis is required if there is a delay in dosing or dose adjustment due to hematological toxicity;
10. Urinalysis: Urine protein, glucose, occult blood (red blood cell and white blood cell), pH, and ketone bodies are performed within 7 days before enrollment, at C1D7, C1D21, and thereafter at days 21 of every two cycles (odd numbered cycles), and at dropout visit, and at days 21 after treatment; If the semi-quantitative method shows the levels of protein ≥2+ (e.g., urine test strip), 24-hour urine protein quantification should be performed;
11. Stool tests: Occult blood test is performed within 7 days before enrollment, at C1D7, C1D21, and thereafter at days 21 of every two cycles (odd numbered cycles), and at dropout visit.
12. Blood biochemistry: Liver function (TP, A, G, ALT, AST, LDH, ALP, TBil, DBil, and IBil), renal function (BUN, Cr, and UA), blood lipid tetrachoric (TC, TG, HDL, and LDL), electrolytes (K+, Na+, CL-, Ca2+, Mg2+, and P), lipase, amylase, fasting glucose, etc. are performed within 7 days before enrollment, at C1D7, C1D21, and thereafter at days 21 of every two cycles (odd numbered cycles), and at dropout visit, and at days 21 after treatment; During the drug administration, patients who develop early symptoms of liver injury (e.g., appetite loss, vomiting, right upper abdominal discomfort, malaise, etc.) before blood biochemical abnormalities should undergo blood biochemistry immediately. If ALT or AST is up to 3 times ULN or baseline value and total bilirubin up to 2 times the ULN or baseline value, the frequency of tests should be increased (recommended 1-2 times/week);
13. Coagulation function (PT, APTT, TT, Fbg, and INR) are performed within 7 days before enrollment, at C1D7, C1D21, and thereafter at days 21 of every two cycles (odd numbered cycles), and at dropout visit.
14. Thyroid function (T3, T4, FT3, FT4, and TSH) are performed within 7 days before enrollment, at C1D7, C1D21, and thereafter at days 21 of every two cycles (odd numbered cycles), and at dropout visit.
15. 12-ECG are performed within 7 days before enrollment, at C1D7, C1D21, and thereafter at days 21 of every two cycles (odd numbered cycles), and at dropout visit. If an abnormal ECG is identified, two additional confirmations must be performed (each 5 minutes apart and the QTc interval should be indicated);
16. Myo-cardial enzymonram is detected within 7 days before enrollment. This examination is supplemented only when symptoms such as precordial pain, palpitations, and ECG abnormalities are present;
17. Echocardiography is performed within 7 days before enrollment and at dropout visit. This examination is supplemented only when clinically meaningful ECG abnormalities occur during treatment;
18. Hepatitis B virus (HBV) and hepatitis C virus examination: HBV examination (HBsAg, HBsAb, HBeAg, HBeAb, and HBcAb) is performed and if the test result is abnormal, viral replication (HBA DNA) and hepatitis C virus antibody (anti-HCV) test should be conducted;
19. Pregnancy testing is limited to women of childbearing age, who are required to undergo a pregnancy test within 7 days before enrollment;
20. Imaging assessment: Including CT or MRI of the chest, abdomen, pelvis, and head. Baseline assessment of screening period tumors can be extended to within 3 weeks before treatment, and CT/MRI scans obtained before signing informed consent can be used for screening period tumor assessment if they meet the criteria; bone scans are required if suspected bone metastasis; no symptoms of cerebral hemorrhage need to be confirmed within 28 days before treatment in patients with stable brain metastases.

The imaging examination should be performed on chest and abdominal lesions under the same conditions as the baseline (layer thickness of the scan, use of contrast agents, etc.) on C1D7 and days 21 of every odd numbered cycle; Other lesions identified at baseline or new lesions suspected subsequently should also be examined at the appropriate time. Subjects should be promptly imaged when outgroup for any reason;

The window of the imaging schedule is ±7 days. An unplanned imaging examination may be performed when disease progression (e.g., symptoms worsening) is suspected. Subjects who discontinue treatment for reasons other than imaging-confirmed disease progression should perform an image evaluation at the date of end-of-treatment, and tumor efficacy evaluations are performed every 8-week follow-up thereafter until confirmed disease progression or initiating new tumor treatment is initiated.

1. Examination of brain lesions: Brain MRI and neurological function tests [19];
2. Blood pressure is measured by the subject himself/herself and recorded in the patient diary card. Blood pressure is tested at least 3 times per week for the first 2 cycles and followed up each day if the blood pressure is abnormal. In addition, blood pressure is measured again by the investigator at each follow-up visit, coffee, and tobacco intake are prohibited within 30 minutes before each blood pressure is measured. The measurement of blood pressure is taken in the sitting position with the arm at heart level after at least 10 minutes of quiet sitting, and each blood pressure measurement is taken on the same side;
3. AEs are recorded from the first dose of the study drug until at least 21 days after the last dose and follow-up until the AEs resolved or stabilized;
4. PD-L1 expression: Biopsy tissues are obtained from appropriate subjects for PD-L1 expression using immunohistochemistry.
5. Concomitant medications and therapy during the trial should be recorded, and the concomitant medication and therapy are recorded only in the case of novel or unresolved AEs associated with treatment if the subject discontinues treatment;
6. Study drug compliance: Drug doses, counts, and compliance for the previous cycle are calculated and recorded in CRF form at the first day of each cycle;
7. Survival follow-up: after discontinuation of trial treatment, survival status and subsequent antitumor therapy can be collected every 8 weeks by clinical or telephone follow-up until death;
8. Time to disease progression: for patients with non-imaging evidence of progression (intolerable and other conditions), imaging evaluation should continue every 8 weeks until disease progression, initiation of other oncologic therapy, death, or end of the study;
9. Tumor treatment is recorded during the follow-up period.

**List of Abbreviations**

| Abbreviations | Full term |
| --- | --- |
| ACE-I | Angiotensin-Converting Enzyme Inhibitors |
| AE | Adverse Event |
| AKP | Alkaline Phosphatase |
| ALT | Alanine Aminotransferase |
| ANC | Absolute Neutrophil Count |
| APTT | Activated Partial Thromboplastin Time |
| AST | Aspartate Aminotransferase |
| BIL | Bilirubin |
| BUN | Blood Urea Nitrogen |
| BSC | Best Supportive Care |
| Cr | Creatinine |
| CCr | Creatinine Clearance |
| CR | Complete Remission |
| CRF | Case Report Form |
| CT | Computed Tomography |
| CTCAE | Common Terminology Criteria For Adverse Events |
| CFDA | China Food And Drug Administration |
| DCR | Disease Control Rate |
| DLT | Dose-Limiting Dose |
| DRQ | Data Rating Questionnaire |
| ECOG | Eastern Cooperative Oncology Group |
| EGFR | Epidermal Growth Factor Receptor |
| EDC | Electronic Data Capturing |
| ECG | Electro Cardio Gram |
| EORTC | The European Organization For Research And Treatment For Cancer |
| Fbg | Fibrinogen |
| GCP | Good Clinical Practice |
| FAS | Full Analysis Set |
| Glu | Glucose |
| Hb | Hemoglobin |
| HFSR | Hand-Foot Syndrome Reaction |
| HR | Hazard Ratio |
| IDMC | Independent Data Monitoring Committee |
| INR | International Normalized Ratio |
| mPFS | Median Progression Free Survival |
| MRI | Magnetic Resonance Imaging |
| MTD | Maximum Tolerated Dose |
| NSCLC | Non-Small Cell Lung Cancer |
| OB | Occult Blood |
| ORR | Objective Response Rate |
| OS | Overall Survival |
| PD | Progressive Disease |
| PDGFR | Platelet-Derived Growth Factor |
| PFS | Progression-Free Survival |
| PI | Principal Investigator |
| PLT | Platelet |
| PR | Partial Response |
| PRO | Protein |
| PK | Pharmacokinetics |
| PD | Pharmacodynamics |
| PPS | Per Protocol Set |
| PS | Performance Status |
| PT | Prothrombin Time |
| QoL | Quality Of Life |
| RBC | Red Blood Count |
| RECIST | Response Evaluation Criteria In Solid Tumors |
| γ-GT | Gamma Glutamyl Transpeptidase |
| SAE | Serious Adverse Event |
| SAS | Safety Analysis Set |
| SCr | Serum Creatinine |
| SD | Stable Disease |
| TKI | Tyrosine Kinase Inhibitor |
| TT | Thrombin Time |
| TTP | To Tumor Progression |
| ULN | Upper Limit of Normal |
| UA | Uric Acid |
| VEGF | Vascular Endothelial Growth Factor |
| VEGFR | Vascular Endothelial Growth Factor Receptor |
| WBC | White Blood Cell Count |

1. Background
   1. [Feasibility of study protocol](https://www.researchprotocols.org/2018/5/e132)

Lung cancer is the most common malignant tumor globally and the leading cause of cancer-related deaths. In China, lung cancer has the highest incidence and mortality rates among all cancers. In 2015, there were 733,000 new cases of lung cancer and 610,000 deaths reported in China. Of which, non-small cell lung cancer (NSCLC) accounts for approximately 85% of lung cancer. The majority of patients with NSCLC present with locally advanced or metastatic disease at the time of diagnosis, resulting in a very low cure rate.

Non-Small Cell Lung Cancer (NSCLC) standard of care include surgery, radiation therapy, chemotherapy, or a combination of these approaches. Surgery remains the primary treatment for early-stage NSCLC (stage I, II, and selected stage IIIA). Neoadjuvant chemotherapy or preoperative chemotherapy for stages I-IIA NSCLC is currently considered experimental. For locally advanced, unresectable stage III disease, concurrent chemotherapy and thoracic radiation therapy are the current standard of care.

In the first-line treatment of advanced NSCLC, comprehensive molecular testing, such as for epidermal growth factor receptor (*EGFR*), anaplastic lymphoma kinase (*ALK*), proto-oncogene 1, receptor tyrosine kinase (ROS-1), v-raf murine sarcoma viral oncogene homolog B1 (*BRAF*), programmed cell death ligand-1 (PD-L1), etc., can be performed. For patients with positive driver gene mutations, molecular targeted therapies can be applied to intervene at these targets for systemic treatment, significantly extending the overall survival (OS) of NSCLC patients. The *EGFR* mutation is a common activating gene mutation in lung cancer. Tyrosine Kinase Inhibitors (TKIs) competitively bind to the intracellular tyrosine kinase binding site, preventing EGFR from binding to ATP and inhibiting cell proliferation. In the past decade, targeted therapies, with EGFR-TKIs as representatives, have become indispensable for late-stage NSCLC patients with EGFR-sensitive mutations, greatly improving the objective response rate (ORR) and progression-free survival (PFS) for this population. The diagnosis and treatment of lung cancer through molecular targeting have undergone significant changes in the last decade. Testing for the genetic mutation status of advanced lung cancer patients is gradually becoming a routine and crucial basis for personalized treatment. For patients without detected driver gene-sensitive mutations, the treatment plan remains traditional chemotherapy.

However, most patients with *EGFR*-sensitive mutations develop acquired resistance to first-line treatment with first-generation EGFR-TKIs after 10-12 months. Subsequent treatment options have been elucidated by studies such as AURA3 and IMPRESS. The AURA3 study showed that compared to platinum-based doublet chemotherapy, osimertinib significantly prolonged PFS in second-line treatment for *EGFR T790M* mutation-positive patients (10.1 months vs. 4.4 months). The IMPRESS study, comparing the continuation of first-generation TKIs combined with platinum-based doublet chemotherapy to platinum-based doublet chemotherapy in patients with or without the *EGFR T790M* mutation, found that the median PFS for both groups was 5.4 months. The latest OS data from the IMPRESS study, with a maturity of 66%, showed that most deaths were related to disease progression. The OS in the EGFR-TKIs group was inferior to the control group (HR 1.44, P=0.016, median OS 13.4 months vs. 19.5 months). Subgroup analysis results were consistent with the overall conclusion. In *T790M* mutation-positive patients, this OS disadvantage had statistical significance (HR 1.49, P=0.0432), while in T790M mutation-negative patients, there was no statistical difference (HR 1.15). There was no difference in PFS in *T790M* mutation-positive patients between the two groups, and in *T790M* mutation-negative patients, there was a trend but no statistical difference (HR 0.67, P=0.0745). Adverse reactions were mainly nausea and appetite loss, with no observed occurrence of interstitial lung disease. Grade 1-2 gastrointestinal toxicity was more common in the EGFR-TKIs group, and adverse reactions led to the deaths of two patients in the treatment-related group, compared to one death in the control group, also treatment-related. In summary, for patients with EGFR-sensitive mutations who develop resistance to first-line treatment with first-generation EGFR-TKIs, if *T790M* mutation-positive, osimertinib can be chosen; if *T790M* mutation-negative, doublet chemotherapy is recommended. After disease progression following treatment with osimertinib, there is currently no superior solution than chemotherapy.

For patients who develop resistance to first-line treatment with EGFR-TKIs, the feasibility of using vascular-targeted drugs for treatment has been explored through clinical studies. ECOG4599 and AVAIL studies demonstrate that the vascular-targeted drug bevacizumab, when combined with chemotherapy in first-line treatment for advanced NSCLC, can extend OS, achieving significant clinical efficacy in advanced NSCLC. Large molecular antibodies against angiogenesis (Ramucirumab) and small molecule inhibitors (Nintedanib) in combination with docetaxel have shown good clinical outcomes in second-line treatment for advanced lung cancer, significantly extending the survival of lung adenocarcinoma patients. Subgroup analysis results from the ALTER0303 study show that for patients with *EGFR* gene-sensitive mutations or wild-type, anlotinib hydrochloride has significant clinical benefits. In *EGFR*-sensitive mutation patients, the PFS in the anlotinib hydrochloride group (n=93) and the control group (n=45) were 5.6 months and 0.8 months, respectively (p<0.0001), and the OS was 10.7 months and 6.3 months, respectively (p=0.0227). These results suggest the efficacy of anti-angiogenic drugs in treating advanced NSCLC.

The efficacy of immune checkpoint inhibitors (ICIs) in patients with first-line EGFR-TKIs progression remains unclear. In studies such as Checkmate-057 and Keynote-010, nivolumab and pembrolizumab monotherapy, respectively, did not show a survival benefit in patients with EGFR mutation-positive tumors who progressed after first-line EGFR-TKIs treatment. However, it is noteworthy that in the Impower 150 study, the combination of atezolizumab, bevacizumab, paclitaxel, and carboplatin significantly increased the OS of patients with *EGFR* mutation-positive tumors who progressed after first-line EGFR-TKIs treatment, compared to the bevacizumab, paclitaxel, and carboplatin regimen alone. This provides a promising avenue for the use of immune checkpoint inhibitors in patients with EGFR-positive tumors who progress after first-line EGFR-TKIs treatment.

- 1. Overview of TQB2450

TQB2450 is a novel humanized monoclonal antibody against PD-L1. TQB2450 could prevent the binding of PD-L1 to programmed death 1 (PD-1) and B7-1, reactivate the T cells, and enhance the immune response, thus harboring the potential to treat various tumors.

Generic name: TQB2450

English name: TQB2450 Solution for Injection

Pinyin: TQB2450 Zhusheye

Molecular Formula: C6_444_H_9968_N_1692_O_2002_S_46_

Molecular Weight: 144,651 Da (no glycosylation)

- - 1. Overview of pharmacological profile

Tissue cross-reactivity:

The degree of tissue cross-reactivity was assessed by a two-step method for immunohistochemistry. The results revealed that TQB2450 had specific tissue cross-reactivity with 18 normal tissues and 15 tissues of cynomolgus monkeys. The details were as follows:

Table 1.2-1 Tissue cross-reactivity

| Species | Group | Cross-reactive tissue |
| --- | --- | --- |
| Human | TQB2450 | The pituitary gland, brain, cerebellum, lung, liver, skeletal muscle, heart, colon, small intestine, stomach, kidney, bladder, lymph nodes, spleen, thymus, bone marrow, blood cells, and placenta. |
|  | Homotype negative control | Lungs, skeletal muscles, colon, bladder, kidney, stomach, heart, blood cells, and bone marrow. |
| Cynomolgus monkeys | TQB2450 | The pituitary gland, lung, liver, muscle, heart, colon, small intestine, stomach, kidney, bladder, lymph nodes, spleen, thymus, bone marrow, and blood cells. |
|  | Homotype negative control | Kidneys, blood cells, and bone marrow. |

The impact on the cardiovascular system of the macaque monkey:

The cynomolgus monkeys were divided into 4 groups: control group, TQB2450 50 mg/kg group, TQB2450 100 mg/kg group, and TQB2450 200 mg/kg group. Each group had 5 male cynomolgus monkeys and 5 females. Each group was intravenously given control substances (placebo injection without TQB2450) or a corresponding concentration of TQB2450 at the volume of 20 mL/kg. The drugs were delivered once a week for 5 consecutive weeks, the infusion rate was ~2 mL/min. Then, the drug was suspended for 8 weeks. The day of first dose was the first day of the study. Lead II electrocardiogram (heart rate, QRS wave duration, PR interval, RR interval, P-wave travel time, QT interval (QTc), corrected QTc), blood pressure (systolic pressure, diastolic pressure, and mean blood pressure), and respiratory rate were determined at ~0-1, ~5-6, ~24-25, ~48-49, ~96-97 hours before and after the first dose, and at ~0-1 hour after the fifth dose, as well as 1 day before the autopsy following the recovery period.

Results showed that the electrocardiographic parameters, blood pressure, and respiratory rate of female and male monkeys in each group had no obvious abnormal alterations.

- - 1. Overview of toxicology

1.2.2.1 Acute toxicity test

Injection into cynomolgus monkeys:

Six cynomolgus monkeys were divided into 2 groups with 3 (both male and female) in each group. The two groups were given TQB2450 of 200 and 400 mg/kg, respectively, and the two were delivered single intravenous doses (10 mg/mL) of TQB2450 at the volume of 20 or 40 mL/kg, respectively. The day of first dose was the first day of the study.

The general condition of cynomolgus monkeys in each group was observed daily on days 1-14 after administration. Body mass was measured before infusion and on days 4, 9, and 14 of the study. The food intake was measured on days 2~3, 8~9, and 12~13 of the study. Temperature, lead II electrocardiogram, and blood pressure was measured at ~0-1 hour before and after the first dose and on day 14 of the study. Hematology and serum biochemical tests were performed on the 4th and 14th days of the study. All cynomolgus monkeys in each group were euthanized after anesthesia on day 15, and gross anatomy was observed.

Results indicated that general observation, body mass, food intake, temperature, electrocardiogram, blood pressure, hemanalysis, serum biochemistry, urinalysis, and gross anatomy of cynomolgus monkeys showed no obvious abnormalities following single intravenous doses of TQB2450 of 200 and 400 mg/kg. The maximum tolerated dose (MTD) was 400 mg/kg.

1.2.2.2 Long-term toxicity test

Toxicity and toxicokinetics of TQB2450 on cynomolgus monkey following intravenous injection for 4 weeks:

Cynomolgus monkeys were divided into a control group, TQB2450 50 mg/kg group, TQB2450 100 mg/kg group, and TQB2450 200 mg/kg group with 5 males and 5 females in each group. Each group was intravenously given control substances (placebo injection without TQB2450) or a corresponding concentration of TQB2450 at the volume of 20 mL/kg. The drugs were delivered once a week for 5 consecutive weeks, a-nd the infusion rate was ~2 mL/min. Then, the drug was suspended for 8 weeks.

General condition, body mass, food intake, temperature, lead II electrocardiogram, blood pressure, respiratory rate, eye examination, hemanalysis, serum biochemistry, urinalysis, bone marrow examination, complement, circulating immune complex, lymphocyte subpopulation (CD3+, CD3+CD4+, CD3+CD8+, CD3+CD4+/CD3+CD8+, CD3-CD14+, CD3-CD16+, CD20+), hormones (T3, T4, TSH), cytokines (IFN-γ, TNF-α, IL-2, IL-6, IL-10), organ weight and coefficient, gross anatomy, and histopathological examination of each group had no obviously abnormal alterations.

TQB2450 had certain immunogenicity. The antibody-positive rates in 50, 100, and 200 mg/kg groups were 10%, 10%, and 0, respectively, and were detected within 6-8 weeks after discontinuance. The exposure of TQB2450 in cynomolgus monkeys increased proportionally with the increasing dose between 50 mg/kg and 200 mg/kg. Besides, certain accumulation was observed after multiple doses.

TQB2450 was injected intravenously into cynomolgus monkeys for 4 weeks and suspended for 4 weeks. The no-observed-adverse-effect level (NOAEL) was 200 mg/kg (AUC0-t was 507909.7±139794.1 hr*μg/mL at this dose after 5 doses).

Table 1.2-2 Average toxicokinetic parameters of different doses of TQB2450 in cynomolgus monkeys

| Time | Dose (mg/kg) | | TQB2450 | | |
| --- | --- | --- | --- | --- | --- |
|  |  |  | 50 | 100 | 200 |
| First dose | C_max_ | μg/mL | 1112.9±166.7 | 2122.4±382.1 | 4120.8±476.4 |
|  | AUC_0-t_ | μg·h/mL | 69524.6±10057.9 | 136371.1±20213.0 | 272611.4±45291.7 |
| Last dose | C_max_ | μg/mL | 1663.5±325.1 | 3343.6±810.9 | 6062.6±1245.5 |
|  | AUC_0-t_ | μg·h/mL | 139614.2±27136.6 | 281160.2±97989.0 | 507909.7±139794.1 |
| Accumulation factor | AR | — | 2.0±0.4 | 2.0±0.5 | 1.9±0.4 |

1.2.2.3 Hemolysis test and vascular stimulation test

Hemolysis test

The hemolysis test consisted of a negative control group (0.9% sodium chloride injection), a positive control group (sterilization water for injection), and TQB2450 dose groups (0.1, 0.2, 0.3, 0.4, 0.5 mL/tube, 10 mg/mL, respectively). The mixture was composed of a set scale of 2% rabbit red blood cell suspension, TQB2450, 0.9% sodium chloride injection, and sterilization water for injection. The mixture was placed in the incubator at 37±0.5 ℃ and observed once at 0, 15, 30, 45, 60, 120, and 180 minutes, respectively. Make 3 parallel tubes in each group.

The results showed that after 3 hours at 37±0.5℃, hemolysis and coagulation were not observed in the negative control group, while hemolysis was presented in the positive control group. Besides, the supernatant was colorless and transparent in all TQB2450 sample groups. Red blood cells naturally sank and were redispersed following adequate oscillation. Hemolysis and coagulation were not observed in all TQB2450 sample groups.

Vascular stimulation test

The vascular stimulation test included the TQB2450 group with 4 male rabbits and 4 females. The highest planned concentration of 10 mg/mL was given by a single injection into the right auricular vein at the volume of 6 mL/kg. In parallel, the same volume of 0.9% sodium chloride injection as the control was administered through injection into the left auricular vein. The day of first dose was the first day of the study.

The general condition and injection site of rabbits were observed daily during the experiment. Four rabbits including 2 males and 2 females were euthanized and then dissected at ~72 hours and 16 days after injection, respectively. The injection site was observed by the naked eye, and the blood vessels and surrounding tissues of the injection site were examined by histopathology. A single injection of TQB2450 with a concentration of 10 mg/mL at a volume of 6 mL/kg was delivered in the ear vein of Japanese white rabbits, which had no irritation to the blood vessels and surrounding tissues of the injection site.

Comprehensive assessment

TQB2450 did not significantly change the electrocardiographic parameters, blood pressure, and respiratory rate of male and female monkeys.

The general observation, body mass, food intake, temperature, electrocardiogram, blood pressure, hemanalysis, serum biochemistry, urinalysis, and gross anatomy showed no obvious abnormalities following single intravenous doses of TQB2450 of 200 and 400 mg/kg. The MTD was 400 mg/kg.

TQB2450 was intravenously injected for 4 weeks and suspended for 4 weeks. The NOAEL was 200 mg/kg (AUC0-t was 507909.7±139794.1 hr*μg/mL at this dose after 5 doses).

Hemolysis and coagulation were not observed in all TQB2450 groups. TQB2450 had no irritation to the blood vessels and surrounding tissues of the injection site.

- - 1. Overview of preclinical pharmacokinetics of TQB2450

After single intravenous doses of TQB2450 at 1, 10, and 60 mg/kg, pharmacokinetic data in cynomolgus monkeys showed a linear dose-dependent relationship. Following multiple doses (10 mg/kg, once a week on weeks 1-4), there were no statistical differences in the peak time and half-life compared with a single dose. However, the peak concentration and drug exposure were higher than that of a single dose. Besides, the accumulation factor was 1.73±0.65 at the interval of 168 hr.

The radioactive distribution of TQB2450 was ranked by the area under the curve (AUC): serum, lung, liver, bone marrow, gonads, heart, adrenal gland, spleen, kidney, bladder, lymph nodes, small intestine, submandibular gland, fat, thymus, eyeball, large intestine, urine, pancreas, muscle, brain. Tissues and organs with abundant blood perfusion had greater radioactivity, such as the lung, liver, bone marrow, gonads, heart, etc. In contrast, organs with poor blood perfusion had less radioactivity, including fat, eyeballs, muscles, etc. Low radioactivity in the brain suggested that TQB2450 did not readily cross the blood-brain barrier.

TQB2450 was mainly excreted through urine and slightly through feces. The excretion rate of TQB2450 was slow.

- - 1. Overview of clinical research

1.2.4.1 Clinical Progress

This product is an innovative anti-PD-L1 fully humanized monoclonal antibody belonging to a novel sequence of monoclonal antibodies. It is classified as a Class 1 therapeutic biologic product. In October 2017, it obtained approval for clinical research from the China Food and Drug Administration (CFDA: 2017L04914). Currently, Phase I tolerability and pharmacokinetic studies are underway for the treatment of advanced malignant tumor patients. The dosing regimen includes intravenous infusion once every three weeks at doses of 1, 3, 10, 20, 30 mg/kg, and 1200 mg/time, with a 21-day cycle of treatment. Thirty-four subjects have been enrolled, and dose escalation has been completed without observing any dose-limiting toxicity (DLT). The study is currently in the expanded phase of fixed-dose administration.

1.2.4.2 Clinical Efficacy

Currently, 34 subjects have been enrolled, with 31 individuals completing the initial tumor assessment. Among them, two Hodgkin's lymphoma patients achieved a partial response (PR), one exited the study after 12 cycles, and one continues treatment after completing 14 cycles. Several patients with renal cancer, diffuse large B-cell lymphoma, lung cancer, and others showed stable disease (SD) as their best response. Based on the investigator's assessment, participants receiving TQB2450 could benefit, and therefore, continue treatment in the study. TQB2450 has shown preliminary efficacy, indicating promising activity, and further exploration across various indications is currently underway.

Summary of efficacy of TQB2450 in Phase I trial

| Group | Numbers | Efficacy |
| --- | --- | --- |
| 1mg/kg | 1 | Ended treatment after progressive disease (PD) |
| 3mg/kg | 6 | 4 PD (ended treatment), 2 still on treatment. Among them, 1 patient with Hodgkin's lymphoma achieved a best response of PR and ended treatment after completing 12 cycles;1 liver cancer patient and 1 lung cancer patient, both achieved a best response of SD after 7 cycles of treatments. |
| 10mg/kg | 6 | 4 PD (ended treatment), 2 still on treatment. Among them, 1 had diffuse large B-cell lymphoma and 1 had Hodgkin's lymphoma, and achieved a best response of SD and PR,respectively, after 15 cycles of treatments. |
| 20mg/kg | 3 | 3 all ended treatment. Among them, 1 renal cancer patient ended treatment after 8 cycles due to a serious adverse event (SAE), with the best response of SD. |
| 30mg/kg | 6 | 5 ended treatment. 1 renal cancer patient achieved a best response of SD after completing 9 cycles. |
| 1200mg | 12 | 5 ended treatment., 5 still on treatment. 1 melanoma patient achieved a first-time tumor assessment of SD after 6 cycles, 1 lung cancer patient achieved a first-time tumor assessment of SD after 6 cycles, and 1 colorectal cancer patient achieved a first-time tumor assessment of SD after 4 cycles. |

1.2.4.3 Safety

The 30 mg/kg group has enrolled a total of six subjects, and no DLT have been observed. A statistical analysis of adverse events for the 34 patients included in the study reveals adverse events occurring in four or more individuals. Safety data summary indicates that adverse events are primarily related to various laboratory abnormalities, with severity mainly grade 1-2, or consistent with the characteristics of underlying diseases. Therefore, TQB2450 demonstrates good tolerability and safety, with manageable overall adverse events that are generally consistent with adverse events observed with similar drugs.

Summary of adverse event of TQB2450 in phase I trial

| Adverse event (n=34) | Grade ≥3 | | Any grade | |
| --- | --- | --- | --- | --- |
|  | No. | Incidence, % | No. | Incidence, % |
| Patients with ≥1 AE | 7 | 20.59 | 30 | 88.24 |
| Laboratory Tests |  |  |  |  |
| GGT increased | 1 | 2.94 | 8 | 23.53 |
| AST increased | 2 | 5.88 | 8 | 23.53 |
| Serum creatinine increased | 0 | 0.00 | 6 | 17.65 |
| ALP increased | 1 | 2.94 | 6 | 17.65 |
| ALT increased | 1 | 2.94 | 5 | 14.71 |
| Amylase decreased | 0 | 0.00 | 5 | 14.71 |
| Amylase increased | 1 | 2.94 | 5 | 14.71 |
| Lipase decreased | 0 | 0.00 | 4 | 11.76 |
| White blood cell decreased | 2 | 5.88 | 4 | 11.76 |
| Blood bilirubin increased | 0 | 0.00 | 4 | 11.76 |
| Metabolic and nutritional disorders |  |  |  |  |
| Hyperuricemia | 1 | 2.94 | 5 | 14.71 |
| Hyponatremia | 1 | 2.94 | 5 | 14.71 |
| Hypoalbuminemia | 0 | 0.00 | 4 | 11.76 |
| Hypochloremia | 0 | 0.00 | 4 | 11.76 |
| Endocrine System Disorders |  |  |  |  |
| Hypothyroidism | 0 | 0.00 | 6 | 17.65 |
| Blood and Lymphatic System Disorders |  |  |  |  |
| Anemia | 2 | 5.88 | 8 | 23.53 |
| Blood and Lymphatic System Disorders |  |  |  |  |
| Pruritus | 0 | 0.00 | 6 | 17.65 |

GGT, gamma-glutamyl transferase; AST, aspartate aminotransferase; ALP alkaline phosphatase; ALT, alanine aminotransferase;

Summary of serious adverse event of TQB2450 in phase I trial

| Patient no. | Period | Dosage | Serious AEs | Association with the study drug |
| --- | --- | --- | --- | --- |
| 001 | C3 | 1mg/kg | Grade IV hypercalcemia | Possible |
|  |  |  | Type II respiratory failure | Unlikely |
| 008 | C3 | 20mg/kg | Grade III liver injury | Definite |
| 009 | C7 | 20mg/kg | Somnolence | Definite |
| 010 | C6 | 20mg/kg | Pancreatic amylase increased | Immune-Related AEs |
|  |  |  |  | Definite |
| 011 | C2 | 30mg/kg | Pleural effusion | Immune-Related AEs |
| 014 | C3 | 10mg/kg | Decreased appetite with bilateral lower limb edema | Unlikely |
| 021 | C6 | 3mg/kg | Liver dysfunction | Possible |
| 022 | C1 | 3mg/kg | Grade III febrile neutropenia | Probable |
| S043 | Screening | - | Pulmonary infection, blood pressure decreased, sudden cardiac arrest, patient died from respiratory failure after symptomatic treatment. | Unrelated |

1.2.3.4 Pharmacokinetic results

Currently, blood drug concentrations have been measured for the first six dose groups. Following intravenous administration of the drug, the results for serum drug exposure and Cmax indicate good linearity from 3 mg/kg to 30 mg/kg, with a half-life of approximately 320 hours for the 1200 mg dose group in the first cycle. When administered for 21 days, the majority of receptor occupancy has reached over 90%, suggesting that the drug can maintain saturation until the next administration, ensuring sustained efficacy and supporting the feasibility of administering the drug once every 3 weeks.

1.2.3.5 Dose recommendation

Taking into consideration the average weight of the Chinese population, receptor occupancy, probability of immunogenicity, clinical convenience, dosages of similar drugs, efficacy, and safety, a proposed fixed dose of 1200 mg per administration every 3 weeks is recommended for later-stage trials. This dosage will be further validated for safety and efficacy in subsequent trials.

1. Study objective

ALTER-L038 study aims to evaluate the efficacy and safety of the combination including anlotinib and TQB2450 in EGFR-positive advanced NSCLC patients who failed prior EGFR-TKIs therapies.

1. Study design
   1. Overall design

This is a multicenter, open-label, single-arm, phase I/II study aimed at evaluating the efficacy and safety of the combination including anlotinib and TQB2450 in EGFR-positive advanced NSCLC patients who failed prior EGFR-TKIs therapies.

The first stage of the study is a phase I 3+3 dose-escalation study, starting with 8 mg anlotinib in combination with 1200 mg TQB2450 as the initial dose, with three dose levels set at 8 mg/10 mg/12 mg. Phase I study is expected to enroll 18 participants.

The second stage is a multicenter, single-arm phase II study using the MTD determined in the first stage, with an estimated enrollment of 54 participants. This study started in August 2019, with recruitment concluding around August 2020, and the estimated end date is August 2021.

In the absence of withdrawal of consent, intolerable side effects, or a decision by the investigator to be inappropriate for further trial, treatment is continued for each subject until radiographically confirmed disease progression.

- 1. Type of comparison

The phase I study is designed for descriptive statistics without comparative analysis.

The phase II study is a single-arm study using historical controls. The primary efficacy endpoint is PFS. A statistically significant extension in PFS compared to historical controls, reaching a clinically meaningful difference (defined as a PFS extension of ≥4 months when comparing the trial and historical groups, using 30.5 calendar days as a month), would be considered a positive outcome for the study.

- 1. Sample size calculation

Phase I part: Following the 3+3 dose-escalation design, the study anticipates a minimum of 3 and a maximum of 18 participants to be enrolled.

Phase II part: Based on prior efficacy data achieved with carboplatin plus pemetrexed in EGFR+ NSCLC patients who failed to EGFR-TKI, an mPFS of 5 months was utilized as a historical control to determine the sample size for the phase II part. We hypothesized that TQB2450 plus anlotinib would lead to clinical benefit in this population, with a mPFS of 9 months. Sample sizes of 54 in the study achieved 90% power at a significance level (α) of 0.05 with an anticipated dropout rate of 20%. The study is expected to last for 24 months, including a 12-month patient enrollment period. At the study's conclusion, an estimated 32 endpoint events are expected to occur.

1. Subjects
   1. Diagnostic criteria

Diagnostic criteria: the diagnosis will be confirmed according to the sixth edition of Oncology Diagnostics and the sixth edition of Internal Medicine published by the People's Medical Publishing House of the People's Republic of China.

- 1. Inclusion criteria

The inclusion criteria were:

1. Male or female, aged 18-75 years;
2. Histologically and cytologically confirmed locally advanced/advanced NSCLC, assessed as stage IIIB-Ⅳ NSCLC (according to the 8th edition of the tumor–node–metastasis staging system of the American Joint Committee on Cancer), or NSCLC patients with postoperative recurrence (all types);
3. Patients with EGFR-positive (EGFR+) NSCLC with failure of prior EGFR-TKIs, or *T790M* mutation with failure of prior 3^rd^-generation EGFR-TKIs;
4. EGFR-TKIs should be the mainstay of the initial treatment for patients. Patients are allowed to receive ≤1 systemic chemotherapy;
5. Patients with previous only 1^st^/2^nd^-generation EGFR-TKIs were required to provide genetic testing reports showing no primary or acquired T790M mutation; patients with previous 3^rd^-generation EGFR TKIs were required to provide genetic testing reports showing EGFR mutation was positive;

Treatment failure is defined as (1) the PD assessed by imaging or clinical evidence during or after EGFR TKIs treatment, or withdrawal from standard treatment for intolerant AEs. The intolerant AEs included grade Ⅳ hematologic toxicity, or grade ≥Ⅲ nonhematologic toxicity, or grade ≥Ⅱ main organ damage such as heart, liver, and kidneys per Common Terminology Criteria for Adverse Events (CTCAE) version 5.0.

Detection results from other hospitals were also acceptable. Tissue is the first choice for detection samples, if optional blood samples cannot be obtained from tissue, the detection methods can be selected by traditional genetic testing (such as immunohistochemistry, ddPCR, Arms-PCR, etc.) or high-throughput sequencing.

1. At least one measurable lesion according to Response Evaluation Criteria in Solid Tumors (RECIST) version 1.1, without prior radiotherapy in the past three months;
2. Predicted life expectancy of ≥3 months; Eastern Cooperative Oncology Group (ECOG) performance status (PS) of 0-1;
3. Recovered from damages (≤grade 1) associated with other treatments per the CTCAE version 5.0;
4. Adequate function of the important organs as evident by the following criteria:
5. Hemanalysis (no blood transfusion, and without the use of G-CSF or other hematopoietic stimulating factors within 14 days):
6. Hemoglobin (HB) ≥80g/L;
7. Absolute neutrophil count (ANC) ≥1.5×10^9/L;
8. Platelets (PLT) ≥75×10^9/L;
9. Biochemistry:
10. Total bilirubin (TBIL) ≤upper limit of normal value (ULN);
11. ALT and AST ≤1.5×ULN;
12. Creatinine (Cr) ≤1.5×ULN or creatinine clearance rate (CCr) ≥60 mL/min;
13. Urinary protein <++;
14. Coagulation function: INR and APTT ≤1.5×ULN;
15. Doppler ultrasound assessment: left ventricular ejection fraction (LVEF) ≥50%;
16. Women of childbearing potential must have adopted contraception, or have a negative serum and urine pregnancy test within 7 days before the study enrollment; women of childbearing potential and men must agree to contraception for the duration of study treatment and 8 weeks after the last dose of study treatment, or have been of sterilization;
17. Be willing and able to provide written informed consent for the trial, and comply with all aspects of the protocol.
    1. Exclusion criteria

The exclusion criteria were:

1. Patients with a history of malignant tumors within 5 years before the start of treatment except for patients with cured orthotopic cervical cancer, cutaneous basal cell carcinoma, cutaneous squamous cell carcinoma, or superficial bladder cancer (Ta [non-invasive cancer], Tis [orthotopic cancer], T1 [superficially invasive]);
2. Central squamous cell lung cancer or squamous cell lung cancer with a significant pulmonary cavity;
3. Have received >1 systemic chemotherapy during previous lines of treatment (patients with neoadjuvant or adjuvant chemotherapy were allowed to be enrolled);
4. Have previously received other anti-PD-1/PD-L1 drugs or immunotherapies targeted PD-1/PD-L1;
5. Patients with severe hypersensitivity reaction after administration of other monoclonal antibodies;
6. Patients with anlotinib allergy;
7. Patients with active autoimmune disease or a history of autoimmune disease (such as, but not limited to, autoimmune hepatitis, interstitial pneumonia, enteritis, vasculitis, and nephritis; patients with asthma that needed bronchodilators for medical intervention were unable to be enrolled); patients with vitiligo, psoriasis, alopecia, and well-controlled type 1 diabetes mellitus who did not require systemic treatment were allowed to be enrolled;
8. Patients who had clinically significant thyroid dysfunction within 6 months before enrollment and whose thyroid function did not return to normal or was clinically insignificant despite medical therapy;
9. Factors that have a significant impact on oral drug absorption, such as inability to swallow, chronic diarrhea, and intestinal obstruction;
10. Patients with brain metastases with symptoms or less than 14 days of symptom control;
11. Severe comorbidities before enrollment:
12. Unstable angina and/or congestive heart failure or vascular disease within 12 months requiring hospitalization (such as aortic aneurysm requiring surgical repair or peripheral venous thrombosis), or other heart damages judged by investigators that could influence the safety assessment of study drugs (such as uncontrolled arrhythmia, myocardial infarction, or ischemia);
13. Esophagogastric varices, unhealed ulcers, uncured wounds, or fracture within 6 months;
14. History of abdominal fistula, gastrointestinal perforation, intra-abdominal abscess, or gastrointestinal bleeding within 6 months;
15. Arterial thromboembolism, grade ≥3 venous thromboembolism, transient ischemic attack (TIA), cerebral vascular accident (CVA), hypertensive crisis, or hypertensive encephalopathy within 6 months;
16. Aggravating chronic obstructive pulmonary disease (COPD) or other respiratory illness requiring hospitalization within 28 days;
17. An active lung infection and/or acute bacterial or fungal infection requiring intravenous antibiotic treatment within 28 days;
18. Clinical jaundice caused by abnormal liver function within 7 days;
19. Hypertension that was uncontrollable with a single antihypertensive drug at present (systolic blood pressure ≥160 mmHg or diastolic blood pressure ≥100 mmHg); or being treated with a combination of two or more antihypertensive drugs;
20. Major surgical operation, biopsy, or obvious traumatic injury within 28 days before enrollment;
21. Virologic testing during screening shows any of the following:
22. HBsAg positive, and HBV DNA ≥1×10^3^ copies/mL;
23. Anti-HCV positive and HCV >ULN;
24. HIV-positive;
25. Immune checkpoint inhibitors or systemic therapies were required to achieve immunosuppression (prednisone or other hormones >10 mg/d);
26. Patients with other anti-cancer therapies or participated in other clinical studies (not EGFR-TKI) within 4 weeks before the first dose of the drug; patients who received therapies or participated in other clinical studies of EGFR TKI within 2 weeks before the first dose of the drug;
27. Imaging showed that the tumor had invaded the important blood vessels, or the tumor was likely to invade the important blood vessels during the subsequent study and cause massive hemorrhage judged by investigators;
28. Patients with any bleeding signs or a history of bleeding, regardless of severity, having grade ≥3 bleeding events per CTCAE, unhealed wounds, anabiosis, or fracture within 4 weeks before grouping;
29. Clinically hemoptysis (daily hemoptysis greater than 50ml) within 2 months before enrollment (defined as coughing up ≥1 teaspoon of blood, small blood clots, or coughing up blood without sputum); but patients with blood in sputum were not excluded;
30. A history of psychotropic substance abuse and cannot be abstinent, or patients with mental disturbance;
31. Have previously received antiangiogenic drugs;

Conditions that increased the risk associated with study participation or the study medication, and could make a patient ineligible for study in the investigator's judgment.

- 1. Screening failure

Failure to establish eligibility within 28 days would result in screening failure and the subject will be excluded from the trial; however, the investigator may decide whether the subjects should undergo appropriate observation or management for re-screening and whether the subjects can be re-entered in the study. In this case, a new ICF will be required to be signed by the subject. Each participant is allowed a maximum of one opportunity for re-screening.

- 1. Withdrawal criteria
     1. Decisions to withdraw from the study by the investigator

The investigator has the right to decide whether to withdraw from the study if a subject who has been enrolled in the study has any situation that is not suitable for continuing the study:

1. Participants who do not meet the screening criteria;
2. Participants who are unable to evaluate for efficacy or unable to evaluate safety without receiving 1 dose of the drug;
3. Participants who are treated with chemotherapy, surgical or experimental drugs other than this protocol during the trial including CFDA-approved modern herbal agents and immunomodulatory agents for the treatment of tumors
4. Participants who do not follow the dose, method, and course of treatment according to the study protocol with poor compliance using drug amounts outside the range of 80% to 120% of the prescribed dose.
5. Occurrence of intolerable adverse reactions or severe adverse events confirmed by the investigator.
6. Termination of treatment decided by the investigator from the patient's best interests.
   - 1. Decisions to withdraw from study by the subject

According to the CIF, the subjects participate in the trials voluntarily and have the right to withdraw at any stage of the trial without discrimination or retaliation and without affecting their medical treatment, rights, and interests; participants who were no longer receiving medications, leading to lost to follow-up without explicit withdrawal from the study, are considered as “withdrawal” or “dropout”. There are possible reasons for withdrawal from study:

- 1. Patient withdrawal of informed consent.
  2. The patient lost to follow-up for various reasons
     1. Subsequent data collection

The investigator must clearly document the reasons and timing for a participant's withdrawal, perform relevant observations and evaluations, complete the assessment according to the study's withdrawal criteria, and record these details in the relevant source documents, detailing the cause of withdrawal.

Subjects who discontinue the study should continue to be monitored for AEs. If feasible, these subjects should be followed up for AEs until resolution to baseline levels or when the investigator determines it unnecessary to continue monitoring. In cases of chronic conditions such as AEs, the investigator might agree to discontinue further follow-up. In such instances, the investigator must document their decision in the subject's original medical records and promptly inform the sponsor about the participant's study discontinuation

During the study, participants who received medication but did not complete the required study assessments should have their final evaluation based on their last available data. Investigators are to contact participants who missed follow-up visits and document the evidence of contact in relevant source documents and Electronic Data Capture (EDC) systems (e.g., timestamps and dates of phone calls, mailing receipts), ensuring proper record-keeping.

For the following reasons leading to withdrawal, subsequent data collection requirements are waived:

1. Withdrawal of informed consent by the participant
2. Inability to complete follow-ups as scheduled for various reasons
3. Death
4. Sponsor's termination of the study

All patients must read and sign an informed consent approved by the ethics committee before the initiation of the study. All study procedures are required to be performed according to the time window in the schematic flow diagram, which is independent of the duration of drug discontinuance. However, the study procedures are allowed for modification within the time window due to holidays or other reasons.

1. **Study drugs**
   1. **Basic information about the study drugs**

**Table 5.1-1 Basic information about the study drugs**

| **Drug names** | **Specification** | **Storage conditions** | **Manufacturers** |
| --- | --- | --- | --- |
| TQB2450 injection | 100 mg/10 mL  300 mg/10 mL | Store, transport, protected from light, at 2-8°C | Chia-tai Tianqing Pharmaceutical Co., Ltd. |
| Anlotinib hydrochloride capsules | 12 mg/capsule  10 mg/capsule  8 mg/capsule | Sealed, protected from light, below 25°C | Chia-tai Tianqing Pharmaceutical Co., Ltd. |

The above drugs are produced by Chia-tai Tianqing Pharmaceutical Co., Ltd., and the production lot numbers and expiration dates of specific drugs are detailed in the drug test reports.

- 1. **Packaging of the study drugs**

The research drug package contains the name of the research drug, drug lot number, specifications, storage conditions, expiration date, name of the sponsor, and other information, and states “for clinical research use only”. The sample label is as follows, specific to the actual use shall prevail.

| **TQB2450-Ib-11** | |
| --- | --- |
| **(For clinical research use only)** | |
| Drug names: | TQB2450 injection |
| Specifications: | ×××mg/10ml |
| Usage and dosage: | ×× |
| Storage conditions: | Store, transport, protected from light, at 2-8°C |
| Production lot number: | ×× |
| Expiration date: | ×× |
|  | Provided by Chia-tai Tianqing Pharmaceutical Co., Ltd. |

| **Anlotinib** | |
| --- | --- |
| **(For clinical research use only)** | |
| Drug names: | Anlotinib hydrochloride capsules |
| Specifications: | Capsule ×× mg, 14 capsules per box, aluminum foam plate |
| Usage and dosage: | Oral on an empty stomach before breakfast, 1 capsules daily, oral once daily on days 1-14 |
| Storage conditions: | Sealed, protected from light, below 25°C |
| Production lot number: | ×× |
| Expiration date: | ×× |
|  | Provided by Chia-tai Tianqing Pharmaceutical Co., Ltd. |

**Table 5.2-1 The sample label of the research drug**

- 1. **Management of the study drugs**

The co-organizer will provide an adequate amount of study drugs to ensure the completion of the study. In accordance with Good Clinical Practice (GCP) requirements, the research drug will be kept in the exclusive custody of the drug administrator and stored under appropriate storage conditions. Study drugs are dispensed as prescribed or medical advice, and the last remaining research drug is collected. Complete records are required for the dispensing and recall of study drugs.

- - 1. **First drug distribution**

After the subjects meet the requirements for enrollment in this study, the investigator will formulate a prescription or medical advice based on the randomized results. The medication manager dispenses the study medication according to the prescription or medical advice.

- - 1. **Distribution and recall of study drugs during follow-up**

TQB2450: TQB2450 was distributed according to the prescription or medical advice, and the subjects were given medication according to the protocol. After the completion of medication, the unopened TQB2450 was recovered.

Anlotinib hydrochloride capsules: In the course of the study, according to the frequency of visits of the subjects, according to the cycle, the anlotinib hydrochloride capsules of the next stage were distributed to the subjects at the end of the cycle, and the unused drugs and remaining drug packages in this stage were recalled.

During the study, TQB2450 and anlotinib hydrochloride capsules were not dispensed to subjects if they were considered by the investigator to be unsuitable for continued medication, and unused medication and drug packages were recalled.

- - 1. **Disposal or destruction of leftover drugs**

The used or partially used drug containers, bottles, infusion bags, and syringes of TQB2450can be locally disposed of according to the usage guidelines and operating procedures established by the study center and local institutions. Unopened drugs should be handed over to the co-organizer for disposal or destruction after the trial.

For the anlotinib hydrochloride capsules that have been distributed to the subjects, the remaining drugs of the last time should be recalled. For the drugs that have not been distributed to the subjects, they should be handed over to the co-organizers for disposal or destruction after the end of the trial together with the remaining drugs recovered during the trial.

- 1. **TQB2450preparation method**

Aseptic principles should be strictly followed during the preparation process. Under aseptic conditions, extract the desired dose of TQB2450from the glass bottle and aseptically dilute it with sterile, non-heated physiological saline to a total volume of 250ml in an infusion bag. Gently invert the solution without shaking to ensure adequate mixing.

As an injectable medication, it should be visually inspection for the presence of particulate matter and discoloration before administration. The drug is a clear to slightly opalescent, colorless to pale yellow liquid. If the solution appears cloudy, discolored, or in the presence of visible particles, it should be discarded. Do not shake the vial.

TQB2450 does not contain preservatives and IV bags containing TQB2450 must be used immediately after dilution/mixing. If the TQB2450 dilution cannot be used immediately, the method of storage is as follows:

- The total storage time from opening the TQB2450 vial to the end of injection should not exceed 6 hours at room temperature;
- The total storage time should not exceed 24 hours in the refrigerator of 2 ℃-8 ℃ without freezing and shaking.
  1. **Administration regimens of the study drugs**
     1. **Administration principles**

During the study period, every 21 days will be one treatment cycle. More than 3 days per treatment cycle will be considered as reasonably delayed dosing, the subsequent administration time was calculated according to the actual date of the previous administration. No other anticancer therapy was allowed during the medication. Patients with the disease control (CR+PR+SD) and who can tolerate adverse effects continue the medication until disease progression or intolerance.

- TQB2450: On the first day of each cycle (D1), a fixed dose of 1200mg will be administered intravenously using an infusion set with a filter membrane, and infused for 60 ±10 minutes. The infusion time starts with the beginning of the TQB2450 infusion and ends with the completion of the TQB2450 infusion and normal saline flushing.
- Anlotinib hydrochloride capsules: According to the 3+3 dose climbing test, the maximum tolerated dose was determined as the initial dose. Before breakfast, take anlotinib orally on an empty stomach( TQB2450 was administered on an empty stomach within±5min of the start of infusion on day 1 of each cycle ),1 tablet per day. Continue the drug administration for 2 weeks and stop for 1 week. If there are missed medications and the interval from the next medication is <12 h, no medication is supplemented. If there was a vomiting event during the treatment, it was necessary to take the medicine if there were formed capsules in the vomit. If no capsule was formed, no supplement was given.
  - 1. **Infusion reaction processing**

Infusion-related reactions may occur with the administration of TQB2450. Symptoms may include fever, chills, rigors, sweating, and headache.

To prevent infusion reactions, H1 blockers (diphenhydramine 50mg IV, or equivalent) and acetaminophen (500 to 650mg oral or IV) can be administered 30-60min before each infusion of TQB2450. Systemic glucocorticoid therapy for the control of infusion reactions or immune-related adverse events had to be tapered for at least 2 weeks before the next dose of study drug at a dose that did not suppress immune-system function (prednisone or equivalent, ≤10mg per day).

Potential infusion-related reactions were observed during infusion of TQB2450 and 30min after completion of infusion.

Infusion rate regulation and/or treatment interruption/cessation for infusion-related reactions depends primarily on severity, as shown in the table:

**Table 5.5-1 Adjustment measures for infusion reaction**

| Grades of AEs  (NCI-CTCAE v5.0) | Adjustment measures |
| --- | --- |
| Grade 1- mild  It is not recommended to interrupt the infusion and clinical intervention for transient mild reactions | The rate of drop was reduced by 50% and any worsening symptoms were closely observed, and clinical intervention was performed if necessary. |
| Grade 2- moderate  Treatment or fluids should be suspended and systemic therapy (e.g., antihistamines, nsaids, anesthetics, intravenous fluids) should be given immediately. Prophylactic therapy is recommended for 24 hours or less. | Treatment with TQB2450 was suspended and resumed when the infusion reaction had resolved to grade 0-1 with a 50% reduction in the infusion rate. During this period, any worsening symptoms were closely observed and appropriate therapeutic interventions were initiated according to local medical practices. |
| Grade 3- severe  Symptoms persist, such as a slow response to systemic therapy and suspension of infusions, relapses after rapid resolution, and hospital admission due to complications. | Treatment with TQB2450 was immediately and permanently discontinued, and appropriate therapeutic interventions were implemented according to local practice. |
| Grade 4- life-threatening and requiring urgent medical intervention |  |

If the infusion rate of TQB2450 is reduced by 50% (90±10min) because of an infusion reaction, this reduction must be maintained for the next scheduled infusion. If an infusion reaction was not observed at the next scheduled infusion, the infusion rate could be returned to the baseline rate during subsequent infusions.

- - 1. **Adjustment of administration regimens**
       1. **General principles for adjustment of administration regimens**

When a toxic reaction occurs, the investigator may refer to the following rules for delay or dose adjustment. The reason for the delay or dose adjustment, the supportive therapy taken and the results should be documented in the subject's medical record and EDC.

If the investigator determines that the dosing adjustment differs from the proposed adjustment rule, after consultation and communication with the sponsor, the investigator will make the appropriate adjustment with reference to clinical practice guidelines or previous clinical experience, and the relevant reasons will be recorded in the subject’s medical record.

- Toxic reactions should be graded according to the NCI CTCAE 5.0;
- In case of simultaneous occurrence of severe toxicity of different severity levels, adjustments should be made according to the highest level observed;
- Treatment delays due to toxicity caused by anlotinib capsules could be delayed up to 35 days after the start of day 1 of the cycle (i.e., a 14-day delay in planned treatment) to allow adequate time for the subject to recover from any toxic effects caused by anlotinib hydrochloride. If a subject is unable to resume treatment by day 35 after the last dose, the subject must discontinue anlotinib hydrochloride treatment;
- Treatment delays due to toxic reactions caused by TQB2450 could be delayed up to 12 days after the start of day 1 of the treatment cycle;
- If subjects require permanent discontinuation due to toxic reactions (e.g., immune-related pneumonitis) of TQB2450, monotherapy with anlotinib should be continued until disease progression or unacceptable toxicity (whichever occurs first);
- If subjects require permanent discontinuation due to toxic reactions (hypertension, proteinuria, etc.) of anlotinib, monotherapy with TQB2450 should be continued until disease progression or unacceptable toxicity (whichever occurs first);
  - - 1. **Criteria for Dose Delay**

All of the following conditions should be met before starting treatment in any cycle, otherwise, a delay in administration is required:

- Absolute neutrophil count ≥1.5×109/L;
- Platelet count ≥75×10^9^/L;
- Hemoglobin（Hb）≥80g/L;
- Proteinuria ≤2+;
- Hand-foot skin reaction and hypertension ≤grade 2;
- Other toxicities until recovery to ≤grade 1

During the administration period (1-14 days) of anlotinib every cycle, if toxic reactions occur and the medication needs to be delayed, the maximum delay time should not exceed 5 days. If the medication cannot be continued for more than 5 days, the remaining anlotinib will not be administrated in this cycle.

- - - 1. **Dose modification**

Dose modifications for the TQB2450 were not allowed in principle throughout the study.

For anlotinib hydrochloride capsules, according to the 3+3 dose climbing test, the maximum tolerated dose was determined as the initial dose. If the initial dose is 12mg, dose level 1 is 10mg, and dose level 2 is 8mg; If the initial dose is 10mg, dose level 1 is 8mg; If the initial dose is 8mg, there was no down-dose selection. Any subject who required a reduction in the anlotinib capsule dose would continue to receive the reduced dose for subsequent cycles. Any subject who has undergone two dose reductions and requires a third reduction due to toxic reactions must discontinue the study treatment.

Principles of dose modifications related to anlotinib hydrochloride capsules.

- Hematologic toxicity

1. Thrombocytopenia

**Table 5.5-2 Dose adjustments related to anlotinib hydrochloride capsules-** **the Platelet counts decreased**

| Grade of AEs | Recommended actions | Adjustment of administration regimens |
| --- | --- | --- |
| Grade 1 | Planned visits | Maintain the original dose |
| Grade 2 | Delayed dosing until recovery to ≤grade 1 | Maintain the original dose |
| Grade 3 | Delayed dosing and medical intervention treatment until recovery to ≤grade 1 | reduce 1 dose level |
| Grade 4 | Permanent termination of treatment | / |

1. Neutropenia

**Table 5.5-3 Dose adjustments related to anlotinib hydrochloride capsules-** **the neutrophil count decreased**

| Grade of AEs | Recommended actions | Adjustment of administration regimens |
| --- | --- | --- |
| Grade 1 | Planned visits | Maintain the original dose |
| Grade 2 | Delayed dosing until recovery to ≤grade 1 | Maintain the original dose |
| Grade 3-4 | Delayed dosing and medical intervention treatment until recovery to ≤grade 1 | reduce 1 dose level |

- Nonhematologic toxicity

1. Abnormal liver function (elevated ALT, AST, or TBIL)

**Table 5.5-4 Dose adjustments related to anlotinib hydrochloride capsules- abnormal liver function**

| Grade of AEs | Recommended actions | Adjustment of administration regimens |
| --- | --- | --- |
| Grade 1 | Planned visits | Maintain the original dose |
| Grade 2 | Delayed dosing until recovery to ≤grade 1 | Maintain the original dose |
| Grade 3-4 | Delayed dosing and medical intervention treatment until recovery to ≤grade 1 | reduce 1 dose level |

1. Proteinuria

**Table 5.5-5 Dose adjustments related to anlotinib hydrochloride capsules- proteinuria**

| Grade of AEs | Definition | Recommended actions | Adjustment of administration regimens |
| --- | --- | --- | --- |
| Grade 1 | Proteinuria 1+, or urinary protein < 1.0g/24 hours | Planned visits | Maintain the original dose |
| Grade 2 | Proteinuria 2+, and urinary protein < 1.0g/24 hours | Planned visits | Maintain the original dose |
|  | Proteinuria 2+, and urinary protein 1.0-3.4g/24 hours | Delayed dosing and medical intervention treatment until proteinuria ≤ 2+ and urinary protein < 1.0g/24 hours | Maintain the original dose |
| Grade 3 | Urinary protein≥3.5g/24hours | Delayed dosing and medical intervention treatment until proteinuria ≤ 2+ and urinary protein < 1.0g/24 hours | reduce 1 dose level |

1. Bleeding events (including hemoptysis, gastrointestinal bleeding, epistaxis, bronchial bleeding, gingival bleeding, gross hematuria, fecal occult blood, and cerebral hemorrhage).

**Table 5.5-6 Dose adjustments related to anlotinib hydrochloride capsules-bleeding events**

| Grade of AEs | Recommended actions | Adjustment of administration regimens |
| --- | --- | --- |
| Grade 1 | Planned visits | Maintain the original dose |
| Grade 2 | Delayed dosing until recovery to ≤ grade 1 | reduce 1 dose level, if it recurs, permanent termination of treatment is considered |
| ≥Grade 3 | Permanent termination of treatment | / |

1. Hypertension

**Table 5.5-7 Dose adjustments related to anlotinib hydrochloride capsules-hypertension**

| Grade of AEs | Recommended actions | Adjustment of administration regimens |
| --- | --- | --- |
| Grade 1-2 | Planned visits | Maintain the original dose |
| Grade 3 | Delayed dosing until recovery to ≤ grade 2 | reduce 1 dose level |
| Grade 4 | Permanent termination of treatment | / |

1. Hand-foot skin reaction

**Table 5.5-8 Dose adjustments related to anlotinib hydrochloride capsules-hand-foot skin reaction**

| Grade of AEs | Recommended actions | Adjustment of administration regimens |
| --- | --- | --- |
| Grade 1-2 | Planned visits | Maintain the original dose |
| Grade 3-4 | Delayed dosing until recovery to ≤ grade 2 | reduce 1 dose level |

1. Others

**Table 5.5-9 Dose adjustments related to anlotinib hydrochloride capsules-**o**ther nonhematologic toxicities**

| Grade of AEs | Recommended actions | Adjustment of administration regimens |
| --- | --- | --- |
| Grade 1 | Planned visits | Maintain the original dose |
| Grade 2-3 | Delayed dosing until recovery to ≤ grade 1 | reduce 1 dose level |
| Grade 4 | Permanent termination of treatment | / |

- - - 1. **Treatment of immunogenic adverse reactions**

Similar to all monoclonal antibody therapies, there is a risk of allergic reactions, including anaphylactic shock, with TQB2450. The TQB2450 should be administered to the subject in an environment where immediate emergency treatment can be performed.

If adverse events related to the investigational drug occurred during the trial, the investigator should decide whether to suspend the trial drug and take symptomatic treatment according to the specific situation. It is recommended to handle the adverse event according to the table. In case of adverse reactions not specified in the immunogenic adverse reaction management table, the expert consensus management recommendations for toxicity management related to SITC immune checkpoint inhibitors (Annex VI) can be managed.

**Table 5.5-10 Treatment of immunogenic adverse reactions**

| Immunologically adverse reactions related to the trial drugs | Severity (CTC grade of AEs) | Clinical Management |
| --- | --- | --- |
| Respiratory diseases  (e.g., interstitial pneumonia) | Grade 2 | Suspend treatment and provide symptomatic treatment with glucocorticoids until toxicity subsides to Grade 1 or lower. If toxicity does not recover within 12 weeks after the last administration, treatment should be terminated. |
|  | Grade 3 or worse, or recurrence | Discontinuation of treatment |
| Hepatitis | Grade 2 (3×ULN < ALT or AST < 5×ULN, or 1.5×ULN < total bilirubin < 3×ULN), lasts for more than 5 days | Suspend treatment and provide symptomatic treatment with glucocorticoids until toxicity subsides to Grade 1 or lower. If toxicity does not recover within 12 weeks after the last administration, treatment should be terminated. |
|  | Grade 3 or worse (ALT or AST≥5×ULN, or total bilirubin ≥ 3×ULN） | Discontinuation of treatment |
| Colitis or diarrhea | Grade 2 or 3 | Suspend treatment and provide symptomatic treatment with glucocorticoids until toxicity subsides to Grade 1 or lower. If toxicity does not recover within 12 weeks after the last administration, treatment should be terminated. |
|  | Grade 4 | Discontinuation of treatment |
| Pancreatitis | Grade 2 or 3 pancreatitis  Grade 2 or 3 elevated amylase or lipase | Suspend treatment and provide symptomatic treatment with glucocorticoids until toxicity subsides to Grade 1 or lower. If toxicity does not recover within 12 weeks after the last administration, treatment should be terminated. |
|  | Grade 4 or recurrent pancreatitis | Discontinuation of treatment |
| Endocrine dysfunction | Grade 2 or 3 hypophysitis | Suspend treatment and provide symptomatic treatment with glucocorticoids until toxicity subsides to Grade 1 or lower. If toxicity does not recover within 12 weeks after the last administration, treatment should be terminated. |
|  | Grade 4 hypophysitis | Discontinuation of treatment |
|  | Hyperglycemia of grade 3 or higher (fasting blood-glucose >13.9-27.8 mmol/L） | Suspend treatment and use insulin replacement therapy until blood glucose levels recover to Grade 2 or below and remain stable. |
|  | Symptomatic hypothyroidism Symptomatic hyperthyroidism, or asymptomatic hyperthyroidism with TSH＜0.1mIU/L | Suspend treatment and use relevant symptomatic treatment until the relevant indicators recover to the inclusion criteria. |
|  | Adrenal insufficiency of grade 2 or higher | Suspend treatment and provide symptomatic treatment with glucocorticoids until toxicity subsides to Grade 1 or lower. If toxicity does not recover within 12 weeks after the last administration, treatment should be terminated. |
| Encephalitis or meningitis | All grades | Discontinuation of treatment |
| Motor and nervous system diseases | Grade 2 | Suspend treatment and provide symptomatic treatment with glucocorticoids until toxicity subsides to Grade 1 or lower. If toxicity does not recover within 12 weeks after the last administration, treatment should be terminated. |
|  | Grade 3 or worse, or recurrence | Discontinuation of treatment |
|  | All grades of Guillain-Barre syndrome | Discontinuation of treatment |
|  | All grades of myasthenia gravis | Discontinuation of treatment |
| Diseases of the skin and subcutaneous tissue | Grade 2 or 3 | Suspend treatment and provide symptomatic treatment with glucocorticoids until toxicity subsides to Grade 1 or lower. If toxicity does not recover within 12 weeks after the last administration, treatment should be terminated. |
|  | Grade 4 | Discontinuation of treatment |
| Eye diseases | Grade 2 | Suspend treatment and provide symptomatic treatment with glucocorticoids until toxicity subsides to Grade 1 or lower. If toxicity does not recover within 12 weeks after the last administration, treatment should be terminated. |
|  | Grade 3 or worse, or recurrence | Discontinuation of treatment |
| Other immune-related adverse events | Grade 2 or 3 | Suspend treatment and provide symptomatic treatment with glucocorticoids until toxicity subsides to Grade 1 or lower. If toxicity does not recover within 12 weeks after the last administration, treatment should be terminated. |
|  | Grade 4 | Discontinuation of treatment |

According to reports from clinical trials of similar drugs abroad, patients receiving PD-L1 inhibitors have a risk of infection, including sepsis, herpes encephalitis, and retroperitoneal hemorrhage caused by mycobacterial infection. Therefore, patients need to be monitored for signs and symptoms of infection in clinical trials. Investigators should consider whether to withhold antibiotics and use antibiotics until the subjects with suspected or confirmed grade 2 bacterial infection have resolved to grade 1 or lower and if the patients with grade 3 infection or grade 2 infection have not resolved for 14 days, the treatment should be discontinued.

For subjects with liver metastases, baseline AST or ALT, or bilirubin elevations, treatment should be discontinued if the increase in AST, ALT, or bilirubin was ≥50% relative to that before the adverse event and lasted for at least 1 week.

- 1. **Concomitant medications**

Any protocol-allowed medications taken by subjects for concomitant disease (except for study drugs) during the trial are concomitant medications and will be recorded on the medical record and eCRF. Concomitant medications were recorded from the time the subjects signed the informed consent form until 28 days after the end of the study drug or the initiation of additional antineoplastic therapy (whichever occurs first).

- - 1. **Prohibited or cautiously given concomitant medications**

Subjects should not receive any approved medications with antitumor effects during the treatment period, such as chemotherapy, traditional Chinese medicine, and immunoregulation (thymosin, interferon, interleukin-2, purple dragon, lentinan, etc.).

Subjects should be cautiously given anticoagulant or clot-preventing medications during the treatment period to avoid exacerbating potential bleeding risk, including but not limited to Salicylic acid derivatives (e.g., aspirin); heparins (e.g., low molecular weight heparin, enoxaparin, dalteparin sodium, ardeparin sodium, etc.); Preventive anticoagulant drugs after cardiovascular events (e.g., clopidogrel and ticagrelor).

Subjects should be cautiously given CYP3A inducers (carbamazepine, rifampicin); phenobarbital inhibitors (ketoconazole, itraconazole, erythromycin, and clarithromycin); CYP3A4 substrate (simvastatin, cyclosporine, and piperidine); other medications metabolized by CYP3A4 (benzodiazepines, dihydropyridine calcium antagonist [calcium antagonism may be selected as appropriate for uncontrolled ACEI hypertension]; HMG-COA reductase inhibitor); CYP2C9 substrate (diclofenac, phenytoin sodium, piroxicam, S-warfarin, and tolbutamide); CYP2C19 substrate (diazepam, imipramine, lansoprazole, and S-mephenytoin). Use CYP2C9 and CYP2C19 substrates with caution, as detailed in the table below.

| P450 enzymes | Substrate |
| --- | --- |
| CYP2C9 | diclofenac, phenytoin sodium, piroxicam, S-warfarin, and tolbutamide |
| CYP2C19 | diazepam, imipramine, lansoprazole, and S-mephenytoin |

Citrus, star fruit, grapefruit, and grapefruit juice affect cytochrome P450 activity and should be avoided in combination.

Since Tinib-related drugs have the side effects of prolonging QT interval in clinical practice, it is necessary to use drugs that prolong QT interval with caution during the trial. This includes, but is not limited to, the several classes of medicines listed below:

- Antimicrobials (clarithromycin, azithromycin, erythromycin, roxithromycin, metronidazole, moxifloxacin);
- Antiarrhythmic drugs (quinidine, sotalol, amiodarone, disopyramide, procaine amide);
- Antipsychotics (risperidone, fluphenazine, haloperidol, haloperidol, thioridazine, pimozide, olanzapine, clozapine);
- Antifungal drugs (fluconazole, ketoconazole);
- Antimalarial drugs (mefloquine, chloroquine);
- Antidepressants (amitriptyline, imipramine, clomipramine, dosulepin, doxepin).
  - 1. **Permitted Concomitant Medications**

Preventive medication is not recommended before administration. However, when the subject experienced infusion reactions of grade 2 or less and could resume medication after treatment, the investigator could use premedication to prevent the recurrence of infusion reactions. Antihistamines, antipyretic analgesics, and glucocorticoids at doses of 10mg or less per day could be used to prevent the recurrence of infusion reactions. Pretreatment could be administered on the basis of clinical experience at each site. Systemic corticosteroids needed to control infusion reactions or immune-related adverse events had to be tapered for at least 2 weeks, and the immunosuppressive dose (equivalent to ≤10 mg of prednisone per day) had to be discontinued by the time of the next dose of study drug. In subjects allergic to diagnostic imaging contrast, steroids may be administered as prophylactic therapy. Subjects who were already taking hormone replacement therapy before enrollment could continue hormone replacement therapy after enrollment.

Subjects could receive supportive care. Supportive care could be combined with the following medications or related treatments: antibiotics, analgesics, hormones, psychotherapy, and any other symptomatic treatment necessary to provide optimal supportive care. Other investigational antineoplastic drugs or antineoplastic chemotherapy/endocrine/immunotherapy were not included in the definition of supportive care.

All treatments that the investigator considers no effects on study endpoints may be administered, such as unconventional therapies (e.g., herbal or acupuncture) and vitamin/mineral supplements. Bisphosphonates are allowed for subjects with bone metastases during the trial.

Palliative radiation therapy in a small area (<5% bone marrow region) will be allowed for subjects with uncontrolled pain of bone metastases after systemic therapy or topical analgesia, the premise is that the radiation field does not include the target lesion.

During treatment, granulocyte colony-stimulating factor (G-CSF) and other hematopoietic growth factors could be administered if the clinical presentation suggested or the investigator judged that it was necessary to treat acute toxic effects such as febrile neutropenia. Subjects were allowed to use erythropoietin on a long-term basis.

1. Study procedures and assessments
   1. Study procedures
      1. Stage 1: 3+3 dose escalation trial

According to the dose provided in the specification of anlotinib, three dose levels of 8 mg, 10 mg, and 12 mg are selected in the escalation trial (**Figure. 6.1-1**).

Three patients are enrolled and receive the initial dose of 8 mg anlotinib plus TQB2450 for safety assessment.

After follow-up of DLTs (1 Cycle, Day1-Day21), the following procedures are conducted:

(1) If no DLT is reported in three patients, the dose of anlotinib is escalated to 10 mg and combine with TQB2450, then three additional patients are enrolled.

(2) If DLTs are reported in one of three patients, three additional patients are enrolled at the same dose level (8 mg); if no additional DLT occurr in these three patients, the dose of anlotinib is escalated to 10 mg and combine with TQB2450, and three additional patients are enrolled; if DLTs are reported in ≥1 of three patients, the study will be terminated.

(3) If DLTs are reported in ≥2 of three patients, the study will be terminated.

When the dose of anlotinib is escalated to 10 mg and combine with TQB2450, three additional patients are enrolled for safety assessment. After follow-up of DLTs (1 Cycle, Day1-Day21), the following procedures are conducted:

(1) If no DLT is reported in three patients, the dose of anlotinib is escalated to 12 mg and combine with TQB2450, then three additional patients are enrolled.

(2) If DLTs are reported in one of three patients, three additional patients are enrolled at the same dose level (10 mg); if no additional DLT occurr in these three patients, the dose of anlotinib is escalated to 12 mg and combine with TQB2450, and three additional patients are enrolled; if DLTs are reported in ≥1 of three patients, the stage 2 study will be conducted at the dose of 8 mg anlotinib.

(3) If DLTs are reported in ≥2 of three patients, the stage 2 study will be conducted at the dose of 8 mg anlotinib.

When the dose of anlotinib is escalated to 12 mg and combine with TQB2450, three additional patients are enrolled for safety assessment. After follow-up of DLTs (1 Cycle, Day1-Day21), the following procedures are conducted:

(1) If no DLT is reported in three patients, the stage 2 study will be conducted at the dose of 12 mg anlotinib.

(2) If DLTs are reported in one of three patients, three additional patients are enrolled at the same dose level (12 mg); if no additional DLT occurr in these three patients, the stage 2 study will be conducted at the dose of 12 mg anlotinib; if DLTs are reported in ≥1 of three patients, the stage 2 study will be conducted at the dose of 10 mg anlotinib.

(3) If DLTs are reported in ≥2 of three patients, the stage 2 study will be conducted at the dose of 10 mg anlotinib.

Subjects enrolled in the 3+3 dose escalation trial receive the same examinations and follow-up to those in stage 2, as detailed in the flow chart in the stage 2 study of anlotinib.


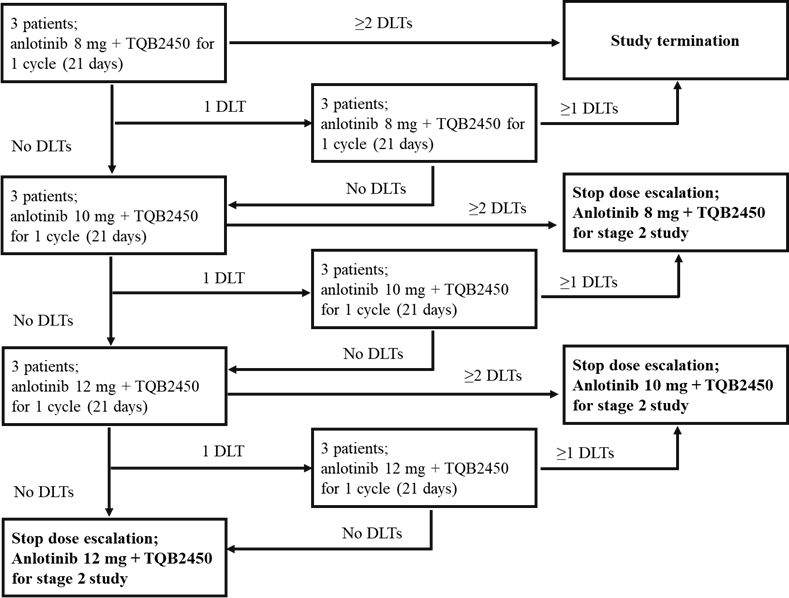


**Figure 6.1-1 Trial profile of the 3+3 dose escalation trial**

- - 1. Stage 2: multicenter, single-arm trial

All observation indicators and examination schedule will not be affected by duration of treatment discontinuation. All examinations (including imaging assessment) are conducted at protocol-specified treatment cycle, referring to the flow chart in the clinical trial of anlotinib.

| **Visit** | **Screening[1]** | | **Treatment period** | | | | **Post-treatment** | | | | **Follow-up of survival** | | |
| --- | --- | --- | --- | --- | --- | --- | --- | --- | --- | --- | --- | --- | --- |
|  |  |  |  |  |  |  | **Withdrawal** | **End-of-**  **treatment** | | **Before progression[2]** | | **After progression** | **After DCO[3]** |
|  | 1 | 2 | 3 | 4 | 5+ | 6+[4] | NA | NA | NA | | | NA | NA |
| **Visit windows** | -28-0 | -7-0 | ±3 | ±3 | ±3 | ±3 | ±3 | ±3 | ±7 | | | ±7 | ±7 |
| **Treatment cycles** | NA | NA | C1D7 | C1D21 | C3D21 | C5D21+ | NA | NA | NA | | | NA | NA |
| **Study weeks** | -4-0 | -1-0 | 1 | 3 | 9 | 15+ | NA | With 21 days after treatment discontinuation | Every 6 weeks | | | Every 6 weeks | Every 8 weeks |
| **Baseline characteristics** | | | | | | | | | | | | | |
| **Informed consent** | X |  |  |  |  |  |  |  |  | | |  |  |
| **Demographics** | X |  |  |  |  |  |  |  |  | | |  |  |
| **Inclusion/exclusion criteria** | X |  |  |  |  |  |  |  |  | | |  |  |
| **Enrollment ID** |  | X |  |  |  |  |  |  |  | | |  |  |
| **Physical examination [5]** |  | X | X | X | X | X | X |  |  | | |  |  |
| **Vital signs [6]** |  | X | X | X | X | X | X |  |  | | |  |  |
| **Medical history [7]** | X |  |  |  |  |  |  |  |  | | |  |  |
| **Compliance before enrollment [8]** |  | X |  |  |  |  |  |  |  | | |  |  |
| **Radiographic evidence of progression** | X |  |  |  |  |  |  |  |  | | |  |  |
| **Laboratory evaluations** | | | | | | | | | | | | | |
| **Hemanalysis [9]** |  | X | X | X | X | X | X | X |  | | |  |  |
| **Urinalysis [10]** |  | X | X | X | X | X | X | X |  | | |  |  |
| **Stool tests [11]** |  | X | X | X | X | X | X |  |  | | |  |  |
| **Blood biochemistry [12]** |  | X | X | X | X | X | X | X |  | | |  |  |
| **Coagulation function [13]** |  | X | X | X | X | X | X |  |  | | |  |  |
| **Thyroid function [14]** |  | X | X | X | X | X | X |  |  | | |  |  |
| **Tumor marker** |  | X | X | X | X | X | X |  |  | | |  |  |
| **12-ECG [15]** |  | X | X | X | X | X | X |  |  | | |  |  |
| **Myo-cardial enzymonram [16]** |  | X |  |  |  |  |  |  |  | | |  |  |
| **Echocardiography [17]** |  | X |  |  |  |  | X |  |  | | |  |  |
| **Hepatitis B, Hepatitis C, HIV [18]** | X |  | | | | | | | | | | | |
| **Pregnancy test [19]** |  | X |  |  |  |  | X |  |  | | |  |  |
| **Imaging assessment** | | | | | | | | | | | | | |
| **Imaging assessment [20]** |  | X |  | X | At the end of every two cycles (6 weeks±7 days) | | X |  | X | | |  |  |
| **Examination of brain lesions [21]** |  | X |  | X | At the end of cycle 3 (±7 days) and every 4 weeks thereafter (12 weeks±7 days) | | X |  | X | | |  |  |
| **Other clinical assessments** | | | | | | | | | | | | | |
| **ECOGPS** |  | X |  | X | X | X | X | X |  | | |  |  |
| **Neurological function evaluation and corticosteroid use** |  | X |  | X | At the end of cycle 3 (±7 days) and every 4 weeks thereafter (12 weeks±7 days) | | X |  | X | | |  |  |
| **Blood pressure monitoring [22]** | X | X | X | X | X | X | X |  |  | | |  |  |
| **Adverse events [23]** |  |  | X | X | X | X | X | X |  | | |  |  |
| **PD-L1 expression [24]** | X |  |  |  |  |  |  |  |  | | |  |  |
| Study drugs | | | | | | | | | | | | | |
| **TQB2450** |  | | 3-week cycle, given on day 1 in a cycle | | | |  |  | |  | |  |  |
| **Anlotinib** |  |  | 3-week cycle (2 weeks on and 1 week off) | | | |  |  | |  | |  |  |
| **Distribution/recycling of anlotinib** |  | X |  | X | X | X | X |  |  | | |  |  |
| **Concomitant treatments [25]** | X | X | X | X | X | X | X | X | X | | | X | X |
| **Drug compliance [26]** |  |  | X | X | X | X | X |  |  | | |  |  |
| **Follow-up of survival [27]** | | | | | | | | | | | | | |
| **Time to disease progression [28]** |  |  |  |  |  |  | every 6 weeks (±3 days) until disease progression or initiating other anti-tumor therapy (non-imaging PD patients) | | | | |  |  |
| **Time to death** |  |  |  |  |  |  |  |  |  | | | X | X |
| **Tomur treatment [29]** |  |  |  |  |  |  |  | X | X | | | X | X |

Note: All observation indicators and examination schedule will not be affected by duration of treatment discontinuation, but occasional changes in each wisit window due to holidays or other administrative reasons are allowed.

The window period of 3 days for screening indicators and examination must be completed before the initiation of the treatment cycle, for example, screening should not be repeated if performed within 7 days before the initiation of the first cycle of treatment. NA: not applicable.

1. Subjects must complete the screening visit within 28 days after imaging progression;
2. Subjects who withdraw from the study without objective progress will continue to be followed up until the date of objective progress occurred or data cut-off;
3. DCO: Data cut-off;
4. Subjects are visited every 6 weeks from V5;
5. Physical examination: Whole body system examination (facial features, integumentary system, lymph nodes, eyes, ears, nose, throat, abdomen, spine, extremities, and nervous system) are performed within 7 days before enrollment, at C1D7, C1D21, and thereafter at days 21 of every two cycles (odd numbered cycles), and at dropout visit;
6. Vital signs: Temperature, pulse rate, and respiratory rate are performed within 7 days before enrollment, at C1D7, C1D21, and thereafter at days 21 of every two cycles (odd numbered cycles), and at dropout visit; Blood pressure monitoring is described in [22];
7. Medical history: Pathological results, EGFR T790M mutation test report; history of tumor surgery, chemotherapy, radiotherapy, and other disease treatment;
8. Compliance before enrollment: Days between the first progression of the subject and enrollment;
9. Hemanalysis: Hemoglobin, red blood cells, white blood cells, neutrophil count, lymphocyte count, and platelets count are performed within 7 days before enrollment, at C1D7, C1D21, and thereafter at days 21 of every two cycles (odd numbered cycles), and at dropout visit, and at days 21 after treatment. If the neutrophil count ≤1×109/L or platelets (PLT) ≤50×109/L, additional examination (1 time/2-3 days) is required; weekly hemanalysis is required if there is a delay in dosing or dose adjustment due to hematological toxicity;
10. Urinalysis: Urine protein, glucose, occult blood (red blood cell and white blood cell), pH, and ketone bodies are performed within 7 days before enrollment, at C1D7, C1D21, and thereafter at days 21 of every two cycles (odd numbered cycles), and at dropout visit, and at days 21 after treatment; If the semi-quantitative method shows the levels of protein ≥2+ (e.g., urine test strip), 24-hour urine protein quantification should be performed;
11. Stool tests: Occult blood test is performed within 7 days before enrollment, at C1D7, C1D21, and thereafter at days 21 of every two cycles (odd numbered cycles), and at dropout visit.
12. Blood biochemistry: Liver function (TP, A, G, ALT, AST, LDH, ALP, TBil, DBil, and IBil), renal function (BUN, Cr, and UA), blood lipid tetrachoric (TC, TG, HDL, and LDL), electrolytes (K+, Na+, CL-, Ca2+, Mg2+, and P), lipase, amylase, fasting glucose, etc. are performed within 7 days before enrollment, at C1D7, C1D21, and thereafter at days 21 of every two cycles (odd numbered cycles), and at dropout visit, and at days 21 after treatment; During the drug administration, patients who develop early symptoms of liver injury (e.g., appetite loss, vomiting, right upper abdominal discomfort, malaise, etc.) before blood biochemical abnormalities should undergo blood biochemistry immediately. If ALT or AST is up to 3 times ULN or baseline value and total bilirubin up to 2 times the ULN or baseline value, the frequency of tests should be increased (recommended 1-2 times/week);
13. Coagulation function (PT, APTT, TT, Fbg, and INR) are performed within 7 days before enrollment, at C1D7, C1D21, and thereafter at days 21 of every two cycles (odd numbered cycles), and at dropout visit.
14. Thyroid function (T3, T4, FT3, FT4, and TSH) are performed within 7 days before enrollment, at C1D7, C1D21, and thereafter at days 21 of every two cycles (odd numbered cycles), and at dropout visit.
15. 12-ECG are performed within 7 days before enrollment, at C1D7, C1D21, and thereafter at days 21 of every two cycles (odd numbered cycles), and at dropout visit. If an abnormal ECG is identified, two additional confirmations must be performed (each 5 minutes apart and the QTc interval should be indicated);
16. Myo-cardial enzymonram is detected within 7 days before enrollment. This examination is supplemented only when symptoms such as precordial pain, palpitations, and ECG abnormalities are present;
17. Echocardiography is performed within 7 days before enrollment and at dropout visit. This examination is supplemented only when clinically meaningful ECG abnormalities occur during treatment;
18. Hepatitis B virus (HBV) and hepatitis C virus examination: HBV examination (HBsAg, HBsAb, HBeAg, HBeAb, and HBcAb) is performed and if the test result is abnormal, viral replication (HBA DNA) and hepatitis C virus antibody (anti-HCV) test should be conducted;
19. Pregnancy testing is limited to women of childbearing age, who are required to undergo a pregnancy test within 7 days before enrollment;
20. Imaging assessment: Including CT or MRI of the chest, abdomen, pelvis, and head. Baseline assessment of screening period tumors can be extended to within 3 weeks before treatment, and CT/MRI scans obtained before signing informed consent can be used for screening period tumor assessment if they meet the criteria; bone scans are required if suspected bone metastasis; no symptoms of cerebral hemorrhage need to be confirmed within 28 days before treatment in patients with stable brain metastases.

The imaging examination should be performed on chest and abdominal lesions under the same conditions as the baseline (layer thickness of the scan, use of contrast agents, etc.) on C1D7 and days 21 of every odd numbered cycle; Other lesions identified at baseline or new lesions suspected subsequently should also be examined at the appropriate time. Subjects should be promptly imaged when outgroup for any reason;

The window of the imaging schedule is ±7 days. An unplanned imaging examination may be performed when disease progression (e.g., symptoms worsening) is suspected. Subjects who discontinue treatment for reasons other than imaging-confirmed disease progression should perform an image evaluation at the date of end-of-treatment, and tumor efficacy evaluations are performed every 8-week follow-up thereafter until confirmed disease progression or initiating new tumor treatment is initiated.

1. Examination of brain lesions: Brain MRI and neurological function tests [19];
2. Blood pressure is measured by the subject himself/herself and recorded in the patient diary card. Blood pressure is tested at least 3 times per week for the first 2 cycles and followed up each day if the blood pressure is abnormal. In addition, blood pressure is measured again by the investigator at each follow-up visit, coffee, and tobacco intake are prohibited within 30 minutes before each blood pressure is measured. The measurement of blood pressure is taken in the sitting position with the arm at heart level after at least 10 minutes of quiet sitting, and each blood pressure measurement is taken on the same side;
3. AEs are recorded from the first dose of the study drug until at least 21 days after the last dose and follow-up until the AEs resolved or stabilized;
4. PD-L1 expression: Biopsy tissues are obtained from appropriate subjects for PD-L1 expression using immunohistochemistry.
5. Concomitant medications and therapy during the trial should be recorded, and the concomitant medication and therapy are recorded only in the case of novel or unresolved AEs associated with treatment if the subject discontinues treatment;
6. Study drug compliance: Drug doses, counts, and compliance for the previous cycle are calculated and recorded in CRF form at the first day of each cycle;
7. Survival follow-up: after discontinuation of trial treatment, survival status and subsequent antitumor therapy can be collected every 8 weeks by clinical or telephone follow-up until death;
8. Time to disease progression: for patients with non-imaging evidence of progression (intolerable and other conditions), imaging evaluation should continue every 8 weeks until disease progression, initiation of other oncologic therapy, death, or end of the study;
9. Tumor treatment is recorded during the follow-up period.
   1. Study assessments

For ethical reasons, examinations that already meet the study requirements prior to the subject's signing of the informed consent form may not need to be repeated.

- - 1. Subjects’ baseline characteristics
  1. Baseline characteristics: age, gender, Occupation, ethnicity, height, and weight;
  2. Smoking history (pack-years is defined as number of cigarettes per day /20×years)
  3. History of tumor treatment:
- Tumor operation: operation date and operation name;
- History of chemotherapy (including neoadjuvant and adjuvant therapy) and targeted therapy: name of drugs, treatment dose, treatment cycles, starting and ending time, and outcomes;
- Radiotherapy history: radiotherapy time, site, dose, and outcomes;
  1. History of comorbidities treatment: Comorbidities (diabetes, hypertension, etc) occurred within 5 years before signing ICF are recorded.
  2. Systematic physical examination: head and neck, heart and lung, abdomen, skin, superficial lymph nodes, and mental status;
  3. ECOG performance status: ECOG performance status is assessed as per Appendix 1.
  4. Vital signs: heart rate, blood pressure, body temperature, respiration.

Medical history and demographic characteristics are collected before enrollment, in order to confirm their eligibility. Any omissions or corrections in the medical history and demographic characteristics identified throughout the study must also be documented in subsequent original records.

Systematic physical examination, ECOG performance status and vital signs are required to be performed within 7 days before enrollment. Results within±7 days of study completion/withdrawal are allowed at the last visit. Reexamination is required if the examinations are outside the acceptable time window.

All vital signs should be measured in a resting state. If body temperature is measured more than twice in a day, the highest result is recorded.

Blood pressure monitoring: Blood pressure is monitored by the subject himself/herself recorded in the patient diary card. Blood pressure is monitored on alternate days for the first 4 weeks of treatment, at least every 1 week from week 5 to week 12, and at least every 4 weeks thereafter. If blood pressure is abnormal, it should be monitored daily; if blood pressure is normal, normal monitoring frequency can be resumed. Blood pressure is re-measured by investigator at each visit.

- - 1. Laboratory examination

| **Table 6.2-1 6.2.2 Laboratory examination** | |
| --- | --- |
| **Categories** | **Items** |
| Hemanalysis | WBC, ANC, lymphocyte count, red blood cells, Hb, platelet count |
| Urinalysis | red blood cell, white blood cell, and urine protein (analysis of 24-hour urine protein will be performed if necessary) |
| Stool tests | white blood cell, red blood cell, and occult blood |
| Blood biochemistry | ALT, AST, GGT, AKP, TP, ALB, TBIL, DBIL, GLU, BUN/Urea, Cr, UA, K^+^, Na^+^, Cl^-^, Ca^2+^, Mg^2+^, P, LDH, TC, TG, HDL, LDL |
| Coagulation function | INR, APTT, PT, FIB |
| Thyroid function | FT3, FT4, TSH |
| Lipase | Serum lipase (as appropriate) |
| Amylase | Serum amylase (as appropriate) |
| Tumor markers | Serum carcinoembryonic antigen (CEA) |
| Pregnancy | Serum HCG/urineHCG |
| Epidemiology | HbsAg (HBV DNA test for positive HbsAg), HBsAb, HBeAg, HBeAb, HBcAb, Anti-HCV, HIV |

During the screening period, the laboratory tests within 7 days before enrollment are allowed. Results within ±7 days of study completion/withdrawal are allowed at the last visit. Re-examination is required to comply with the requirements of the clinical study if the examinations are outside the acceptable time window.

- - 1. Cardiac function examination

12-ECG: During the screening period, the results of 12-ECG within 7 days before enrollment are allowed. Results within ±7 days of study completion/withdrawal are allowed at the last visit. Reports of 12-ECG must maintain traceability. The thermal sensitivity spectrum should clearly record the name and examination time of subjects. Copies of the bedside ECG spectra are reserved for reference but not included in statistical analysis.

Echocardiography: During the screening period, the results of echocardiography within 14 days before enrollment are allowed. Left ventricular ejection fraction is a mandatory item.

Myo-cardial enzymonram (CK, CK-MB) and troponin: During the screening period, the results of Myo-cardial enzymonram and troponin within 7 days before enrollment are allowed. This examination is supplemented only when symptoms such as precordial pain, palpitations, and clinically significant ECG abnormalities are presen.

- - 1. Imaging examination

Sites: During the screening period, head, neck, chest, and abdomen (including pelvic) are the mandatory sites that need to be examined. Other sites should be determined based on the trial protocol and clinical judgment.

Method: CT or MRI. MRI is used for head and neck (CT can be used as a substitute for MRI in subjects with contraindications for MRI). CT or MRI is used for chest and abdomen. Bone scan (ECT) is required if suspected bone metastasis.

Requirement for examination: The imaging examination should be performed under the same conditions as the baseline. Imaging examinations are performed as per RECIST v1.1 criteria to ensure a reasonable evaluation of efficacy.

Time requirements: During the screening period, ECT results of bone metastasis within 3 months before enrollment are allowed, while CT/MRI results of other sites within 28 days before enrollment are allowed. 6.2.4 Imaging within ±28 days of study completion/withdrawal are allowed at the last visit. After enrollment, all subjects were recommended to undergo ECT re-examination every 6 months. Subjects with baseline bone metastases were required to evaluate the bone metastasis site at each imaging evaluation. If investigator suspects new lesions based on clinical symptoms, confirmed ECT/MRI/CT are required.

- - 1. Detection of EGFR mutation, tumor mutation burden (TMB) and PD-L1 expression

Subjects who have received treatment with first or second-generation EGFR TKIs are required to provide a genetic testing report, proving the absence of primary or acquired *T790M* mutations. Subjects who have received treatment with third-generation EGFR TKIs are required to provide a genetic testing report, proving the positivity of EGFR mutations. For subjects with access to tissue samples, it is recommended to prioritize tissue-based *EGFR* mutation testing. Subjects without access to tissue samples may choose blood-based *EGFR* mutation testing using traditional gene testing (such as ddPCR, Arms-PCR, etc.) or high-throughput sequencing. T790M mutation and Exon 20 insertion mutation are key sites for testing.

PD-L1 expression is measured in suitable subjects from sub-centers with testing conditions.The selected subjects should meet the criteria for biopsy, and it is anticipated that sufficient tissue will be obtained for immunohistochemistry. Fresh tissue is divided into two parts, with one directly used for the immunohistochemistry of PD-L1, and the other prepared into a paraffin block for TMB testing.

- 1. Maximum tolerated dose and dose-limiting toxicity

MTD: Toxicity and safety are observed to determine the MTD of anlotinib when combined with TQB2450. MTD is defined as the highest dose when less than 33% of subjects experienced DLT.

DLT: DLT is defined as any one of the following toxicities occurring within the first 21 days of treatment (including events that started in the first cycle but were confirmed in the second cycle), as per NCI CTCAE 5.0.

1. Grade IV hematologic toxicity and following events:
2. ≥ CTCAE grade 3 febrile neutropenia (ANC<1000/ul; body temperature ≥38.5°C)
3. ≥ CTCAE grade 3 neutropenia lasting for >7 days
4. CTCAE grade 3 platelet count decreased lasting for >7 days, with signs of clinically significant bleeding or need for platelet transfusion
5. Grade II or higher liver and kidney dysfunction (lasting for >7 days after symptomatic treatment) and following events:
6. CTCAE grade 2 bilirubin increased, lasting for >7 days
7. ≥ CTCAE grade 3 bilirubin increased
8. CTCAE grade 3 AST or ALT increased, lasting for >7 days
9. CTCAE grade 4 AST or ALT increased
10. Grade III or higher non-hematologic toxicity (except for alopecias) and following events:
11. ≥CTCAE grade 2 pancreatitis
12. Cerebral hemorrhage, ≥grade 2 pulmonary hemorrhage
13. grade 2 abnormal coagulation or bleeding, lasting for >7 days
14. CTCAE grade 3 fatigue, lasting for >7 days
15. CTCAE grade 3 hypertension, hand-foot skin reaction, diarrhea, nausea, and vomiting that are not relieved by symptomatic treatment until the next treatment cycle.
16. CTCAE grade 3 abnormal laboratory examinations that are not relieved by symptomatic treatment until the next treatment cycle.

If one subject experiences toxicity that met the criteria for DLT, the study treatment was interrupted and follow-up was performed as described in the study procedures. Treatment can be discontinued due to any DLT. However, under the investigator and co-sponsor approval, subjects with DLT could receive the next lowest dose level after recovery.

- 1. Efficacy assessment
     1. Primary endpoint
- **Progression-free survival:**

PFS is defined as the time from enrollment to the first time of PD or death from any cause, whichever occurs first.

If the subject did not experience disease progression or death, PFS is defined as the date of the last confirmed progression-free. Subjects who discontinued for reasons other than disease progression (no subsequent tumor imaging assessment) and starting subsequent therapy after the trial were censored at the time of discontinuation or the initiation of subsequent therapy. In cases other than those described above, pre-planned sensitivity analyses will undertake to define PFS based on the date of confirming imaging progression events. New onset other tumor is not considered indicative of PD and is not censored as data.

If the image examination and assessment show PD, the date of PD is not defined as the imaging time of first signs of PD, but the time of definite imaging evidence of PD. If the PD is diagnosed with other clinical examinations, the date of PD is defined as the diagnosis time.

- - 1. Secondary endpoints
- **OS:** OS is defined as the time from enrollment to the date of death due to any cause. For the analysis of OS, data for patients who are alive will be censored for OS at the time of the last follow-up. Data for patients who were lost to follow-up will be censored for OS at the last date they were known to be alive. Censored OS is defined as the time from randomization to censor.
- **1-year PFS rate:** 1-year PFS rate is defined as the percentage of patients who have not progressed or died at 1 year.
- **1-year OS rate:** 1-year OS rate is defined as the percentage of patients who are still alive at 1 year.
- **Duration of response (DOR):** DOR is defined as the time from first documented evidence of complete response (CR) or partial response (PR) until PD or death, whichever occurred first. For the analysis of DOR, data for patients who discontinue study due to due to reasons other than disease progression (without subsequent imaging examinations) or hose who received post-trial treatment will be censored at the time of study discontinuation or the initiation of post-trial treatment. The occurrence of newly developed other tumors is not considered as a PD event, and will be not censored.
- **Disease control rate (DCR):** DCR is defined as the percentage of subjects with PR, CR, or stable disease (SD) lasting for ≥4 weeks.
- **Objective response rate (ORR):** ORR is defined as the percentage of subjects with confirmed tumor volume reduction to pre-specified values and maintenance of minimum time requirements (≥4 weeks), including subjects with a PR or CR. Objective responses are assessed per RECIST v1.1. Subjects must have at least one measurable lesion at baseline. The response was classified as CR, PR, SD, and PD according to RECIST v1.1.
  1. Safety assessment
     1. Safety management

Various management will be taken to ensure the safety of subjects in the study, including strict inclusion and exclusion criteria and close monitoring (hospitalization monitoring, follow-up, and out-of-hospital diary card recording and telephone follow-up).

Safety is analyzed in the safety analysis set (SAS).

- - 1. Adverse events

**(1)** **Pre-treatment events (PTE)**

PTE is defined as any adverse medical event that occurs prior to administration of any study drug in a subject who has signed informed consent, but which does not necessarily have to have a causality to the study.

Concomitant diseases and corresponding complications/abnormalities, new abnormalities that occur during the baseline evaluation are recorded as PTE. If PTEs aggravate abnormally after study drug treatment, investigator is responsible for determining whether it is an AE.

**(2) AEs**

AE is defined as any untoward medical occurrence in a subject who received study drugs, but which does not necessarily have to have a causality to treatment.

**(3) Observation and recording of AEs**

Any medical events that occurred from starting treatment until 28 days after the end of the study drug or the initiation of other anti-tumor therapy, regardless of its causality to study drugs, are considered to be an AE.

If laboratory abnormalities were part of a syndrome, the syndrome or diagnostic finding (e.g., anemia) rather than the laboratory finding (i.e., decreased hemoglobin) will be recorded.

The following information about AEs should be recorded, including time of AE occurrence (starting time) and recovery time (ending time), severity, the causality of AEs to study drugs, follow-up, outcomes, and managements.

AEs are followed-up until the disease is stable, normalized, and returned to baseline.

**(4) Grading the severity of AEs**

Grading the severity of AEs are assessed by the NCI-CTCAE version 5.0.

**(5) Causality assessment of AEs to drugs**

In this study, the relationship of AEs to TQB2450, anlotinib, and immune response will be assessed.

AEs was categorized as definitely-related, probably-related, possibly-related, possibly-unrelated, and definitely-unrelated.

Investigator(s) are required to assess the causality of AEs to study drugs, according to the following 5 criteria.

(1) Whether the administration time and the suspected AEs exhibit a reasonable relationship;

(2) Whether the suspected AEs fulfill the criteria for the typical reactions of the drug;

(3) Whether the suspected AEs can be explained by the effects of the combined drug, patient’s clinical condition, or other therapies;

(4) Whether the suspected AEs disappear or are mitigated after drug discontinuation;

(5) Whether the same AEs recurred after repetitive administration of the study drugs.

AEs will be calculated as the sum of definitely-related, probably-related, and possibly-related events.

|  | 1 | 2 | 3 | 4 | 5 |
| --- | --- | --- | --- | --- | --- |
| Definite | + | + | - | + | + |
| Probable | + | + | - | + | ? |
| Possible | + | + | ± | ± | ? |
| Unlikely | + | - | ± | ± | ? |
| Unrelated | - | - | + | - | - |

Note: +, Yes; -, No; ±, probably Yes or No; ?, unknown.

- - 1. Serious AEs (SAEs)

SAE is defined as any adverse event that results in any one of the following unanticipated events during the clinical trial period:

- 1. Death and life-threatening events
  2. Events leading to in-patient hospitalization or prolongation of existing hospitalization;
  3. Events causing persistent or severe disability;
  4. Events causing incapacity
  5. Events causing congenital anomalies/birth defects;

Events that should be not classified as SAEs:

1. Hospitalization due to the use of study drugs.
2. Hospitalization due to protocol-specified procedures (e.g., pathological tissue sampling); however, the event leading to in-patient hospitalization or prolongation of existing hospitalization due to these procedures should be classified as SAE;
3. Hospitalization for planned treatment of the disease。

If an SAE occurs, the clinical monitor and principal investigator are to be notified within 24 hours of awareness of the event. Meanwhile, investigator should fill out, sign, and date the SAE form, and immediately report it by fax or network to the National Medical Products Administration, Food and Drug Administration of the researcher's and sponsor region, Department of the National Health and Family Planning Commission, ethics committee of participating centers, clinical trial organizations of participating centers, and sponsor. In the event of fax or network failures encountered during the study, researchers may choose to use postal mail and are required to retain the corresponding express receipts and logistics records. Sponsor is required to regularly report to the investigators at the participating sites, the drug clinical trial institutions, and the ethics committee after receiving SAE form. Detailed report contact information is listed below.

| **Table 6.6-2：Contact information of SAE reporting sites** | | | | | |
| --- | --- | --- | --- | --- | --- |
| **Reporting sites** | **Address** | **Contact** | **Tel** | **Fax** |  |
| Chia Tai Tianqing Pharmaceutical Group Co., Ltd. | Building 9, No. 799-8, Xuanwu Avenue, Xuanwu District, Nanjing | -- | 025-68551889 |  |  |
| National Medical Products Administration, Drug Registration Division - Drug Research Division | No. 1 Beiluyuan, Exhibition Road North, Xicheng District, Beijing | -- | 0086-10-  68313344-1269 | 010-88363228 |  |
| National Health and Family Planning Commission of the People's Republic of China | No. 1 Xizhimenwai South Road, Xicheng District, Beijing | -- | 010-68792204 | 010-68792513 |  |
| Jiangsu Food and Drug Supervision Administration | No. 5 Gulou Street, Gulou District, Nanjing | -- | 025-83273701 | 025-83273714 |  |
| Jiangsu Cancer Hospital | No. 42 Baiziting, Nanjing City, Jiangsu | Ethics Committee | 025-83284707 |  |  |

- - 1. Definitely-related or suspected unexpected SAEs

As per “Standards and procedures for expedited reporting of safety data during drug clinical trials” issued by Center for Drug Evaluation (2018/1/27), co-organizer is responsible for the expedited reporting of definitely-related or suspected unexpected SAEs during the study. Investigator(s) is required to cooperate with the co-organizer in the data collection and discussion.

- - 1. AEs of special interest

AEs of special interest are required to immediately report to the sponsor. Sponsor is responsible for addressing potential new risks of clinical trial by appropriate approaches. Investigators are required to immediately report these following events to the sponsor. In all cases, investigators are required to report the event to the sponsor within 24 hours after learning of the event. Details events (regardless of its causality to study drug) are listed as following:

(1) Pregnancy in female subjects

Female subjects of childbearing potential are recommended to notify the investigators immediately if pregnancy was confirmed during the study or within 6 months after the last dose of study drug. Investigator(s) are required to complete the Clinical Trial Pregnancy Report Form and report to the sponsor or their designated personnel within 24 hours of being informed of a pregnancy occurrence and submit it to the sponsor or their designated personnel. Pregnancy events should not be documented in the electronic Case Report Form (eCRF) of AEs. Investigator should discontinue the treatment for pregnant subjects, provide advices, and discuss the risks of continuing the pregnancy and potential impacts on the fetus. Pregnant subjects will be monitored until the end of pregnancy. Investigator is required to submit the Clinical Trial Pregnancy Report Form when obtaining the latest information on the pregnancy process and outcomes.

(2) Pregnancy in female partner of the male subjects

Male subjects were informed by the informed consent that if their partner pregnant during the study period or within 6 months after the last dose of study drug, they should immediately notify the investigator. Investigator(s) are required to complete the Clinical Trial Pregnancy Report Form and report to the sponsor or their designated personnel within 24 hours of being informed of a pregnancy occurrence and submit it to the sponsor or their designated personnel. Investigator should provide advices, and discuss the risks of continuing the pregnancy and potential impacts on the fetus, meanwhile, discuss with her primary physician and/or obstetrician to discuss reasonable solutions. If subjects decide to continue the pregnancy, the investigator should collect detailed information about the course and outcome of the pregnancy in detail. Meanwhile, partners with pregnancy needs to sign an authorization form, allowing the use and disclosure of the health information related to this pregnancy and agreeing to undergo subsequent follow-ups for the pregnancy. After signing authorization, investigator is required to submit the Clinical Trial Pregnancy Report Form when obtaining the latest information on the pregnancy process and outcomes.

(3) Spontaneous abortion

Spontaneous abortion should be reported as SAEs (abortion has great significant significance assessed by sponsor) and recorded in the eCRF. Investigator(s) is required to report the SAE after aware of the occurrence of spontaneous abortion, as per the procedure in 6.4.3 section.

(4) Congenital anomalies/birth defects

Any congenital anomalies/birth defects in infants born to female subjects or female partners of male subjects who had taken study drugs should be reported as SAEs in the eCRF. Investigator(s) is required to report the SAE after aware of the occurrence of congenital malformations/birth defects, as per the procedure in 6.4.3 section.

- - 1. Management of common AEs

**6.5.6.1 Common AEs related to TQB2450**

Types and treatment recommendations of immune-related adverse events (irAEs) caused by TQB2450 are listed in Appendix 6.

**6.5.6.2 Common AEs related to Anlotinib**

**(1)** **Hand-foot skin reaction (HFSR)**

HFSR is a disease characterized by redness, marked discomfort, swelling and tingling of the palms or soles of the feet. It usually occurs in subjects treated with chemotherapy or targeted therapy

Grade 1: Minimal skin changes or dermatitis (e.g., localized erythema, hyperkeratosis, oedema) and painless;

Grade 2: Significant skin changes (flaking, blisters, swelling, and hyperkeratosis) and pain, interfering with daily life and activities.

Grade 3: Severe skin changes (flaking, blisters, bleeding, ulcers, oedema, and hyperkeratosis) with significant pain and limited personal self-care ability.

For subjects occurred grade 1 HFSR, supportive treatment is not required. For subjects occurred ≥grade 2 HFSR, the following supportive treatments are recommended: strengthen skin care, keep skin clean, avoid secondary infection; avoid pressure or friction; use moisturizers or lubricants, and topically apply lotions or lubricants containing urea and corticosteroids; topical antifungal or antibiotic therapy when necessary.

**(2)** **Hypertension**

In previous studies, anlotinib is associated with the frequent occurrence of hypertension and the incidence of grade 3 or higher hypertension was 13.61%. During this study, subjects are recommended to monitor blood pressure during treatment. In the event of a hypertensive event, the following managements are recommended:

Grade 1: Pre-hypertension (systolic blood pressure [SBP] 120-139 mmHg or diastolic blood pressure [DBP] 80-89 mmHg); No indication for antihypertensive drugs and blood pressure is monitored.

Grade 2: Stage 1 hypertension (SBP 140-159 mmHg or DBP 90-99g mmHg); Medical intervention is required. For recurrent or persistent (≥24 hours), symptomatic hypertension with >20mmHg SBP increases or >140/90 mmHg increase of previously normal range, monotherapy and blood pressure monitoring are required. Thiazide diuretic, angiotensin-converting enzyme inhibitors (ACEI), angiotensin II receptor blockers (ARB), beta-blocker, or calcium-channel blocker are commonly used.

Grade 3: Stage 2 hypertension (SBP ≥160 mmHg or DBP ≥100 mmHg); Medical intervention is required. Multidrug therapy is required, commonly using a thiazide diuretic combined with an ACEI, beta-blocker, or calcium-channel blocker.

Grade 4: Life-threatening (e.g., malignant hypertension, persistent neurological deficit, hypertensive crisis). Urgent treatments are required.

Hypertension emergencies: SBP >120 mmHg accompanied by acute or progressive target organ damage (e.g., Cerebral infarction, intracranial or subarachnoid hemorrhage, hypertensive encephalopathy, etc.); among them, the progressive or rapidly progressive hypertension based on chronic essential hypertension is the most common (about 40%-50%).

Hypertension urgencies: SBP >120 mmHg with no or minor organ damage.

In case of hypertensive crisis, subjects should discontinue the study treatment and withdraw from the study.

When subjects occur elevated blood pressure during vital sign measurements, a second measurement should be taken after a 15-minute rest. If the elevation is repeated and reaches the grade 2, it can be defined as an AE of hypertension. If the time of improvement from the first occurrence of hypertension to its reappearance is less than 1 week, it is identified as one AE and record the highest level of severity. The start time is defined as the time of the first hypertensive episode, and the end time is defined as the last outcome time of hypertension.

**(3) Proteinuria**

Proteinuria should be monitored closely for all subjects during the treatment period, particularly for subjects with a history of hypertension. For subjects with 2 consecutive results of urine protein (++), 24-hour urinary protein measurement should be performed. For subjects experienced proteinuria, doses are adjusted as per the principles listed in the dose adjustment section of the protocol.

**(4)** **Diarrhea**

Grade 1 to 2 diarrhea could be treated with supportive care at the onset (e.g., 4 mg loperamide orally followed by 2 mg every 2 hours until diarrhea is resolved).

**(5) Hyperlipidemia**

The management of hyperlipidemia should consider the patient's pre-treatment state and dietary habits. In addition to dietary control, hypercholesterolemia of grade 2 or higher (≥7.75 mmol/l) or hypertriglyceridemia of grade 2 or higher (≥2.5 times×UNL) should be treated with HMG-CoA reductase inhibitors (atorvastatin, etc.) or appropriate lipid-lowering agents.

**(6) Gastrointestinal bleeding**

The presence of gastrointestinal bleeding, including fecal occult blood (++) and above, vomiting blood, or fresh blood in the stool, should be treated actively and symptomatically. For upper gastrointestinal bleeding, fasting should be performed and treatment consisting of anti-acid, gastric mucosa protection, anti-bleeding (hemostatic cyclic acid, reptilase, etc.) and, if necessary, octreotide should be administered; for lower gastrointestinal bleeding, anti-bleeding, blood transfusion and supportive treatment should be given; for uncontrolled bleeding, assistance from a surgeon is required immediately.

**(7) Thyroid dysfunction**

The thyroid function should be monitored closely for all subjects. Symptomatic treatments (e.g., Euthyrox) are required when TSH ≥20 mU/L or any value of T3, T4, FT3, and FT4 is lower than the normal value. During the study period, if thyroid dysfunction is considered as an AE by the investigator, the beginning time of AE is defined as the time of the first laboratory abnormality of thyroid function.

1. **Data management and statistical analysis**
   1. **Data entry**

The data collected in this study will be conducted using the electronic data capture (EDC) system. Investigator(s) or designated representative should complete the source document/eCRF timely, accurately, completely, and normatively. Data entry must be performed by authorized personnel who are qualified in EDC operation training.

- 1. **Data verification**

Clinical research associates (CRA) have to conduct the source data verification to ensure the accuracy, completeness, consistency, and standardization of the data. Data administrators have to verify the data in the EDC system according to the verification protocol to ensure the accuracy, completeness, and standardization of the data. If any entries into the eCRF are incorrect or incomplete, any discrepancies will be noted in the EDC system by means of electronic data queries. The CRA and data administrators will ask the investigator or CRC to make appropriate corrections. The corrected data will again be reviewed for completeness and consistency.

- 1. **Data review and cleaning**

Data cleaning methods includes EDC system automatic verification, SAS program cleaning, and manual verification. The investigator or CRC will confirm the questionable data on the basis of manual or systematic queries and make appropriate corrections. The monitor or data administrator will judge whether the data is reasonable according to the answers to questions, data updates, and audit tracks. If the data queries are still unresolved, they will be noted in the EDC system again until the queries solving and the data is clean.

- 1. **Electronic signature**

After the data entry was completed, all data queries were closed, and the monitoring staff checked and the data administrator checked, the investigator is responsible for verifying the authenticity and accuracy of the data reported in the EDC system and making an electronic signature in EDC. If there was any data modification after signature, it was necessary to sign again. The investigator must sign and date the Investigator’s Statement of electronic signature before any study-related procedures. It is required to declare that an electronic signature has the same legal effect as a written signature.

- 1. **Database locking**

The database locking will be confirmed after the completion of data entry, SDV, data queries clearance, electronic signature, SAE consistency check, medical verification, and data verification report. The data administrators will carry out the database locking after completing the listing procedure. The locked database will be not opened in principle.

- 1. **Data transfer**

To ensure the evaluation and supervision by the State Food and Drug Administration and sponsor, all documents must be archived by the research center and treated as confidential material, including confirmed documents (those that can effectively check different records, such as medical records), signed informed consent forms, and detailed source records of drug allocation. Data will be stored under lock and key for 5 years. The ownership of all documents will belong to Chia Tai Tianqing Pharmaceutical Group Co., Ltd. Investigator(s) shall not provide it to any third party in any form without the written consent of the sponsor, except for State Food and Drug Administration.

- 1. **Data preservation**

To ensure the evaluation and supervision by the State Food and Drug Administration and sponsor, all documents must be archived by the research center and treated as confidential material, including confirmed documents (those that can effectively check different records, such as medical records), signed informed consent forms, and detailed source records of drug allocation. Data will be stored under lock and key for 5 years. The ownership of all documents will belong to Chia Tai Tianqing Pharmaceutical Group Co., Ltd. Investigator(s) shall not provide it to any third party in any form without the written consent of the sponsor, except for State Food and Drug Administration.

1. **Statistical analysis management**
   1. **Analysis datasets**

- Full Analysis Set: According to the intention-to-treat (ITT) principle, the efficacy analysis was performed on all cases that received at least one dose of the drug.
- Per-protocol set (PPS): All randomized subjects who received at least 6 weeks of medication, had at least one imaging assessment, meeting the trial protocol, with favorable compliance, did not use prohibited drugs during the trial, and complete CRF. No imputation of missing data. Efficacy analysis was conducted based on both the FAS and PPS populations.
- Safety analysis set: All randomized subjects have received at least one dose of the research drug and have a post-administration safety record were included in the safety analysis set. This data set was used for the safety analysis.
  1. **Statistical Analysis Plans**

All statistical analyses were based on SAS version 9.4 or above. The quantitative variables were listed as mean, standard deviation, median, quartile, minimum and maximum values. Qualitative variables are listed as frequencies and percentages, with two decimal places reserved for percentages. The Kaplan-Meier method was used to analyze time-to-event data.

**8.2.1 Sample size**

Phase I: Following the 3+3 principle of dose adjustment, a minimum of 3 and a maximum of 18 patients were expected to be enrolled.

Phase Ⅱ: The mPFS of TQB2450 plus anlotinib hydrochloride capsules was estimated to be 9 months, and the historical control group carboplatin combined with pemetrexed was set as 5 months. The two-sided test level α was 0.05, and the power level was 0.90. It was calculated that 54 subjects would need to be enrolled (considering that the loss to follow-up rate should be controlled at 20%). The study was expected to last 24 months, including 12 months after enrollment. At the end of the study, 32 end-point events were expected to have occurred.

**8.2.2 Distribution of subjects**

The enrollment and completion of the subjects and the reasons for early withdrawal were summarized. The situation of the population in each analysis set was summarized, and the list of subjects' early withdrawal and protocol deviation was listed.

**8.2.3 Demographic characteristics and baseline analyses**

The demographic and baseline characteristics of the subjects (ECOG score, smoking history, etc.) were descriptively analyzed. The mean, standard deviation, median, quartile, minimum and maximum values were listed for quantitative variables, and frequency and percentage were listed for qualitative variables. The demographic and baseline characteristics of the subjects were listed.

**8.2.4 Tumor treatment and concomitant disease treatment history**

Frequency and percentage were used to describe the treatment history of tumor and comorbidities.

**8.2.5 Safety analyses**

Safety analysis includes AEs/adverse reactions, serious AEs/adverse reactions, laboratory tests, vital signs, physical examination, ECOG score, 12-lead electrocardiogram.

- - - 1. **AEs**

Medical coding of adverse events/adverse reactions/immune-related adverse events was performed with the use of MedDRA 21.0. The occurrence of adverse events/adverse reactions/immune-related adverse events, serious adverse events/adverse reactions/immune-related adverse events, adverse events/adverse reactions/immune-related adverse events leading to withdrawal, and adverse events/adverse reactions/immune-related adverse events leading to death were summarized, the number and times of occurrence were listed, and the incidence was calculated. The incidence of adverse events/adverse reactions/immune-related adverse events and serious adverse events/adverse reactions were summarized according to system organ classification (SOC) and preferred terminology (PT), and the number and incidence of adverse events/adverse reactions were listed. The association and toxicity grade of AEs/AEs (NCI CTCAE v5.0) were summarized according to SOC and PT, and the number and incidence of AEs/AEs were listed. The list describes the details of the occurrence of each adverse event/adverse reaction for each subject.

- - - 1. **Laboratory tests**

The laboratory tests were classified according to clinical significance, namely normal, abnormal without clinical significance, abnormal with clinical significance or normal and abnormal. The laboratory tests were summarized descriptively by frequency and percentage using cross-classification table.

Lists describe occurrences of normal before treatment but abnormal after treatment, as well as subjects who were abnormal before treatment but worsened after treatment. The mean, standard deviation, median, quartile, minimum and maximum values of routine blood test, blood biochemistry, four coagulation tests, thyroid function, lipase and amylase were listed. The measured values and changes before and after treatment were described. Paired *t* tests were performed when necessary.

Describe the proportion of subjects with abnormal changes who have “abnormal clinical significance”, in which the investigator judges whether the abnormality has clinical significance.

| Type | Tests |
| --- | --- |
| Hemanalysis | WBC, ANC, LYM, RBC, Hb, PLT |
| Urinalysis | urine RBC, urine WBC, proteinuria, glucose in urine (proteinuria ≥ 2+, additional 24-h urine protein quantification is required within 1 week) |
| Stool tests | leukocyte, RBC, occult blood |
| Blood biochemistry | ALT, AST, GGT, ALP, LDH, TP, ALB, TBIL, DBIL, GLU, BUN/Urea, Cr, K^+^, Na^+^, Cl^-^, Ca^2+^, Mg^2+^, P, TC, TG, HDL, LDL |
| Six coagulation tests | INR, APTT, PT, FIB, TT, D-D dimer |
| Lipase | lipase |
| Amylase | amylase |
| Tumor marker | carcinoembryonic antigen |
| Pregnancy text | human chorionic gonadotophin |
| Epidemiological | HbsAgn (Additional HBV DNA if positive), HBsAb, HBeAg, HBeAb, HBcAb, Anti-HCV, HIV |

- - - 1. **Physical examination**

The physical examination was classified according to normal and abnormal, and the cross-classification table was used. Frequency and percentage were used to summarize the physical examination descriptively.

- - - 1. **Vital signs**

Means, standard deviations, medians, quartiles, and minimum and maximum values will be listed for vital signs, describing the measurements and changes before and after treatment. Paired *t* tests were performed when necessary.

- - - 1. **12-lead electrocardiogram**

The 12-lead electrocardiogram was classified according to clinical significance, namely normal, abnormal without clinical significance, abnormal with clinical significance or normal and abnormal. The 12-lead electrocardiogram was summarized descriptively by frequency and percentage using cross-classification table.

- - - 1. **Concomitant medications**

The WHODrug was used to code the combined drugs, and the use of combined drugs during the study period and the frequency of each drug were summarized. The details of the combined drugs are listed.

- - 1. **Efficacy analysis**
       1. **Analysis of primary efficacy endpoints**

PFS is defined as the time from enrollment to the first time of disease progression or death from any cause, whichever occurs first.

If the subject did not experience disease progression or death, PFS is defined as the date of the last confirmed progression-free. Subjects who discontinued for reasons other than disease progression (no subsequent tumor imaging assessment) and starting subsequent therapy after the trial were censored at the time of discontinuation or the initiation of subsequent therapy. In cases other than those described above, pre-planned sensitivity analyses will undertake to define PFS based on the date of confirming imaging progression events. New onset other tumor is not considered indicative of PD and is not censored as data.

If the image examination and assessment show PD, the date of PD is not defined as the imaging time of first signs of PD, but the time of definite imaging evidence of PD. If the PD is diagnosed with other clinical examinations, the date of PD is defined as the diagnosis time.

- - - 1. **Analysis of secondary efficacy endpoints**

OS is defined as the time from enrollment to the date of death due to any cause. For the analysis of OS, data for patients who are alive will be censored for OS at the time of the last follow-up. Data for patients who were lost to follow-up will be censored for OS at the last date they were known to be alive. Censored OS is defined as the time from randomization to censor.

One-year progression-free rate is defined as the probability of patients experiencing the endpoint events of disease progression from enrollment to one year. Endpoint events include disease progression, death, and withdrawal due to intolerance.

One-year survival rate is defined as the probability of patients experiencing the endpoint event of death from enrollment to one year.

DOR is defined as the time from the date of CP and PR until the date of disease progression. Subjects who discontinued for reasons other than disease progression (no subsequent tumor imaging assessment) and starting subsequent therapy after the trial were censored at the time of discontinuation or the initiation of subsequent therapy. New onset other tumor is not considered indicative of PD and is not censored as data.

DCR is defined as the percentage of subjects with PR, CR, or SD lasting for ≥4 weeks.

ORR is defined as the percentage of subjects with confirmed tumor volume reduction to pre-specified values and lasting for ≥4 weeks, including subjects with a PR or CR. Objective responses are assessed per RECIST v1.1. Subjects must have at least one measurable lesion at baseline. The response was classified as CR, PR, SD, and PD according to RECIST v1.1.

For PFS, OS, and DOR, Kaplan-Meier was used to estimate median survival and draw survival curves, and the 1-year disease progression rate and 1-year survival rate were also estimated. For ORR and DCR, the Clopper-pearson method was used to estimate 95% confidence intervals.

1. **Project management**
   1. **Information promulgation**

The present study will be registered and publicized in the Chinese Clinical Trial Registry after obtaining ethical approval from the responsible unit of the clinical study.

- 1. **GCP and ethics committee review and approval**

This clinical trial will be conducted in accordance with the Declaration of Helsinki (2013), the GCP promulgated by the NMPA and related regulations. The investigator will not begin any study subject activities until approval from the Clinical Research Ethics Committee of the Clinical Trial Team leader. Any modifications of the protocol during the clinical study should be declared and filed to the Ethics Committee.

Clinical investigators will follow all applicable regulations to protect subjects. The informed consent document used in the trials must be reviewed by the sponsor, approved by the ethics committee, and available for inspection.

- 1. **Informed consent**

Clinical investigators must make it clear to subjects that participation in clinical trials is voluntary, and they are completely free to refuse to enter the study or to withdraw from it at any time, for any reason, without incurring any penalty or withholding of treatment on the part of the investigator, and the rights and welfare of the subjects will be protected. The investigator must make the subject aware that participants in the trial and their personal information will be strictly maintained confidential. It is the responsibility of the investigator to provide an adequate explanation in understandable language of the aims, importance, anticipated benefits, potential hazards, consequences of the study, other available treatment options, and the rights and obligations of the subject under the Declaration of Helsinki. Adequate time will be provided to ensure that the subject has time to consider and discuss participation in the protocol and sign the informed consent.

Before participants enrolled in this study to perform any of the procedures required by the protocol, subjects must:

- Be informed of all contents of the study and all the contents and terms of the informed consent form.
- Be provided adequate time to consider and discuss participation in the protocol and sign the informed consent whether to participate in the study.
- Voluntary consent to participate in the study.
- Personally (or their legally authorized representative) signed and dated the informed consent form approved by the Ethics Committee.

If there are any updates or amendments to the informed consent form during the trial, written informed consent should be obtained using the updated/amended informed consent form for subjects continuing in the trial.

- 1. **Protocol** **modification**

Protocol modifications to ongoing studies if necessary must be submitted to the Ethics Committee again for approval before implementation. Approval of the ethics committee and the sponsor must be obtained before any changes can be implemented, except for changes necessary to eliminate an immediate hazard to trial subjects. Changes to eliminate an immediate hazard to trial subjects can be performed immediately, but the change must be documented in a protocol modification, reported to the ethics committee, and submitted to the appropriate regulatory agency within the required deadline.

If protocol modifications involve important new information of the investigated drug, the informed consent must be revised and sent to the ethics committee for approval, and then the subject's consent must be obtained again.

- 1. **Protocol deviation**

The investigator should document and account for all protocol deviations, and report all protocol deviations that may affect subject safety and data integrity to the ethics committee and the sponsor in a timely manner.

- 1. **Retention of document**

The investigator must maintain adequate and accurate records to enable the conduct of the study to be fully documented, including but not limited to the study protocol, study protocol modification documents, informed consent forms, and ethics committee and government approval documents.

- 1. **Study discontinuation**

1. Serious safety issue is identified by the investigators;

2. Efficacy too poor to continue the trial;

3. Major mistakes in the protocol make it difficult to evaluate drug effects;

4. Discontinuation of the study at the request of the administrative department.

Full discontinuation of the trial can be temporary or permanent and the discontinuation of the test should retain all test records for review.

- 1. **End of study**

Based on the statistical analysis of the trial, the investigation director summarizes the results objectively and in detail, and actively completes the end-of-trial study report to meet the unified regulatory requirements for the clinical review of new drugs. Each participating unit completes the report of the sub-center.

- 1. **Quality control and quality assurance**

To ensure the quality of the trial, the clinical study program will be discussed and established by the sponsor and the investigator before the trial formally begins. GCP training will be conducted for relevant investigators participating in the trial. Each research center must manage study drugs in accordance with the protocol and SOPs, including receipt, storage, distribution, retrieval, and destruction (if applicable).

The necessary measures will be performed in the design and implementation phases of the study to ensure that the data collected are accurate, consistent, complete, and credible, pursuant to the GCP guidelines. All observed results and abnormal findings in clinical trials should be verified and recorded in a timely manner to ensure the reliability of the data. The instruments, equipment, reagents, standards, and so on used in various tests in clinical trials should have strict quality management and ensure that they are working in normal conditions.

Clinical data will be entered into the EDC, and the supervisor verifies whether the information is complete and accurate and directs the research center staff to make the necessary corrections and additions.

The drug regulatory agency, ethics committee, sponsor’s monitors, and/or inspectors may conduct systematic inspections of clinical trial-related activities and documents to evaluate whether the trial is conducted in accordance with the trial protocol, SOPs, and relevant regulations and whether the trial data are recorded in a timely, truthful, accurate, and complete manner.

- 1. **Publication policy**

After the clinical trial, the investigator with the assistance of the sponsor and the statistical analysis unit conducts statistical analysis of the trial data using appropriate statistical methods, makes an objective summary based on the results of the trial, makes an objective evaluation of the safety of the drug based on the results, and finally provides a written summary report of the clinical study.

The sponsor and the principal investigator (research unit) should mutually agree on the submission and publication of papers of the trial. Any publication of the result by investigators will require pre-written consent from the sponsor. Subjects should be kept confidential at the time of publication.

1. **Participanting center and personnel.**
   1. **Participanting center, center number, and principal investigator (sorted by center number)**

| Participanting center | Center number | Principal investigator | Professional title |
| --- | --- | --- | --- |
| Jiangsu Cancer Hospital | 01 | Ji-Feng Feng | Chief physician |
| Jiangsu Province Hospital | 02 | Xin Zhao | Chief physician |

- 1. **Lead center and principal investigator**

Unit: Jiangsu Cancer Hospital

Address: 42 Bai ZiTing, Nanjing, Jiangsu Province

Postal Code: 210009

Responsible Person: Meiqi Shi

- 1. **Statistical analysis unit and responsible person**

Unit: Chia-tai Tianqing Pharmaceutical Co., Ltd.

Address: Building 9, No. 699-8, Xuanwu Avenue, Xuanwu District, Nanjing, Jiangsu Province

Postal Code: 210023

Responsible Person: Yi Wang

- 1. **Co-organizer and principal**

Unit: Chia-tai Tianqing Pharmaceutical Co., Ltd.

Address: Building 9, No. 699-8, Xuanwu Avenue, Xuanwu District, Nanjing, Jiangsu Province

Postal Code: 210023

Responsible Person: Long Qian

- 1. **Research progress**

Expected from August 2019 to August 2021.
